# Rhythms and Background (RnB): The Spectroscopy of Sleep Recordings

**DOI:** 10.1523/ENEURO.0235-25.2025

**Published:** 2026-02-03

**Authors:** Jonathan Dubé, Michael Foti, Stéphane Jaffard, Véronique Latreille, Birgit Frauscher, Julie Carrier, Jean-Marc Lina

**Affiliations:** ^1^Department of Psychology, Université de Montréal, Montreal, Quebec H3C 1J7, Canada; ^2^Center for Advanced Research in Sleep Medicine, Hop. Sacré-Cœur de Montréal, Montreal, Quebec H4J 1C5, Canada; ^3^Electrical Eng. Dep., École de Technologie Supérieure, Montreal, Quebec H3C 1K3, Canada; ^4^LAMA, Université Paris-Est Créteil Val-de-Marne, Créteil, 94010 CEDEX, France; ^5^Department of Neurology and Neurosurgery, Montreal Neurological Institute Hospital, McGill University, Montreal, Quebec H3A 2B4, Canada; ^6^Department of Neurology, Analytical Neurophysiology Lab, Duke University, Durham, North Carolina 27708; ^7^Centre de Recherches Mathématiques, Université de Montréal, Montreal, Quebec H3C 1J7, Canada

**Keywords:** arrhythmic activity, brain rhythms, EEG, NREM sleep, phase–amplitude coupling, spectral analysis

## Abstract

Nonrapid eye movement (NREM) sleep is characterized by the interaction of multiple oscillations essential for memory consolidation, alongside a dynamic arrhythmic 1/*f* scale-free background that may also contribute to its functions. Recent spectral parametrization methods, such as fitting oscillation and one-and-over-F and irregular resampling auto-spectral analysis, enable the dissociation of rhythmic and arrhythmic components in the spectral domain; however, they do not resolve these processes in the time domain. Instantaneous measures of frequency, amplitude, and phase–amplitude coupling (PAC) are thus still confounded by fluctuations in arrhythmic activity. This limitation represents a pitfall for studies of NREM sleep relying on instantaneous estimates to investigate oscillatory coupling. To address this limitation, we introduce “Rhythms and Background” (RnB), a novel wavelet-based methodology designed to dynamically denoise time series data of arrhythmic interference. This enables the extraction of purely rhythmic time series, suitable for enhanced time-domain analyses of sleep rhythms. We first validate RnB through simulations, demonstrating it accurately estimates the spectral profiles of individual and multiple oscillations across a range of arrhythmic conditions. We then apply RnB to publicly available intracranial electroencephalogram sleep recordings, showing that it provides an improved spectral and time-domain representation of hallmark NREM rhythms. Finally, we demonstrate that RnB significantly enhances the assessment of PAC between cardinal NREM oscillations, outperforming traditional methods that conflate rhythmic and arrhythmic components. This methodological advance offers a substantial improvement in the analysis of sleep oscillations, providing greater precision in the study of rhythmic activity critical to NREM sleep functions.

## Significance Statement

The Rhythms and Background (RnB) algorithm introduces a novel approach to signal processing in electrophysiology by disentangling rhythmic activity from the arrhythmic background at the time series level. RnB denoise brain rhythms from arrhythmic interference in both the time and spectral domains, providing clearer insights into cerebral oscillatory processes. This breakthrough has direct applications in studying brain connectivity and oscillatory dynamics during sleep. Additionally, its application in clinical populations where pathological changes in arrhythmic activity are common, such as neurodevelopmental and neurodegenerative disorders, will help to better understand abnormal oscillatory processes. By improving the accuracy of rhythmic signal analysis, RnB opens new avenues for understanding brain function and dysfunction in research and clinical settings.

## Introduction

Brain rhythms from electroencephalograms (EEG) and other electrophysiological techniques reveal the synchronized rhythmic activity from neural ensembles. These rhythms are often identified using the spectral peaks of the power spectrum (Buzsáki, 2004). A noteworthy case is nonrapid eye movement (NREM) sleep, which is characterized by cardinal rhythms such as sleep slow waves (SSWs; 0.5–4 Hz, delta), theta bursts (TBs; 6–10 Hz), sleep spindles (8–16 Hz, sigma), and sharp-wave ripples (100–200 Hz). NREM sleep rhythms are organized in complex wave sequences, with organized phase–amplitude coupling (PAC) across frequencies occurring locally and between remote brain regions (Klinzing et al., 2016; Helfrich et al., 2019). For instance, sleep spindles are organized by the phase of SSWs and occur preferentially during the transition period of SSWs toward their upstate. PAC between delta and sigma rhythms during NREM sleep is increasingly recognized as a crucial component of overnight memory consolidation, and the loss of this phase coupling in aging may contribute to impaired memory consolidation (Helfrich et al., 2018).

New evidence suggests that “arrhythmic” brain activity recorded from the EEG plays complementary roles to brain rhythms for NREM sleep functions (Helfrich et al., 2021). Arrhythmic activity recruits a broad range of frequencies and is usually expressed as a $1/f^{\beta }$ decay in the power spectral density (He, 2014). This “scale-free” arrhythmic component is usually estimated using the spectral slope (β) expressed in the log–log power spectrum. The steepness of β has been shown to change across sleep stages (Lendner et al., 2020; Helfrich et al., 2021; Niethard et al., 2021; Kozhemiako et al., 2022) and to show age-dependent changes, which can predict cognitive impairment (Finley et al., 2024; Hernandez et al., 2024). Importantly, changes in β may alter the spectral peaks’ characteristics in the Fourier domain typically attributed to brain rhythms. For instance, older humans exhibit higher fast-frequency power in their EEG spectrum due to a flatter β slope rather than increased fast oscillations (Voytek et al., 2015). Methods such as fitting oscillations and one-and-over-F (FOOOF) and irregular resampling auto-spectral analysis (IRASA) have been recently introduced to dissociate rhythmic and arrhythmic changes in the power spectrum, respectively, from their peaks and β characteristics (Wen and Liu, 2016; Donoghue et al., 2020). Rightly so, these methods are widely used across neuroscience (Donoghue et al., 2022; Ostlund et al., 2022).

At the signal level, arrhythmicity manifests as ubiquitous “desynchronized” scale-free fluctuations in electrophysiological recordings visible “in between” transient rhythmic oscillations and on which they are also superimposed (Helfrich et al., 2021). These scale-free brain fluctuations introduce noise into multiple signal processing methods by reducing the reliability of phase and amplitude narrowband estimates associated with brain rhythms (Jurkiewicz et al., 2021; Donoghue et al., 2022). As scale-free power dominates in lower frequencies, slow fluctuations create transient events that are erroneously detected as slow waves (Samaha and Cohen, 2022). Although methods such as FOOOF and IRASA are increasingly used to distinguish rhythmic and arrhythmic activity at the level of the power spectrum (Pani et al., 2022), no method currently addresses the disentanglement of rhythmic processes from the arrhythmic background at the signal level.

Here, we introduce Rhythms and Background (RnB), a wavelet-based method to extract a rhythmic time series in which the arrhythmic contribution is removed. Our method attenuates the $1/f^{\beta }$ contribution at the level of wavelet coefficients in the “timescale” framework, allowing the synthesis of a new time series, free from the arrhythmic background. This series may then be used in ulterior signal processing pipelines, such as for the detection and coupling analyses of purely rhythmic events. We validated RnB using realistic simulations and applied it to intracranial NREM sleep recordings to demonstrate that it improves the characterization PAC between delta and sigma rhythms.

## Materials and Methods

### From the spectral to the wavelet paradigm

The objective of this work is to extract as time series the rhythmic activity embedded in electrophysiological signals, in which the ubiquitous 1/f arrhythmic background is attenuated. We posit that the power spectrum of any signal can be decomposed into a scale-free background component and a distinct rhythmic contribution. On the Fourier spectral side, this can be formalized as follows:

$$\begin{array}{c} \Gamma (f)=P(f)\;e^{R(f)} \end{array},$$

where $P(f)=c/f^{\beta }$ accounts for arrhythmicity (with c as a positive constant and a “scaling exponent” β) that will govern the decay of the power spectrum, while R(f) represents oscillatory activity. By expanding the exponential, this model is equivalent to the following expression:

$$\begin{array}{c} \Gamma (f)\approx \;\frac{c}{\;f^{\beta }}+c\;f^{-\beta }R(f)+\cdots \end{array},$$

where the first term $c/f^{\beta }$ represents the scale-free background and the second term $c\;f^{-\beta }R(f)$ accounts for the rhythmic contribution. It is noteworthy that this contribution is defined as a product between two factors: a narrowband rhythmic process R(f) and the concurrent background $c\;f^{-\beta }$. The rhythmic term $\left(c\;f^{-\beta }R(f)\right)$ can thus be understood as a resonance within a neuronal dynamic system, in which rhythms simultaneously emerge from and are stabilized by background activity (Hahn et al., 2014; Tchumatchenko and Clopath, 2014). In the frequency domain (i.e., power spectrum), this interaction may give rise to a Gaussian peak, characteristic of oscillatory processes captured by spectral parameterization methods, provided that R(f) decays rapidly at low frequencies.

Spectral whitening approaches such as IRASA (Wen and Liu, 2016) proposed to estimate a data-driven spectral filter $Q(f)\approx P{\left(f\right)}^{-1}\approx f^{\beta }$ acting on Γ(f). In their method, the scaling exponent β is estimated through irregular resampling on an epoch-per-epoch basis and subsequently filtered with an epoch-wise filter $Q_{i}(f)$. Then, the rhythmic factor $e^{R^{*}(f)}$ is estimated by averaging the residual spectra across multiple epochs “*i*”:

$$\begin{array}{c} e^{R^{*}(f)}=\langle Q_{i}(f)\;\Gamma_{i}(f)\rangle \end{array}.$$

However, such methods operate only in the frequency domain, and the resulting “rhythmic spectra” cannot be used for time-resolved analyses.

RnB translates the model above into a wavelet framework, using the “timescale” decomposition of the signal. The upcoming section will give a detailed mathematical description of the algorithm; here, we provide a didactic and integrative overview.

**Figure 1.**
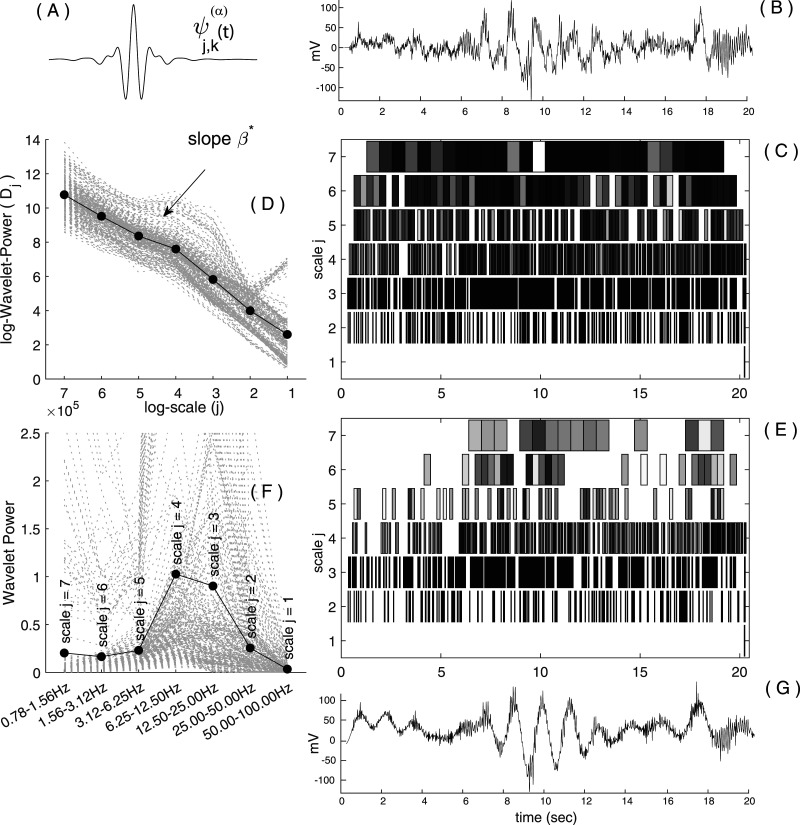
Timescale wavelet framework. ***A***, Example of a fractional spline wavelet $\psi^{\left(\alpha \right)}$ used by RnB for the timescale decomposition (panels ***C*** and ***E***). The fractional spline wavelet family is parameterized by the regularity index α, which defines its smoothness. Specific wavelets $\psi_{\;jk}^{\left(\alpha \right)}$are generated by applying scale (*j*) and time translation (*k*) transformations to $\psi^{\left(\alpha \right)}$. ***B***, Example of a 20 s trace of NREM3 sleep intracranial recordings from the anterior cingulate. ***C***, Timescale decomposition of the signal in ***B*** using the wavelet basis in ***A***. Each box represents a wavelet coefficient. $\left\langle s,\;\psi_{\;j,k}^{\left(4\right)}\right\rangle$ at a particular scale (*j*) and discrete time position $\left(k\;2^{j}\right)$. Real time is indicated on the *x*-axis (lower frequencies correspond to higher scales). Boxes account for 99% of the overall wavelet power. Lighter gray indicates higher coefficient amplitude (globally normalized). ***D***, Logarithmic wavelet power (*y*-axis) as a function of scales (*x*-axis), computed from 300 NREM3 sleep epochs. The linear slope β indicates the scale-free process across the wavelet coefficients. Each NREM3 sleep epoch is shown in light gray. and the dark bold line represents the average. The relationship between each scale (j) and its corresponding frequency interval is shown on the *x*-axis of the ***F*** subpanel. ***E***, Timescale decomposition of the RnB processed wavelet coefficients using the same signal as in subpanels ***B*** and ***C***, preserving scale-dependent rhythmic activity and attenuating the scale-free component (bold line) visualized in ***D***. Lighter gray indicates higher coefficient amplitude (globally normalized). Note that compared with the original timescale decomposition shown in ***C***, the rhythmic decomposition is lacunar because of the soft-shrinkage filtering (see Materials and Methods). ***F***, Wavelet power (*y*-axis) as a function of scale (*x*-axis), computed from the same 300 NREM3 sleep epochs shown in ***D***. For each *j* scale, the corresponding frequency intervals is shown on the *x*-axis (in correspondence with ***D***). Dashed gray lines indicate individual epochs, and the dark bold line represents the average. ***G***, Synthesized rhythmic signal using the filtered timescale coefficients shown in ***E***.

Wavelets are transient “little waves” of a specific scale localized in time (Fig. 1*A*). Like a “zoom” function, wavelets at small temporal scales capture fast transient fluctuations, whereas wavelets at larger scales capture slower oscillations. The wavelet coefficients are thus defined by correlating a signal with wavelets at different scales and time position. This results in a “timescale” decomposition of a signal, with wavelets coefficients expanded across scales and time (Fig. 1*B*,*C*). Both the scale-free (arrhythmic) background and the scale-dependent (rhythmic) component contribute to each wavelet coefficient in the timescale decomposition. The original contribution of RnB is to remove the scale-free contribution from each coefficient, preserving rhythmic activity in the coefficients.

This is achieved in two successive steps: first, the analysis of the original time series, and second, the synthesis of the rhythmic time series. During the analysis of the “original” time series, the scaling exponent (β) will be estimated from the wavelet power across all scales within each epoch (see Fig. 1*C*,*D* for NREM sleep). Because of their sensitivity to scaling properties, wavelets are uniquely suited to characterize the scale invariance inherent to electrophysiological recordings (Abry et al., 2019). This scale-free contribution will then be attenuated by removing the influence of β from all wavelet coefficients in the timescale expansion, preserving only scale-dependent rhythmic activity in the “filtered” representation (Fig. 1*E*,*F*). This procedure yields a “rhythmic time series,” providing a substrate for further analyses of oscillatory activity (Fig. 1*G*).

Mathematically, the scale-free contribution is removed from the coefficients via a fractional differentiation operation grounded in the properties of the spline wavelet basis (Unser and Blu, 1999), as detailed in the next section.

### “Mother wavelet,” wavelet family and timescale expansion of a signal

A “mother wavelet” $\psi^{\left(\alpha \right)}(t)$ is a localized transient function (finite duration), with oscillations centered around a characteristic frequency $f_{0}$ (Fig. 1*A*). Furthermore, this mother wavelet is characterized by a “regularity” parameter α which governs its behavior at low frequency $\left(∼\;f^{1+\alpha }\right)$. Intuitively, α reflects how smooth the wavelet is: low α values allow the wavelet to capture abrupt transients, whereas higher values provide smoother functions that more effectively represent recurrent oscillatory activity. Given such a “mother wavelet,” a collection of rescaled (scales $2^{j},\;j=1,2,\ldots$) and time-shifted (parameter k) wavelets is defined as follows:

$$\psi_{\;j,k}^{\left(\alpha \right)}(t)=\;\frac{1}{\sqrt{2^{j}}}\;\psi^{\left(\alpha \right)}\left(\frac{t-k\;2^{j}}{2^{j}}\right).$$

Any signal can thus be expanded using wavelets using a “timescale” decomposition (singular example on Fig. 1*B*,*C*) in terms of a weighted sum of discrete time translated (*k*) of successive scaled $\left(2^{j}\right)$ wavelets, up to a predefined scale $2^{J}$ as follows:

$$\begin{array}{c} s(t)=\;s_{r}(t)+\overset{J}{\underset{\;j=1}{\sum }}\;\overset{n_{j}}{\underset{k=1}{\sum }}\;w_{\;j,k}\;\psi_{\;j,k}^{\left(\alpha \right)}(t) \end{array},$$

where $n_{j}=2^{-j}N$ is the number of wavelet coefficients that describe the signal at scale $2^{j}$. Each coefficient $w_{\;jk}$ is obtained by correlating the signal with a scaled and shifted wavelet $\psi_{\;j,k}^{\left(\alpha \right)}$, and Figure 1*C* (timescale decomposition) gives a visual representation of the coefficients. For completeness, we highlight two points related to this wavelet expansion.

- *Localization of coefficients.* A wavelet coefficient at scale $\approx 2^{j}$ captures signal content around frequency $f_{j}=\;2^{-j}f_{0}$ localized around the temporal window of size $2^{j}$ and centered at $t=k\;2^{j}$. In other words, coefficients result from a localized bandpass filter in time and frequency. It is worth noting that, despite the temporal correlations present in the original signal, the wavelet coefficients of this discrete expansion are largely decorrelated, enabling localized processing.
- *Low-frequency residue.* The expansion also includes a residue [ $s_{r}(t)$ in Eq. 4] corresponding to stable slow activity not described by the wavelets. This large-scale approximation of the original signal results from a low-pass filter that complements the bandpass filters of the wavelet-based correlations. In practice, because signals are finite in duration, this residue also compensates for edge effects limiting reliable estimation of the largest scales (i.e., very low-frequency activity).

Thus, wavelet coefficients provide a timescale representation of the signal, whereas the residual captures an unavoidable low-frequency approximation in finite-length recordings.

### The RnB procedure

To extract a rhythmic time series, RnB will proceed in three steps using the analysis of an original time series: (1) estimate β per epoch from the log–log slope of wavelet power across scales; (2) attenuate the 1/f contribution on a sparse selection of the coefficients; and (3) synthesize the rhythmic time series from those coefficients in a suitable wavelet basis.

#### Estimation of the scaling exponent β in the wavelet domain

Rather than estimating β in the Fourier domain, we use the scaling of wavelet coefficient power given by Abry et al. (2019). Let $D_{j}=\log\;\left(1/n_{j}\sum_{k}w_{\;j,k}^{2}\right)$ be the average log power at scale *j.* A standard weighted least-squares regression of *D* computed over all logarithmic scales will reveal the expected linear trend of the scale-free process. Figure 1*D* illustrates this log–log linear regression on the wavelet coefficients from 300 epochs of NREM3 sleep recordings. Notice the linear slope indicating the presence of a scale-free process in the wavelet coefficients. This process contributes to the power of each coefficient in the timescale representation.

Formally, this “wavelet-based” scaling exponent is defined as follows:

$$\begin{array}{c} \beta^{*}=\frac{\sum_{\;j_{1}}^{\;j_{2}}\;n_{j}\;\sum_{\;j_{1}}^{\;j_{2}}\;j\;n_{j}\;D_{j}-\;\sum_{\;j_{1}}^{\;j_{2}}\;j\;n_{j}\;\sum_{\;j_{1}}^{\;j_{2}}\;n_{j}\;D_{j}}{\sum_{\;j_{1}}^{\;j_{2}}\;n_{j}\;\sum_{\;j_{1}}^{\;j_{2}}\;n_{j}j^{2}-{\left(\sum_{\;j_{1}}^{\;j_{2}}\;j\;n_{j}\right)}^{2}} \end{array}.$$

#### Attenuation of 1/*f* activity and denoising of wavelet coefficients

In analogy with the IRASA approach introduced in Equation 3, RnB adjusts wavelet coefficients in the “timescale” domain to account for ubiquitous, scale-free activity. To this end, we will use the fractional spline wavelets introduced by Blu and Unser (2000), whose regularity parameter α controls the smoothness of the basis and sets the expected scale law of the coefficients $\left(\beta^{*}\right)$. Unlike classical integer-order wavelets, this basis allows fractional orders, providing a natural way to decorrelate the $\beta^{*}$ exponent from the oscillatory content and thereby attenuate the 1/f^β*^ background.

For a signal with aperiodic exponent $\beta^{*}$ and using a wavelet analysis with $\alpha =a_{0}+\;(\beta^{*}/2)$, the decay of the wavelet coefficients follows $w_{\;j,k}\;∼2^{\;j(a_{0}+(\beta */2))}$ and combines the intrinsic regularity of the wavelet basis $\left(a_{0}\right)$ and the scaling exponent $\left(\beta^{*}\right)$. This scale-free process is visible in the coefficients from the timescale expansion of the initial signal (Fig. 1*C*), in which a power law is clear (Fig. 1*D*).

To remove the arrhythmic contribution, RnB will rescale the coefficients as $w_{\;j,k}^{*}\;∼2^{-j(\beta */2)}w_{\;j,k}$.This correction flattens the scale-free trend, leaving residual coefficients that primarily reflect rhythmic contribution as illustrated in Figure 1*E*.

In addition to the previous $1/f^{\beta^{*}}$ correction, the processing of the wavelet coefficients also involves a standard denoising, a “soft shrinkage” $S(w_{\;j,k})$ that will contribute to the sparsity of the wavelet representation (Donoho and Johnston, 1994):

$$S(w)=w\;\max\left(1-\frac{\lambda }{\left|w\right|},0\right)\;\;\mathrm{with}\;\;\lambda =\;\sigma \;\sqrt{2\ln\;N},$$

and the noise σ is estimated from the finest scale wavelet coefficients, $\sigma =\mathrm{median}(\left|w_{1,.}\right|)/0.6745$.

The “rhythmic” wavelet coefficients are therefore given by the following expression:

$$w_{\;j,k}^{*}=\;2^{-j(\beta */2)\;}S(w_{\;jk}).$$

#### Synthesis of the rhythmic time series

Since the previous processing removed the $\beta^{*}$ scaling from the wavelet coefficients, the rhythmic coefficients $w_{j,k}^{*}$ define a wavelet expansion using only $\alpha_{0}$ as follows:

$$s_{R}(t)\underline{\underline{\mathrm{def}}}\overset{J}{\underset{\;j=1}{\sum }}\;\overset{n_{j}}{\underset{k=1}{\sum }}\;w_{\;j,k}^{*}\;\;\psi_{\;j,k}^{\left(\alpha_{0}\right)}(t).$$

The regularity parameter $\alpha_{0}$ governs the wavelet smoothness of this synthesis and indirectly its effective temporal extent: larger α yield smoother wavelets with longer, more oscillatory temporal envelopes and narrower spectral bandwidth, whereas smaller α values produce shorter and more “impulsive” wavelets, with higher temporal but lower spectral resolution, reflecting the fundamental time–frequency trade-off (Blu and Unser, 2000; Abry et al., 2019).

We set $\alpha_{0}=4$ as a compromise between temporal and spectral resolution, suitable for NREM sleep rhythms. This value provides a sufficient frequency resolution to represent narrowband transients such as spindles while maintaining temporal sharpness for broader slow waves.

The resulting timescale representation and rhythmic time series (Fig. 1*E*,*G*) shows a marked attenuation of the arrhythmic component and highlight scale-specific rhythmic activity across multiple NREM3 epochs (Fig. 1*F*). Note that the rhythmic time series $s_{R}(t)$ omit the residual term $s_{r}(t)$ from Equation 4, which capture ultraslow nonlocalized fluctuations and whose removal improve robustness to very low-frequency artifacts and slow drifts.

### Spectroscopy of NREM sleep recordings

*S*pectroscopy refers to the systematic analysis of the intrinsic oscillatory content of a signal or observation, providing a basis for unambiguous interpretation. In RnB, the rhythmic signal is obtained from the synthesis of the denoised wavelet coefficients, in which the scale-free component is attenuated. However, because wavelet power is organized by dyadic scales (i.e., discrete frequency intervals), it lacks a fine quasicontinuous frequency resolution when considered as a power spectrum (as illustrated on Fig. 1*F*). However, the Fourier power spectra from the rhythmic signals will provide this finer description when averaged across epochs (akin to the IRASA method described in Eq. 3; Wen and Liu, 2016), yielding a reliable representation of dominant oscillatory activity.

**Figure 2.**
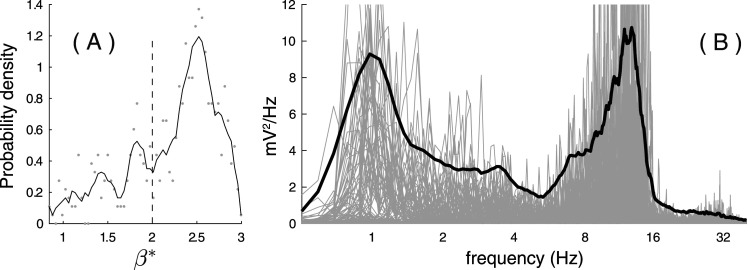
The “NREM sleep spectroscopy.” ***A***, The probability density distribution of the β from processing 300 20 s NREM3 sleep epochs (same epochs as in ***D*** and ***F***). ***B***, The Fourier spectra from the rhythmic signal of the same 300 20 s NREM3 sleep epochs after RnB processing. Light gray lines show individual epochs, while bold black is the average across all epochs.

In context of this paper, we will define the NREM sleep spectroscopy as the joint analysis of (1) the distribution of scale-free exponents $\left(\beta^{*}\right)$ across epochs, capturing the variations of the arrhythmic background (Fig. 2*A*) and (2) the Fourier spectrum of the rhythmic component from $s_{R}(t)$, describing its oscillatory activity (Fig. 2*B*). This provides an integrated framework for characterizing the organization of oscillations within their arrhythmic context across NREM sleep epochs.

### The RnB algorithm: code accessibility

In the following sections, we apply the RnB algorithm to simulations and intracranial NREM sleep recordings, demonstrating its validity through simulations and application using NREM sleep analysis. A guide how to use this algorithm on user-provided data can be found in a publicly available GitHub (https://github.com/michaelcfoti/RnB-Wavelet). The analyzed dataset for the figures presented in the following sections is available in the Extended Data. The RnB algorithm on the GitHub is available as a MATLAB package licensed under an Apache 2.0 license. The module supports MATLAB 2021a or later, with dependencies upon the “Signal Processing” toolbox. The package is openly developed and maintained. The project’s repository includes the codebase, a demo which allows for illustration use of the algorithm on publicly available and user-provided data and the documentation materials. This pipeline allows user-provided time series to extract a rhythmic time series and perform basic spectroscopy. Rhythmic time series are extracted as a matrix that can be used by the user to perform time-resolved analyses in other pipelines.

Two parameters need to be set to use the algorithm on user-provided data (see the *wRnBMain* script).

- The *j*_1_ and *j*_2_ parameters specify the range in which the scaling exponent β* is estimated (Eq. 5). This should be done across scales ranging from *j*_1_ (high frequencies) to *j*_2_ (low frequencies). The range of scales from *j*_1_ to *j*_2_ should cover a range in which the scale-free process and rhythms of interest are both present (see Fig. 1*F* for a correspondence between scales and frequencies). Importantly, there is a compromise between the scales to be used and the duration of the epochs in which the statistics are computed. The finest scales (high frequencies) should not include noisy activity, whereas the largest scales (low frequencies) are limited by epoch duration and edge effects. This can be manually tweaked in the RnB algorithm described in the following section.
- The parameter J refers to the largest scale (lower frequency) upon which the wavelet analysis/synthesis operations are performed to obtain the rhythmic signal.

These parameters can be changed according to the input data properties, and the documentation on GitHub allows the user to understand the impact of such changes. As default parameters used in this work, the scale ran from 1 to 9 to determine the arrhythmic $\beta^{*}s$ and the parameter J was set to 8.

### Simulation analyses of RnB

Simulations were conducted to assess RnB performance in characterizing background and rhythms. A set of eight second epochs were constructed, combining various scale-free backgrounds with transient 4 s oscillations. Considering fractional Brownian motion signals, the scale-free backgrounds were obtained by filtering white-noise time series to obtain a power law $1/f^{\beta }$ of the spectral power while keeping the Fourier phase random. To introduce variability in the background, each trial used a β scaling exponent randomly sampled around a predefined mean value, with a standard deviation of 0.1.

**Figure 3.**
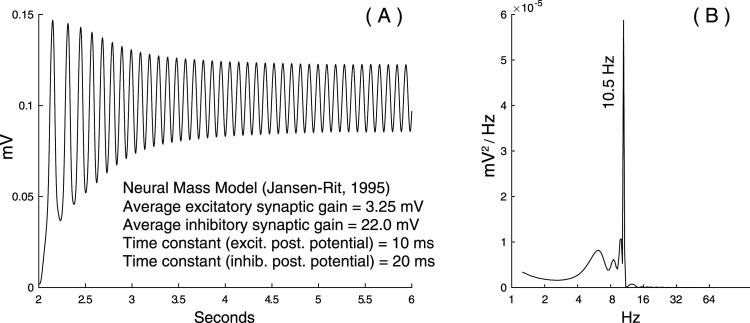
Methods: neural mass simulations. ***A***, A 4 s alpha oscillation produced by a standard neural mass model (with the original neurophysiological parameters onto the Figure). ***B***, The Fourier power spectrum of the model response (***A***). Notice that the alpha peak is accompanied with a lower-frequency content that corresponds to the first seconds of the simulated oscillation, which does not correspond to a “pure” harmonic oscillation.

**Figure 4.**
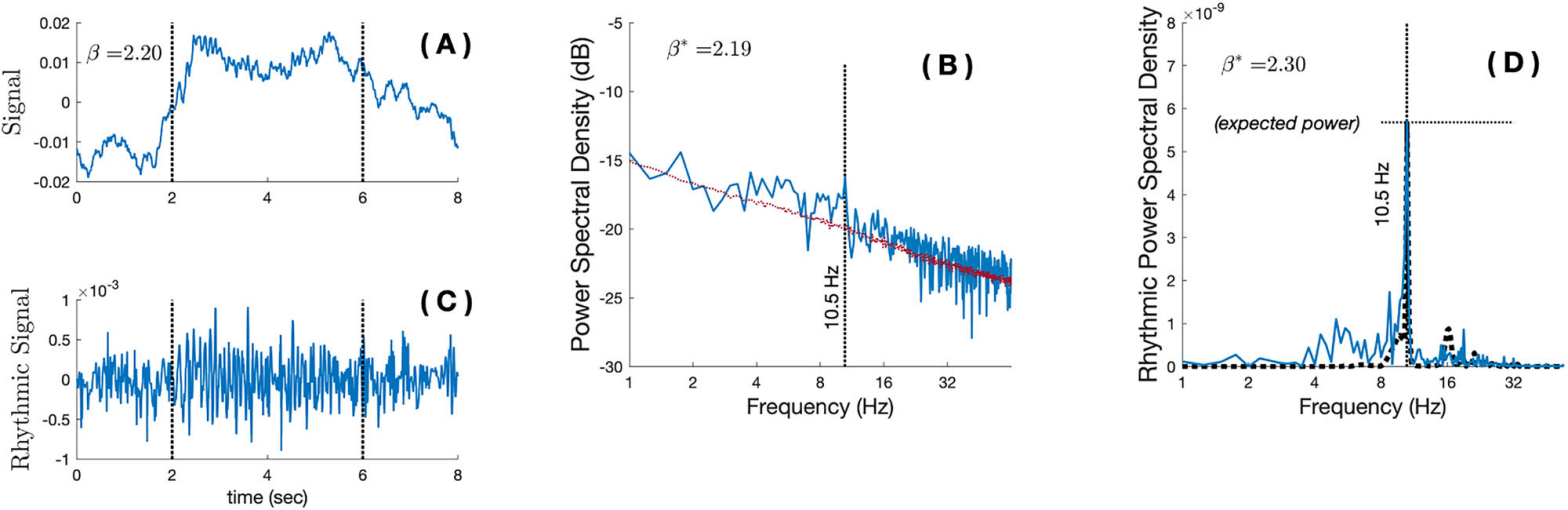
Methods: RnB estimations of neural masses simulations. ***A***, Example of a simulated time series of 8 s containing a mixture of a scale-free activity ( β=2.2; arrhythmic background) and the alpha oscillation from the neural mass model (4 s, 10.5 Hz rhythm). ***B***, The Fourier spectra of the single trial displayed in ***A***), mostly dominated by the arrhythmic background (1/*f* slope). ***C***, The rhythmic time series $s_{R}(t)$ resulting from the RnB processing of the time series presented in ***A***, synthesized from the filtered wavelet's coefficients. Note the attenuation of the arrhythmic drift between 0–2 s and 6–8 s and the preservation of a transient alpha rhythm between 2 and 6 s. ***D***, The Fourier spectra of epoch shown in ***C*** highlights a clear signature of the simulated oscillation that was added to the arrhythmic background, with a marginal “arrhythmic” contribution that is significantly attenuated compared with ***B***. The black dotted line is the original spectra of the simulated oscillation, without the addition of an arrhythmic background (“ground-truth”). The observed peak match with “expected power,” indicating that RnB adequately recovers the maximal power of the simulated oscillation in a single trial, after removing the arrhythmic background.

Four-second alpha oscillations were added to these scale-free backgrounds using a classical phenomenological neural mass model (Jansen and Rit, 1995; Pinotsis et al., 2012). This model simulates the dynamics of coupled inhibitory and excitatory neuronal populations interacting with a pyramidal cell. Examples of the temporal and spectral responses of this pure “rhythmic” model are presented in Figure 3. These neural mass responses were superimposed onto pure scale-free backgrounds to construct realistic time series (see Fig. 4*A* for an example).

Two sets of simulations were conducted to assess RnB's accuracy in estimating the maximal rhythmic amplitude of alpha and delta rhythms, as well as the scaling exponents:

1. The first set of simulations tested the robustness of RnB estimation for an alpha oscillation (10.5 Hz) simulated at 15 arbitrary amplitude levels within three arrhythmic background conditions (*β* = 1.7, 2.1, and 2.5).
2. A second set of simulations assessed the ability of RnB to perform accurate estimations in the context of two concurrent oscillations (delta at 3 Hz and alpha at 13 Hz). The delta oscillation varied across 15 incremental arbitrary amplitude levels, and the alpha amplitude remained constant.

For each condition (oscillation amplitude × background β), we first generated 300 pure scale-free epochs with β sampled around the target value (SD = 0.1). Oscillatory activity was then superimposed on 200 randomly chosen epochs per condition, leaving the 100 remaining ones as arrhythmic controls. To mimic a realistic scenario where rhythms may or may not be present, 60 epochs per condition were randomly selected among the total 300 for statistical analyses. RnB estimates of maximal rhythmic amplitude and scaling exponents were then extracted from these analyzed epochs.

### Intracranial NREM sleep analyses using RnB

From NREM sleep recordings, we investigated whether rhythmic time series exhibit cardinal NREM sleep rhythms, notably their spectral signature and interfrequency (delta–sigma) coupling. To do so, we first performed spectral analyses of the NREM sleep rhythmic time series produced by RnB. Second, we detected SSWs in the rhythmic time series and characterized how these events locally group and synchronize other rhythms in the theta and sigma frequency bands. Finally, we assessed whether delta–sigma PAC during NREM sleep differed between rhythmic and original time series.

#### Intracranial recordings

The intracranial recordings used in this work are extracted from a publicly available repository of intracranial EEG (iEEG) recordings in patients with epilepsy (MNI open iEEG atlas: https://mni-open-ieegatlas.research.mcgill.ca). From this multicentric database providing physiological activity in nonlesional tissues of the sleeping brain, we used the recordings of 16 patients. Analyses were performed on NREM2 and NREM3 sleep recordings, including all electrodes (sampling rate, 200 Hz) localized in the precuneus and the anterior cingulate, two key hubs of cortical synchronization for slow waves and sleep spindles (Murphy et al., 2009; Zerouali et al., 2014). All recordings were segmented into 20 s epochs. The duration of recordings in each region and sleep stage is described in Table 1.

**Table 1.** Dataset for the intracranial sleep recordings

Region	Sleep stage	# epochs	Total duration (min)
Precuneus	NREM2	967	322.33
	NREM3	951	317
Ant. cingulate	NREM2	821	273.66
	NREM3	809	269.66

Total number of 20 s epochs and total duration of each sleep stage are shown.

#### Spectroscopy of NREM sleep recordings

Each 20 s NREM sleep epoch was processed with the RnB algorithm to estimate the scaling exponent and extract a rhythmic time series. From RnB outputs, we estimated the distribution of scaling exponents and the average power spectral density separately in NREM2 and NREM3 sleep, what will be referred to as “NREM sleep spectroscopy” (Fig. 2). We compared these results to model fits from FOOOF, a standard spectral parameterization method (Donoghue et al., 2020).

For FOOOF analyses, we used the following parameters: an extraction of three peaks in the 0–45 Hz range, with fixed knee aperiodic modes (single slope). Aperiodic knees were initially parametrized in a subset of models but did not yield stable results and were therefore excluded from subsequent analyses.

#### Slow-wave detection in the NREM sleep rhythmic signals

We adopted a proof-of-concept approach to detect SSWs in the rhythmic time series from NREM3 sleep, to assess how they impacted the delta–sigma coupling during NREM sleep.

Rhythmic SSWs (rSSW) were identified in the rhythmic time series using a custom-made detector using region-adaptive frequency filtering. This filtering served to use the locally expressed oscillatory content in each region, in line with the recent approaches (Donoghue et al., 2022). This detector operates in three steps.

##### Step 1: Canonical slow-wave candidates and polarity in iEEG

We first used a standard intracranial slow-wave detector on the rhythmic times series, as defined by Ellenrieder et al. (2016). Briefly, candidates slow-wave half-waves were identified in the rhythmic time series after delta filtering (0.5–4 Hz) by detecting zero crossings. Only half-waves with a duration falling within physiologically plausible limits (125–1,000 ms) were retained. Because intracranial bipolar polarity varies according to neuronal geometry and therefore cannot be used to determine the phase of SSWs, we inferred rSSWs phase by comparing γ-band (30–80 Hz) power between successive half-waves; the half-wave exhibiting lower gamma power was labeled as the rSSWs downstate.

##### Step 2: region-adaptive rhythmic filtering

From the NREM sleep spectroscopy in each region (rhythmic time series), we defined a stage-specific delta bandpass filter centered on the main rhythmic delta peak which stood out locally during NREM3 (see Fig. 6 for relevant results). For the anterior cingulate, this range was 0.5–2.5 Hz; for the precuneus, it was 1–3 Hz. We then reapplied the same detection criteria as in Step 1 (zero-crossing identification, duration limits, and γ-based polarity determination) within this region-adaptive band to isolate events with maximal rhythmic content for each region and stage.

##### Step 3: conjunction of detected SW

The final rSSW inventory was defined as the conjunction of events detected in both passes. This procedure acted as a region-adaptative filter to lower the number of rSSWs retained from standard Step 1, in accordance with the frequencies observed in the NREM sleep spectroscopy of each region.

#### Original-signal SSW inventory

We also produced a second SSW inventory from the original NREM sleep recordings, using the standard intracranial detection criteria described in Step 1 above (von Ellenrieder et al., 2016). This set of SSWs detected in the original intracranial signal (without RnB processing) served to compare NREM sleep findings between the rhythmic time series provided by RnB and the original signal.

#### Windowing and classification of SSWs

For all analyses (spectroscopy, time–frequency, and PAC), we extracted 4 s windows centered on the maximal hyperpolarization peak of each SSW or rSSW.

As in Bouchard et al. (2021), we characterized the transition frequency of each SSWs and rSSWs by measuring the time delay (τ) between the down phase (hyperpolarization) and up phase (depolarization) maxima. The inverse of this delay was used to define, for each SSW, a transition frequency $f_{tr}=(1/2\tau )$. Then, we applied an unsupervised Gaussian mixture model (expectation–maximization algorithm) to the transition-frequency distribution in each region, classifying events into latent “switcher” classes: slow switchers (SlowS) with lower transition frequency and fast switchers (FastS) with faster transition frequency. Events in the rhythmic signal are referred to as rhythmic slow switchers (rSlowS) and rhythmic fast switchers (rFastS).

#### Spectroscopy analyses of rSSWs

In the first set of analyses, the spectroscopy of rSSWs was compared with an equivalent number of baseline periods, using 4 s NREM3 sleep time windows without rSSWs. Second, we directly compared the spectroscopy of rSlowS with that of rFastS, as SSWs with a slower transition frequency are known to better synchronize NREM sigma (≈13 Hz) rhythms (Chylinski et al., 2022).

#### Time–frequency analyses of rSSWs

To examine the timing of other NREM rhythmic activity around rSSWs, we used the rhythmic time series to perform time–frequency analyses around two distinct characteristic markers of rSSWs: (1) for standard rSSWs analyses, the peak of hyperpolarization was used as the central landmark consistent with previous studies (Cox et al., 2014); and (2) when specifically comparing rSlowS and rFastS, the window was centered on the zero-crossing point between hyperpolarization and depolarization, representing the midpoint of the transition phase for both types. This alignment ensures equivalence between the two types of switchers, as depolarization occurs more rapidly in fast switchers.

To assess the impact on rSSWs on other rhythms, we computed two types of scalograms, following Tallon-Baudry et al. (1999): (1) the induced power scalogram and (2) the phase-locked power scalogram. Both provide amplitude estimates in a time–frequency plane but differ in their computation. Induced power is obtained by averaging the amplitude of wavelet coefficients computed on a trial-by-trial basis. In contrast, phase-locked power is computed by first averaging the coregistered signal across rSSWs and then extracting the amplitude of the resulting wavelet coefficients (Tallon-Baudry et al., 1999). Phase-locked power thus isolates activity that is systematically aligned in phase across events, whereas induced power reflects the total event-related power regardless of phase consistency.

For all rSSWs analyses, we calculated the time–frequency wavelet transform within a 1 s window around each central landmark (indicated as *t* = 0 in Fig. 9). The scalograms were calculated using the continuous wavelet transform with the Morse analytic wavelet (default parameters, β=20 and γ=3). For visualization only, each scalogram is renormalized using an analytic variant of the Taeger–Kaiser power normalization and each display is individually rescaled between 0 and 1.

#### PAC analyses of rSSWs

To investigate how the rhythmic and original time series differ in their sensitivity to the PAC of delta and sigma rhythms during NREM sleep, we computed delta–sigma PAC from 4 s epochs centered on SSW epochs in the original time series and around rSSWs epochs in the rhythmic time series (rhythmic PAC, rPAC).

For SSWs, we used the phase φ(t) of the delta band (0.5–4 Hz) and the amplitude of the simultaneous sigma activity (10–16 Hz) from the Hilbert transform of the original signal. For rPAC, we considered the phase φ(t) and the amplitude of the simultaneous sigma activity from the Hilbert transform of the rhythmic time series preprocessed with *RnB*. For rPAC, we adapted the sigma frequency band as defined by the RnB spectroscopy in each region (Fig. 7; anterior cingulate, 8–14 Hz; precuneus, 11.5–14.5 Hz).

For SSWs or rSSWs, the PAC and rPAC are defined as follows:

$$\begin{array}{c} \mathrm{PAC}=\left|\frac{1}{T}\underset{t}{\sum }\;(A(t)-\langle A\rangle )\left(\;e^{i\;\varphi (t)}-\;\langle e^{i\;\varphi }\rangle \right)\right|=\;\;\left|\frac{1}{T}\underset{t}{\sum }\;A(t)\;e^{i\;\varphi (t)}-\;\frac{1}{T}\underset{t}{\sum }\;A(t)\;\;\frac{1}{T}\underset{t}{\sum }\;e^{i\;\varphi (t)}\right| \end{array},$$

where T is the epoch duration across which the summation is running and ⟨A⟩ and $\left\langle e^{i\varphi }\right\rangle$ are the mean amplitude and mean complex phase term across T. Subtracting these means removes spurious correlations caused by amplitude offsets or nonuniform phase distributions, providing a normalized measure that is robust to differences in overall signal power or phase bias (Cox et al., 2020). PAC values were further averaged by event types (rSlowS and rFastS) in each region (anterior cingulate and precuneus).

#### Detection of TBs associated with rSSWs in the rhythmic time series

The impact of TBs on delta–sigma PAC was investigated specifically in the precuneus (Jiang et al., 2019) because we identified a theta peak in the precuneus spectroscopy (Fig. 7). This was done because TBs were previously identified as precursors and potential modulators of sleep spindles (sigma) activity (Jiang et al., 2019). To do so, we adapted an existing NREM TB detection algorithm for the rhythmic time series. We used the specific θ-band from the NREM sleep spectroscopy of the precuneus (theta mode 7.02±2.4Hz in NREM3). The detected events needed to reach a peak above ±3 standard deviations of the average theta power, with start and stop times defined with ±1 standard deviation. The final TB inventory kept events with a duration between 0.4 and 1 s. We then extracted 4 s windows centered around rSSWs co-occurring with TBs (<500 ms; rSSWs + TBs events) and around rSSWs that did not (rSSW − TB events).

#### Statistics

We used statistical tests to determine the accuracy of RnB in our simulation and to evaluate the impact of the SSW switcher types on rhythmic power and rPAC. Data distribution was inspected with Shapiro–Wilk tests. Parametric tests (two-tailed) were used when normality held; otherwise, nonparametric equivalents were chosen. All statistical analyses were performed using SPSS 26. All statistical tests, including confidence intervals, are reported in the statistical table (Table 2).

**Table 2.** Statistical table

Test	Figure/panels	Dependent variable	Independent variable/contrast	Distribution	95%CI
Multiple regression	Figure 5*B*	α amplitude	α amplitude	Normal	[0.987 1.003]
	Figure 5*B*	α amplitude	Background condition	Normal	[0.011 0.013]
	Figure 5*B*	α amplitude	α amplitude *x* background condition	Normal	[−0.033 −0.027]
Multiple regression	Figure 5*C*	β exponent	Amplitude in (alpha oscillation)	Normal	[0.099 0.103]
	Figure 5*C*	β exponent	Scale-free regime	Normal	[0.399 0.402]
	Figure 5*C*	β exponent	Amplitude in *x* scale-Free interaction	Normal	[0.006 0.01]
Multiple regression	Figure 5*F*1	δ amplitude	Amplitude in (δ)	Normal	[0.84 0.89]
	Figure 5*F*2	α amplitude	Amplitude in (δ)	Normal	[−0.04 0.01]
	Figure 5*F*3	β exponent	Amplitude in (δ)	Normal	[0.024 0.029]
Mann–Whitney *U*	Figure 8*C*1	β exponent	rSlowS versus rFastS	Non-norm.	–
*t* test	Figure 8*D*1	Δ power	rSlowS versus rFastS	Normal	[2.84 11.82]
*t* -test	Figure 8*D*1	σ power	rSlowS versus rFastS	Normal	[0.65 11.91]
Mann-Whitney U	Figure 8*C*2	β exponent	rSlowS versus rFastS	Non-norm.	N/A
*t* test	Figure 8*D*2	Δ power	rSlowS versus rFastS	Normal	[−0.74 0.63]
*t* test	Figure 8*D*2	σ power	rSlowS versus rFastS	Normal	[−1.34 0.15]
Mann-Whitney *U*	Figure 10*A*	Slow switchers	PAC versus rPAC	Non-norm.	N/A
Mann-Whitney *U*	Figure 10*A*	Fast switchers	PAC versus rPAC	Non-norm.	N/A
Mann-Whitney *U*	Figure 10*B*	Slow switchers	PAC versus rPAC	Non-norm.	N/A
Mann-Whitney *U*	Figure 10*B*	Fast switchers	PAC versus rPAC	Non-norm.	N/A
Mann-Whitney *U*	Figure 10*A*	rPAC (anterior cingulate)	rSlowS versus rFastS	Non-norm.	N/A
Mann-Whitney U	Figure 10*B*	rPAC (precuneus)	rSlowS versus rFastS	Non-norm.	N/A
Mann-Whitney U	Figure 10*C*	TBs (precuneus)	TB versus all SW	Non-norm.	N/A

Statistical results from all experiments, including the structure of the data, the statistical test used, the sample size, effect size estimates (with 95% confidence intervals), and the *p* values.

Linear regressions were used to assess the relationship between simulation parameters and RnB estimates (estimation of maximal rhythmic amplitude and arrhythmic scaling exponents). Simulation parameters (arrhythmic beta, rhythmic amplitude) and their interactions were entered as predictors of RnB outcomes in the SPSS Statistics 26 software.

For intracranial data of NREM sleep, bilateral *t* tests (with 1,000 bootstraps to obtain bias-corrected confidence intervals) or nonparametric Mann–Whitney *U* tests investigated the impact of the switcher type (rSlowS vs rFastS) on the NREM sleep spectroscopy (arrhythmic beta and rhythmic power). For rhythmic power, we integrated power in adaptive frequency bands for identified peaks in the NREM sleep spectroscopy of the anterior cingulate and precuneus (delta, 0.5–4 Hz in both regions; sigma, 8–14 Hz in the anterior cingulate, 11.5–14.5 Hz in the precuneus; theta, 5–9 Hz in the precuneus; Fig. 6).

Mann–Whitney *U* tests were used to test the difference in delta–sigma PAC between the original and rhythmic time series separately for both types of switchers in the precuneus and in the anterior cingulate. PAC and rPAC difference between switchers were also investigated separately in each region. Finally, the impact of TB on PAC differences was investigated in the preceding classification (rSSWs + TBs vs rSSWs − TBs) in the precuneus.

## Results

We used realistic simulations to evaluate RnB performance in estimating rhythmic (oscillation amplitude) and scaling (β) parameters. As a first illustrative example of RnB application, we considered a simulated alpha (10.5 Hz) oscillation embedded in an arrhythmic background (Fig. 4). The initial signal (Fig. 4*A*) exhibited a clear arrhythmic drift, which was evident in its Fourier spectrum (Fig. 4*B*). After RnB processing, the rhythmic signal shows a clear transient oscillation, and the arrhythmic drift is removed (Fig. 4*C*). The spectral analysis of the rhythmic signal reveals a clear alpha peak in the rhythmic spectral density (Fig. 4*D*).

**Figure 5.**
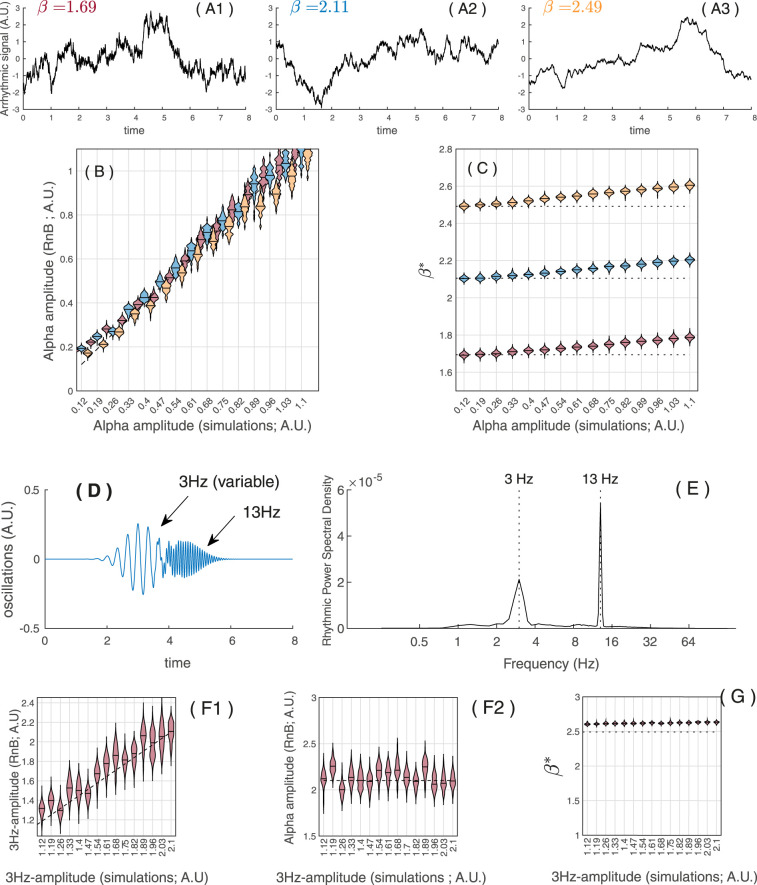
Simulation results: RnB estimation of rhythms and background. ***A*1*–A*3**, Examples of simulated “pure” arrhythmic time series with three different scale-free β exponents (β in each condition, red, 1.69; blue, 2.11; yellow, 2.49); 300 simulations were generated for each arrhythmic condition. Simulated alpha oscillations (10.5 Hz) at 15 fixed amplitude levels (*x*-axes on the ***B*** and ***C*** subplots) were added to a subset of simulations in each of the arrhythmic conditions (see Materials and Methods for details). ***B***, Median and kernel density of RnB estimated alpha amplitudes (*Y*-axis) for the 15 fixed simulated alpha amplitude levels (*X*-axis), in each arrhythmic condition (red, *β* = 1.69; blue, *β* = 2.11; yellow, *β* = 2.49). ***C***, Median and kernel density of RnB estimated β exponents (*Y*-axis) for the 15 fixed simulated alpha amplitude levels (*X*-axis), in each arrhythmic condition (red, *β* = 1.69; blue, *β* = 2.11; yellow, *β* = 2.49) ***D***, Example of two concurrent simulated oscillations (delta at 3 Hz and alpha at 13 Hz). Simulated delta oscillations were varied at 15 incremental amplitude levels (*x*-axes in F1 and F2 subplots), whereas the delta oscillation was fixed. ***E***, Power spectral density (after RnB processing) of the dual-oscillation simulation model showed in ***D***. ***F*1**, Median and kernel density of RnB estimated delta amplitude (*y*-axis) for the 15 fixed delta amplitude levels (*x*-axis), concurrent with an alpha oscillation. ***F*2**, Median and kernel density of RnB estimated alpha amplitude (*y*-axis) for the 15 fixed amplitude levels of the simulated delta oscillations (*x*-axis), concurrent with alpha oscillation. ***G***, Median and kernel density of RnB estimations of the β exponents (*y*-axis) for the 15 fixed amplitude levels of the simulated delta oscillations (*x*-axis), concurrent with an alpha oscillation.

Regression analyses then examined how varying the amplitude of simulated alpha oscillations (15 amplitude levels × 3 arrhythmic background conditions; Fig. 5*A,B*) predicted RnB alpha amplitude estimates*.* RnB alpha amplitude scaled significantly and positively with the simulated alpha amplitude (*b* = 0.99; *p* < 0.0001), accounting for nearly all variance in RnB outputs (*R*^2^ = 0.98; Fig. 5*A*). A small amplitude × background interaction was observed (*t* = 8.58; *p* < 0.0001; Δ*R*^2^ < 0.01), indicating a weaker relationship in the background condition with higher β values (Δ*b* = −0.05; *p* < 0.0001). Importantly, the association between simulated and measured alpha amplitude remained strong and significant in all three background conditions (slopes ranging from *b* = 0.94 to 0.99; all *p* < 0.0001).

We next tested whether varying the amplitude of simulated alpha oscillations predicted RnB scale-free background (β) estimates across the same 15 amplitude levels × 3 background conditions (Fig. 5*C*). In contrast to the strong effects on alpha amplitude estimates, RnB β estimates scaled only modestly with simulated alpha amplitude (*b* = 0.10; *t* = 91.1; *p* < 0.001), which accounted for <1% of the variance in β estimates (*R*^2^ < 0.01; Fig. 5*C*). A small amplitude × background interaction was again observed (*t* = 9.47; *p* < 0.001; Δ*R*^2^ < 0.01), indicating a stronger positive association in the high-β background condition (Δ*b* = 0.01; *p* < 0.001). Across all models, background condition remained by far the dominant predictor of RnB β estimates (*t* = 663.3; *p* < 0.0001; *R*^2^ = 0.99).

Finally, we evaluated RnB estimates in simulations with two concurrent oscillations (delta at 3 Hz and alpha at 13 Hz; Fig. 5*D*–*G*). Simulated delta amplitude varied across 15 incremental levels, while alpha amplitude remained constant. RnB estimated delta amplitude scaled strongly and positively with simulated delta amplitudes (*b* = 0.87; *p* < 0.0001; *R*^2^ = 0.85; Fig. 5*F*1). In contrast, *RnB* estimated alpha amplitude was unaffected by simulated delta amplitude (*b* = −0.01; *p* = 0.27; Fig. 5*F*2). By comparison, β estimates increased significantly with higher simulated delta amplitude, explaining ∼31% of the variance of β estimates (*b* = 0.03; *p* < 0.001; *R*^2^ = 0.31; Fig. 5*G*).

Overall, RnB provided reliable estimates of both rhythmic amplitudes and scaling exponents across single and concurrent oscillations (alpha and delta). Two minor caveats should be noted: (1) RnB scaling exponent (β) estimates are slightly overestimated in the presence of high amplitude oscillation (especially delta), and (2) rhythmic amplitude estimates (alpha) are slightly attenuated in high-β arrhythmic conditions. Together, these patterns indicate that while rhythmic and arrhythmic components are disentangled, minimal residual cross-influence persists.

### The spectroscopy of sleep recordings

We now demonstrate the potential of RnB to characterize NREM sleep rhythms using intracranial recordings of NREM sleep. Our analyses focused on the anterior cingulate and the precuneus, two key regions involved in NREM delta (slow waves) and sigma (sleep spindles) rhythms (Murphy et al., 2009; Zerouali et al., 2014).

**Figure 6.**
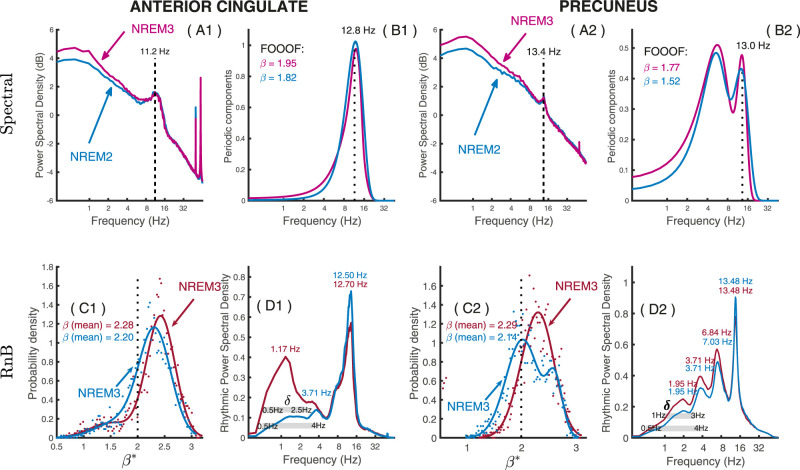
Results: RnB application to NREM sleep recordings. Results from spectral factorization using FOOOF (top row) and spectroscopy analyses using *RnB* (bottom row) for NREM sleep recordings in the anterior cingulate (two left columns) and precuneus (two right columns). ***A*1, *A*2**, Standard spectral density per region in NREM2 (blue) and NREM3 (pink) sleep, highlighting a significant arrhythmic 1/*f* background alongside spectral peaks. ***B*1, *B*2**, Residual periodic power is estimated by FOOOF in NREM2 and NREM3 sleep. Dashed vertical lines indicate spectral peaks. ***C*1, *C*2**, Probability density of scaling exponents (per epoch) as assessed by RnB in NREM2 (red) and NREM3 (blue) sleep. ***D*1, *D*2**, Spectral analysis from the RnB rhythmic time series, highlighting distinctive peaks in each region. Thick horizontal lines in the delta band indicate the frequency bands to be used for the detection of SSWs in the rhythmic time series.

For illustrative purposes, we first show how the NREM sleep power spectrum can be parameterized using FOOOF (Donoghue et al., 2020). As expected, results from FOOOF highlight that intracranial recordings of NREM2 and NREM3 sleep spectrum are characterized by different scaling exponents, with steeper average arrhythmic slope in NREM3 compared with NREM2 in both anterior cingulate and precuneus (β shown on Fig. 6*B*1,*B*2). FOOOF also reveals sigma peaks in the average NREM spectrum in both stages and regions, with an additional theta peak in the precuneus during NREM2 and NREM3 sleep (Fig. 6*B*1,*B*2).

Next, we assessed whether the rhythmic time series extracted by RnB provided a reliable spectral representation of NREM sleep. Spectroscopy analyses were performed on rhythmic time series from NREM2 and NREM3 sleep, considering power spectral density and the distribution of scaling exponents. Like FOOOF, the distributions of scaling exponents β assessed by RnB showed higher scaling exponents in NREM3 as compared with those in NREM2 sleep, for both anterior cingulate and precuneus (β distributions shown respectively on Fig. 6*C*1,*C*2). Furthermore, higher rhythmic delta power was observed in NREM3 compared with that in NREM2 sleep in the anterior cingulate and precuneus, although the specific frequency bands varied between the two regions (Fig. 6*D*1,*D*2). In the anterior cingulate, a single low-frequency delta peak (1.17 Hz) predominated in NREM3 as compared with NREM2 sleep, whereas two delta peaks (1.95 and 3.71 Hz) were found in the precuneus during NREM3 and NREM2 sleep. A rhythmic theta peak (≈7 Hz) was also found specifically in the precuneus during NREM2 and NREM3 sleep. Additionally, rhythmic sigma peaks were found in both regions, with slightly faster frequencies in the anterior cingulate (12.5 Hz) as compared with the precuneus (13.5 Hz) and lower maximal power in NREM3 compared with NREM2. The spectroscopy of NREM recordings is thus concordant with expected standard NREM delta and sigma rhythms, in addition to theta rhythms in the precuneus.

### rSSWs modulate rhythmic and arrhythmic activity

Given that rhythmic time series showed higher delta activity during NREM3 compared with lighter (NREM2) sleep in both recorded regions, we detected SSWs in the rhythmic time series from NREM3 sleep using spectroscopy-adapted frequency criteria (see Materials and Method), which we named “rhythmic sleep slow waves” (rSSWs). We wanted to assess how they modulate other rhythms during NREM3 sleep using time–frequency analyses (time-resolved level) and spectroscopy (spectral level).

**Figure 7.**
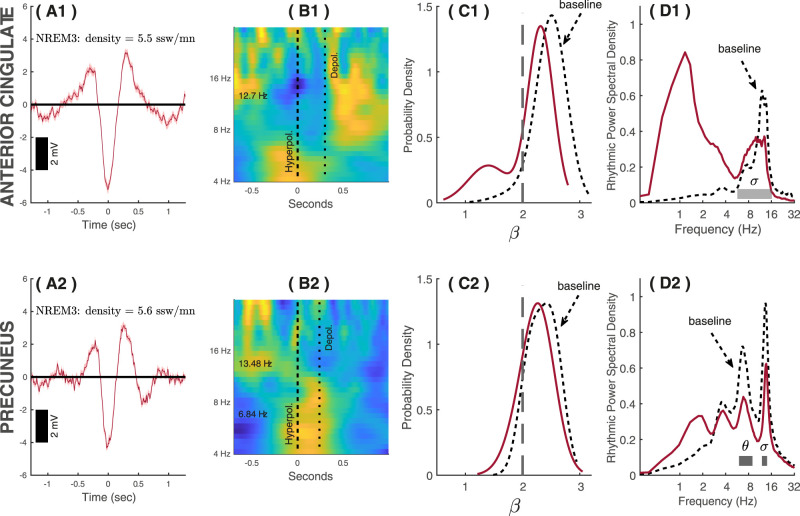
Detection of rSSWs from the anterior cingulate and precuneus during NREM3 sleep. ***A*1, *A*2**, Average waveforms of rSSWs during NREM3 sleep in the anterior cingulate (top) and precuneus (bottom). ***B*1, *B*2**, Time–frequency analyses (induced power) of the rhythmic time series associated with rSSWs coregistered with respect to the hyperpolarization phase. Red/yellow indicates higher normalized power than blue. ***C*1, *C*2**, Distribution of scaling exponents across epochs with rSSW (continuous lines) and epochs without rSSW (dashed lines). ***D*1, *D*2**, Rhythmic power density with rSSWs (continuous lines) and without rSSWs (dashed lines).

Detection rates of SSWs and rSSWs were lower in the rhythmic time series than in the original signal during NREM3 sleep (≈5 rSSW/min vs ≈14 SSW/min), which is expected since arrhythmic fluctuations inflate SSW counts in raw data. rSSW average waveform retained the expected biphasic shape of SSWs, with fast-frequency suppression during the downstate (Fig. 7*A*1,*A*2). Interestingly, time–frequency analyses of the rSSW in NREM3 indicate that they were associated with sigma activity in their depolarization period, predominantly in the anterior cingulate (Fig. 7*B*1,*B*2).

The spectroscopy of rSSWs was compared with baseline NREM3 sleep epochs (without SSWs). The probability density distributions showed lower scaling exponents for epochs with rSSWs than for baseline epochs, particularly in the anterior cingulate (Fig. 7*C*1,*C*2). Rhythmic power density showed higher delta and lower sigma rhythmic power for epochs with rSSWs compared with epochs without rSSWs in both regions, although the anterior cingulate showed stronger stage-related difference in rhythmic delta (Fig. 7*D*1,*D*2). Rhythmic sigma peak was wider in the anterior cingulate as compared with the precuneus. The theta peak previously observed in the precuneus also showed lower power during rSSWs as compared with the baseline, suggesting that theta activity in the precuneus may not be specifically linked with rSSWs.

### The spectroscopy of SSWs differs according to transition frequency

SSWs can be categorized as slow or fast switchers, depending on the duration of their transition frequency, the inverse of their transition period between their hyperpolarization and depolarization phase (Bouchard et al., 2021). Sigma rhythms are preferentially nested in the transition of SSWs with longer transition periods (slow switchers) as compared with fast switchers during NREM sleep (Chylinski et al., 2022). We thus evaluated in the rhythmic time series how slow switchers group sigma activity during NREM3 sleep.

**Figure 8.**
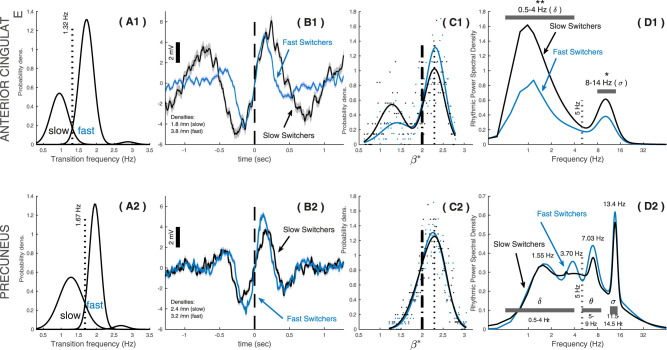
The spectroscopy of rSSWs in function of their transition frequency during NREM3. rSSWs can be classified according to the duration of their transition period between hyperpolarization and depolarization (i.e., transition frequency). Slow waves with a shorter transition period between their hyperpolarization and depolarization peaks are considered “fast switchers” (rFastS in the rhythmic time series), whereas rSSWs with a longer transition periods are considered “slow switchers” (rSlowS in the rhythmic time series). ***A*1, *A*2**, The probability density distributions of the transition frequencies of rSSWs in the anterior cingulate (***A*1**) and precuneus (***A*2**), highlighting a bimodal distribution of transition frequency in both regions. The thresholds to cluster rFastS and rSlowS are shown as dashed vertical lines in both regions. ***B*1, *B*2**, Average waveforms of rSlowS and rFastS epochs in the rhythmic time series. The dashed lines show the transition midpoint. ***C*1, *C*2**, Probability density distributions of the scale-free exponents for rSlowS and rFastS in each region. ***D*1, *D*2**, Spectral power density of the rhythmic time series associated with rFastS and rSlowS in NREM3 for both regions. The horizontal shaded bars indicate local frequency bands (delta, theta, sigma) used in statistical analyses (*t* tests). Bilateral significance for each band is shown separately using stars (*p < 0.05; **p < 0.01). Significant differences in rhythmic power between rSlowS and rFastS were found in the anterior cingulate only.

We categorized each rSSW detected in the rhythmic time series from NREM3 sleep as a rhythmic slow switcher (rSlowS) or a rhythmic fast switcher (rFastS; see Materials and Methods). Depending on their transition frequency, we clustered rSSWs between rSlowS and rFastS and found a probability distribution skewed toward rFastS in both regions (Fig. 8*A*1,*A*2). The transition frequency separating rSlowS and rFastS was ∼1.32 Hz in the anterior cingulate and 1.67 Hz in the precuneus, indicating a slower transition from hyperpolarization to depolarization in the anterior cingulate (see Fig. 8*B*1,*B*2 for examples).

The spectroscopy of rSlowS differed from rFastS particularly in the anterior cingulate. In this region, the scaling exponents of rSlowS showed a distribution with lower median values compared with rFastS (Fig. 8*C*1; standardized *Z*, −4.5; *p* < 0.001 on Mann–Whitney standardized *U* test). In the precuneus, no difference between the distributions of scaling exponents was found between slow and rFastS (Fig. 7*C*2; *p* > 0.05). In the anterior cingulate (Fig. 8*D*1), rSlowS showed significantly higher rhythmic delta power (*t* test, *p* < 0.001) and higher rhythmic sigma power (*t* test, *p* < 0.05) as compared with rFastS. In the precuneus, rhythmic power spectral density did not significantly differ between rFastS and rSlowS in either considered frequencies (all *t* tests, *p* > 0.05; Fig. 8*D*2).

### RSlowS and rFastS regroup differently transient sigma and theta rhythms

To evaluate how other rhythms were regrouped in time by the transition of rSSWs, we examined induced and phase-locked power in time–frequency analyses from the rhythmic time series. Induced power reflects the overall amplitude from the average of the wavelet coefficients across all events. In contrast, phase-locked power is the wavelet amplitude computed on the average signal coregistered to a common landmark. Phase-locked power thus provides an indication of how consistently a given event or rhythm occurs in phase at a specific point across coregistered event (here, relative to the transition midpoint of rSSWs), whereas induced power captures increase or decreases in oscillatory energy regardless of the precise phase alignment of the signal. Previous work shows that sigma rhythms tend to occur specifically in phase during the transition from hyperpolarization to depolarization of SSWs and especially in waves with a slower transition such as rSlowS (Chylinski et al., 2022). We thus hypothesized that rSlowS would exhibit stronger phase-locked sigma power during their transition when compared with rFastS.

**Figure 9.**
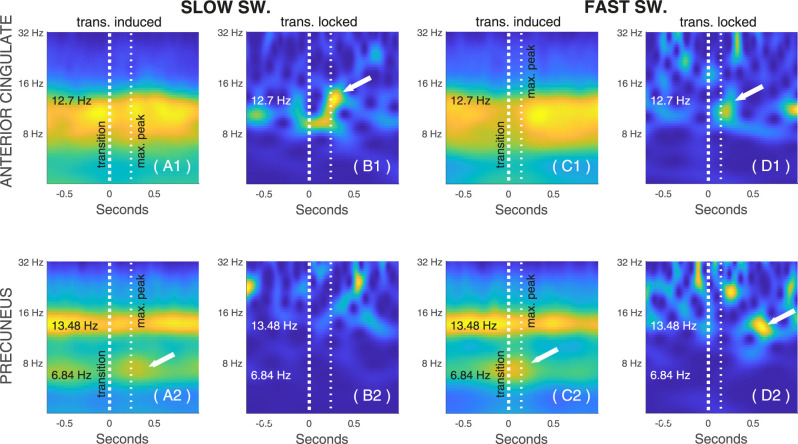
Time–frequency analyses of rSSWs in function of their transition frequency during NREM3 sleep. Time–frequency maps (scalograms) of rSSWs in the anterior cingulate (top) and precuneus (bottom), coregistered to the transition midpoint. Slow switchers (longer transitions; left panels ***A, B***) are contrasted with fast switchers (shorter transitions; right panels ***C, D***). Induced power is shown in ***A*** and ***C***, phase-locked power is shown in ***B*** and ***D***. Vertical dashed lines mark the transition (bold) and subsequent depolarization peak (light dash). White arrows highlight transition-locked sigma in the anterior cingulate (***B*1, *D*1**) and precuneus (***D*2**) and transition-induced theta in the precuneus (***A*2, *C*2**). Each panel is individually normalized for visualization (red, high power; blue, low power).

Induced sigma power was observed around the transition of both rSlowS and rFastS, in the anterior cingulate (Fig. 9*A*1–*C*1) and in the precuneus (Fig. 9*A*2–*C*2). As expected, rSlowS in the anterior cingulate further showed noticeable phase-locked sigma power during their transition, reaching maximal power around the depolarizing peak (Fig. 9*B*1). rFastS in the anterior cingulate also exhibited some phase-locked power around the depolarizing peak (Fig. 9*D*1), although less pronounced than for rSlowS. In the precuneus, phase-locked sigma power was not strongly modulated around the maximal depolarization peak, for either rSlowS or rFastS. However, some phase-locked sigma emerged ∼400 ms later after the depolarization peak of rFastS in the precuneus (Fig. 9*D*2).

In the precuneus specifically, induced theta power was observed with the transition of rSlowS and rFastS (Fig. 9*A*2,*C*2). However, theta power was not sufficiently synchronized across events to yield a robust phase-locked power signature around the transition of either rSlowS (Fig. 9*B*2) or rFastS (Fig. 9*D*2).

### The rhythmic time series highlights coupled delta and sigma rhythms in NREM3 sleep

We compared delta–sigma coupling from the rhythmic time series versus the original time series. PAC was assessed from SSWs (original iEEG signal) and from rSSWs (rhythmic time series), referred to as rhythmic PAC (rPAC). We hypothesized that rSlowS and rFastS events would show higher PAC than in the original signal, due to minimized arrhythmic interferences in the rhythmic time series.

**Figure 10.**
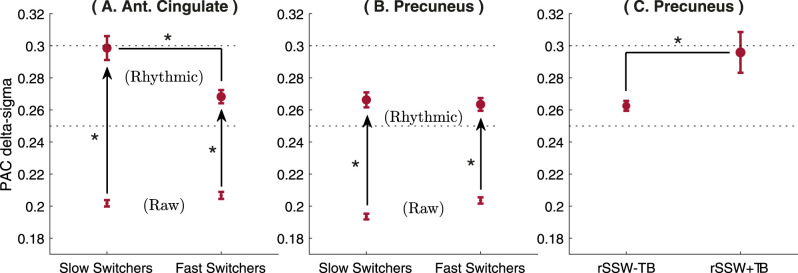
Delta–sigma PAC in the original and rhythmic time series. Average values and standard error of the mean for rPAC (rhythmic) and PAC (raw), for slow and fast switchers during NREM3 sleep in the anterior cingulate (***A***) and in the precuneus (***B***). In the precuneus, rPAC values are shown in ***C*** for all rSSWs without TBs (rSSW − TBs) and for rSSWs codetected with a TBs (rSSW + TBs). For each event type, arrows indicate significant differences between PAC and rPACs values. Stars indicate significance value (*: *p* ≤ 0.01).

As shown in Figure 10, *A* and *B*, rPAC values were significantly higher than PAC values for both types of switchers in the two brain regions (Mann–Whitney *U* tests, *p* < 0.001 for all events). Moreover, rSlowS showed higher rPAC as compared with rFastS in the anterior cingulate during NREM3 (Fig. 10*A*; *U* = 2.82; *p* = 0.005). This difference between switchers was not significant with standard PAC in the anterior cingulate (*U* = 1.85; *p* = 0.06). In the precuneus, no significant differences in rPAC were found between switchers (Fig. 10*B*; *U* = 0.18; *p* > 0.1).

Since induced theta power was observed around the transition of rSlowS and rFastS in the precuneus (Fig. 9*A*2,*C*2), we also explored the impact of TBs events on delta–sigma rPAC during rSSWs in the precuneus. This was prompted by previous literature suggesting that NREM TBs precede slow-wave-spindle complexes and facilitate delta–sigma PAC (Gonzalez et al., 2018). We identified rSSWs co-occurring with TBs (<500 ms; rSSWs + TBs events) and rSSWs that did not (rSSWs − TBs). We hypothesized higher delta–sigma rPAC for rSSWs + TBs events as compared with rSSWs − TB in the precuneus.

As shown in Figure 10*C*, delta–sigma rPAC was higher for rSSWs + TBs compared with that for rSSWs − TBs (*U* = 2.46; *p* = 0.01). Importantly, rSSW + TB events represented ∼6% of all rSSWs in NREM3 (116/1,815 rSSWs). Although rSSWs + TBs are rare, they constitute a significant modulator of rPAC in NREM3.

In summary, delta–sigma PAC was consistently higher in the rhythmic compared with the original time series. Importantly, RnB revealed that delta–sigma PAC is differentially modulated by specific sleep events: rSSWs increased rPAC in the anterior cingulate, whereas TBs enhanced delta–sigma PAC in the precuneus.

## Discussion

The RnB algorithm introduces a novel approach to signal processing in electrophysiology, by disentangling rhythmic activity from the arrhythmic background at the time series level. Spectral methods such as FOOOF and IRASA can separate rhythmic peaks from the scale-free background in the power spectrum (Pani et al., 2022), but they remain limited for time-resolved analyses and signal processing: instantaneous phase, amplitude, and PAC estimates remains biased when the background deviates from a simple power law. To address this limitation, we leveraged the wavelet time–scale paradigm to attenuate scale-free activity at the level of wavelet coefficients, producing rhythmic time series with preserved oscillatory components. Simulations and intracranial NREM sleep recordings confirmed that RnB reliably recovers transient oscillations across multiple frequency bands. Importantly, rhythmic time series extracted by RnB revealed stronger delta–sigma PAC during NREM3 sleep, underscoring its value for studying cross-frequency interactions.

### Dynamic of the scale invariance and NREM sleep EEG spectroscopy

Growing evidence indicates that the scale-free background is neither constant nor unitary: it changes across sleep stages and often exhibits frequency-specific “knees,” where the spectrum bends away from a single power law (Gao, 2016; Gao et al., 2020; Brake et al., 2024). In NREM sleep, these knees overlap with the delta range, “hiding” slow oscillatory peaks (Bódizs et al., 2021; Gerster et al., 2022; Schneider et al., 2022). The wavelet-based RnB framework is particularly robust to this problem for several reasons.

First, wavelet-based estimates provide more reliable characterization of the scale-free regime and of low power than Fourier-based approaches (Abry et al., 2019). This advantage arises from key wavelet properties: (1) their vanishing moments, which cancel slow signal drifts or polynomial trends according to their regularity parameter (a), and (2) their “constant-Q” property, which adapts the time window to each scale and thus maintains a roughly constant number of oscillatory cycles per frequency band. In contrast, Fourier methods use a fixed window size, resulting in poorer resolution and biased low-frequency estimates. Second, *RnB* applies the $1/f^{\beta }$ correction in the timescale domain, attenuating scale-free activity at each scale and time point. This repeated, scale-specific correction enhances robustness by preserving transient low-frequency oscillations in the reconstructed rhythmic signal. As a result, when the rhythmic signal is reanalyzed spectrally, oscillatory peaks remain clearly identifiable—even when overlapping with a scale-free knee—whereas global spectral parameterization may flatten or distort them (Gerster et al., 2022). Finally, by removing the low-frequency approximation term during synthesis of the rhythmic time series (the “residue” in Eq. 4), RnB eliminates stable departures from the arrhythmic baseline without suppressing nonstationary events. Together, these features enable a genuine spectroscopy of sleep rhythms and allow recovery of transient delta oscillations critical for studying their interactions with spindles.

Simulations confirm that RnB preserves delta and sigma amplitudes across a range of 1/*f* exponents (Fig. 5). While RnB provides robust estimates of both oscillatory and arrhythmic activity, our simulations revealed residual cross-component influences. These were modest (in general, *R*^2^ < 0.01) but became more substantial in the delta range: strong delta oscillations increased β estimates, explaining up to ∼30% variance in RnB β estimates. Given the importance of delta activity in sleep research, this coupling must be acknowledged as a potential confound. At the same time, the systematic nature of the effect makes it tractable, suggesting that future refinements (e.g., calibration, iterative separation) could mitigate this bias and enhance the reliability of RnB in high-delta contexts.

### A case study: detection of rhythmic SW and switchers during NREM sleep

Our intracranial data further showed that the rhythmic time series provided by RnB recovered the frontal dominance of rhythmic delta in NREM3, consistent with established slow-wave topography (Steriade, 2006). Using spectroscopy-adapted criteria (Muehlroth and Werkle-Bergner, 2020; Donoghue et al., 2022), RnB enabled detection of SSWs in the rhythmic time series (rSSWs). Importantly, rSSWs in anterior regions modulated sigma power more strongly than those in the precuneus, corroborating results from EEG studies indicating that frontal waves serve as more potent triggers for spindles (Cox et al., 2014) and supporting the validity of our results.

Notably, we further observed lower scaling exponents during rSSWs in the anterior cingulate compared with NREM sleep epochs without rSSWs, which is aligned with earlier spectral parametrization findings (Lendner et al., 2020). This result also converges with studies linking lower scaling exponents to higher neuronal excitability (Gao et al., 2017), which is also observed during the depolarization phase of SSWs (Steriade, 2006). Interestingly, although simulations indicated inflation of β estimates under higher delta amplitudes, RnB remains sensitive to transient reductions in β during NREM3 SSWs and rSlowS, reinforcing the validity of our estimates in relevant physiological ranges.

We also reproduced the fast–slow switchers classification of SSWs in the rhythmic signal (Bouchard et al., 2021). In the anterior cingulate, the transition of slow switchers (rSlowS) was associated with higher phase-locked sigma power, and rSlowS showed higher delta–sigma rPAC when compared with rFastS. These results support the idea that SSWs with prolonged depolarization periods, such as rSlowS, promote stronger spindle generation (Botella-Soler et al., 2012; Cox et al., 2018, 2020; Chylinski et al., 2022). In the precuneus, rSSW–sigma rPAC depended more on co-occurring TBs, consistent with their proposed role as an “early” downstate that favors subsequent spindle emergence (Gonzalez et al., 2018; Jiang et al., 2019). In our data, both event types were relatively rare: most of rSSWs were classified as rFastS (Fig. 8), and TBs occurred in ∼6% of all rSSWs (see Results, The rhythmic time series highlights coupled delta and sigma rhythms in NREM3 sleep). The scarcity of these specific events likely contributes to the high variability of delta–sigma PAC across studies (Cox et al., 2019). Collectively, our findings extend MEG observations suggesting that locally synchronized transient sigma rhythms associated with rSlowS and TBs may act as specific drivers of broader spindle synchronization (Zerouali et al., 2014).

### Enhancing PAC measures with RnB

Finally, delta–sigma PAC was higher for SSWs detected in RnB's rhythmic signal compared with those detected in the raw signal. This enhancement likely reflects that the rhythmic time series isolate genuine oscillatory activity, thereby increasing the specificity of rSSW detection and PAC estimation, albeit at the cost of sensitivity, resulting in a lower number of detected events in rhythmic compared with original time series. Our trade-off thus favors precision over exhaustivity. Our finding aligns with the notion that scale-free signal fluctuations can introduce spurious SSW detections and lower PAC estimates, thereby increasing PAC variability (Aru et al., 2015; Donoghue et al., 2022). Such variability has repeatedly been noted as a source of small and heterogeneous PAC effect sizes in sleep research (Mander et al., 2017; Jurkiewicz et al., 2021). By filtering out these arrhythmic confounds, RnB enhances the isolation of rhythmic events, improving the robustness of both slow-wave detection and PAC measures. This is particularly valuable for studying rhythm-dependent interactions across hippocampo–thalamo–cortical networks, where arrhythmic activity may differ between regions (Staresina et al., 2023).

### Future directions

RnB offers a practical shift in EEG signal processing, offering a wavelet-based framework to extract “true” oscillations from complex $1/f^{\beta }$ activity, even when background vary over time or involve multiple “scale-free” regimes. Here, we adapted detection criteria for known NREM sleep rhythms (rSSWs, TBs) to the rhythmic time series, serving as proof of concept rather than an exhaustive event characterization. The next step is to detect and classify rhythmic events directly in the RnB signal to develop new empirical detection criteria. For instance, matching pursuit approaches (Durka and Blinowska, 1995; Krstulović and Gribonval, 2006; Durka et al., 2024) or cycle-by-cycle analysis (Cole and Voytek, 2019) implemented within the rhythmic signal might identify subtle SSWs, TBs, or sigma bursts that standard detection technique may overlook.

## Conclusion

By leveraging the wavelet-based multiresolution “timescale” paradigm, RnB disentangles NREM sleep rhythms from their arrhythmic background, enabling extraction of rhythms from scale-free dominated signals. Our findings show that this approach preserves physiologically meaningful oscillatory dynamics to study interfrequency interactions of neural rhythms. RnB allows to shift the spotlight: in the raw signal, scale-free dynamics dominate the stage, whereas RnB brings the oscillatory actors to the forefront. In the long run, RnB may support the development of a refined detection and classification system of rhythmic sleep events across the lifespan and in clinical populations, where altered scale-free dynamics may obscure oscillatory markers.

10.1523/ENEURO.0235-25.2025.d1 Data 1 Download Data 1, ZIP file.

## Synthesis

Reviewing Editor: Adrien Peyrache, McGill University Decisions are customarily a result of the Reviewing Editor and the peer reviewers coming together and discussing their recommendations until a consensus is reached. When revisions are invited, a fact-based synthesis statement explaining their decision and outlining what is needed to prepare a revision will be listed below. The following reviewer(s) agreed to reveal their identity: Agustín Solano.

This study introduces a novel method for resolving oscillatory and aperiodic signals in the time domain, with promising applications for improved characterization of brain activity. While the evidence presented is robust, the manuscript would benefit from in-depth editing to improve readability.

The reviewers raised several specific concerns, which need to be addressed to warrant publication:

1/ Jargon and technical terms should be properly introduced and clearly defined.

2/ Clarify how the wavelet-based method differs from other time-resolved signal analysis approaches, such as the Hilbert transform.

3/ Improve the presentation of the method, which is currently scattered across many figures; a more concise and integrated exposition would enhance clarity.

Specific comments:

Overall, the manuscript is extremely difficult to read and very technical for a JNeuro manuscript. Also, it is more technical and less approachable than comparable papers, such as Donoghue et al. 2020 Nature Neuro (FOOOF) or Wen &Liu 2016 Brain Topography (IRASA).

The key advantage of the proposed solution is that oscillations/aperiodic activity can be estimated in the time- and not only frequency-domain (as in FOOOF). Therefore, the authors use a wavelet approach, however, it remains unclear if this is really different from Fourier or Hilbert approaches as these provide highly similar results (see Bruns et al. 2004).

One reason why the paper is not approachable is that many terms are not defined and rely on specialized expert language. For example, on page 7 line 12 the authors discuss resonance and line 13 infrared diverge... while this might be established physical terms (and well suited for a specialized journal), they require a bit more elaboration for a neuroscience audience. This is just one example, but similar wordings can be found on almost every page and thus, render this manuscript difficult to read.

The figures fail to stand on their own and raise additional questions that the legends do not address. For example, in Fig 1, panel E1: Why is the rhythmic factor > 1 and what does this entail? Likewise, in Fig 1 panels D1/D2 - How broad is the distribution around the mean? In Figure 3, it is almost impossible to understand how the signal with a beta of 2.27 can give rise to a distribution as displayed in panels B (peak at 2.5). In Figure 7, the terms persistence and anti-persistence are not well defined and it is puzzling how the authors decided on the two brain regions of interest (ACC and Precuneus). Maybe one motivation was to identify putative cortical origins of SOs and spindles, respectively, but why not include the hippocampus as a source for the ripple oscillation?

While it is great that the authors make the method details available for the community, the approach might be more broadly adopted if the authors make the manuscript and tool approachable. For me, it is unclear why the methods/results are distributed across 11 figures that employ mainly expert terminology and suboptimal visualizations.

A more detailed explanation of how Rhythmic SSWs (rSSW) were detected would be beneficial. While the two-step process is outlined (initial delta low-pass filter + high-frequency activity for polarity, then adaptive band-pass filter), a clearer, step-by-step description for a broader audience would enhance understanding.

The explanation of induced power scalogram and phase-locked scalogram (lines 390-396) is good, but expanding on the detailed procedure for their computation, especially for a wider audience, could be useful. What are the specific steps involved in calculating these?

Regarding the rationale for formula 15 of PAC, while the formula is provided, a brief explanation of its derivation or why this specific form is chosen would be helpful for readers not intimately familiar with this particular PAC formulation.

Figure 8: The horizontal gray bars in Figure 8.D2 for the precuneus, where rFastS and rSlowS did not differ in arrhythmic and rhythmic estimates, are misleading. They should either be removed or clearly re-explained as indicating frequency ranges rather than significant differences. A different visual cue for frequency ranges would be preferable.

Figure 8 Caption (Line 1039): The caption states "precuneus (B1)" which should be "A2" based on the figure layout. This is a minor but important correction for clarity.

## Author Response

Dear Dr Peyrache, We thank you for the opportunity to revise our manuscript and for forwarding both your comments and those of the reviewers. After carefully reviewing the feedback, we were surprised to note that several of the reviewer's remarks refer to figures, sections, or terminology that are not present in the version of the manuscript submitted to your journal. Several points also address elements that were already included in the submitted version. From the nature of the comments, it appears that they may refer to an earlier version of our work submitted to the Journal of Neuroscience, rather than to the version submitted here to eNeuro. The manuscript originally submitted to eNeuro had already been substantially revised relative to that prior submission.

Nonetheless, we have further addressed each comment constructively and in detail, to render the manuscript as accessible as possible. In our response, we provide clarifications and cite the precise locations in the revised manuscript so that the reviewer and the editorial team can easily verify how the concerns have been handled. In the annotated manuscript (submitted "for reviewers"), all new or substantially modified text is highlighted or presented in boldface, in accordance with eNeuro's editorial guidelines.

We trust these revisions will help resolve any confusion and allow the manuscript to be assessed on its current merits.

Reviewer Comments and Author's Responses ------ Comment 1. The key advantage of the proposed solution is that oscillations/aperiodic activity can be estimated in the time- and not only frequency-domain (as in FOOOF). Therefore, the authors use a wavelet approach, however, it remains unclear if this is really different from Fourier or Hilbert approaches as these provide highly similar results (see Bruns et al. 2004).

Response:

We thank the reviewer for this important point. As Bruns (2004) demonstrated, Fourier, Hilbert, and wavelet transforms are formally equivalent when narrowband filters are appropriately matched; differences then mostly arise from parameter choices. However, as highlighted by Abry et al. (2019), fixed-window Fourier and short-time Fourier transforms (STFT) exhibit a systematic bias in estimating both scaling exponents and low-frequency power, especially in the presence of "knees" or deviations from strict power-law scaling. This limitation is particularly relevant during NREM sleep, where such knees overlap with delta oscillations.

Abry et al (2019) showed that wavelet, by contrast, avoids such bias and obtain robust scale-free estimates and power estimates at low-frequency. This advantage stems from the wavelet's constant-Q property, which provides scale-adaptive time-frequency resolution, maintaining optimal frequency precision across the spectrum. Furthermore, beyond formal equivalence, the multiresolution time-scale paradigm used in wavelet analysis allows direct characterization of the signal scaling properties in the time domain.

This leads us to use spline-based wavelets, which possess a fractional differentiation property (Blu &Unser, 2000) that is specifically suited for describing scale-free dynamics (and thus, to whiten it). Leveraging these features, the RnB algorithm denoises wavelet coefficients from the scale-free background across time and scale, thereby reconstructing a rhythmic time series that isolates oscillatory activity with optimal frequency resolution.

Conventional Fourier or Hilbert approaches cannot achieve this level of dynamic adaptability.

To clarify these aspects, we have revised the Methods section (lines 129-157) to introduce the wavelet time-scale framework in a brief didactic section for neuroscience readers, and updated Figure 1 accordingly. The advantages of the wavelet approach over Fourier-based analyses for characterizing rhythmic and aperiodic activity during sleep are also now discussed specifically in the revised Discussion (lines 767-787).

Comment 2: . " Overall, the manuscript is extremely difficult to read and very technical for a JNeuro manuscript. Also, it is more technical and less approachable than comparable papers, such as Donoghue et al. 2020 Nature Neuro (FOOOF) or Wen &Liu 2016 Brain Topography (IRASA). Jargon and technical terms should be properly introduced and clearly defined. Many terms are not defined and rely on specialized expert language. For example, on page 7 line 12 the authors discuss resonance and line 13 infrared diverge... while this might be established physical terms (and well suited for a specialized journal), they require a bit more elaboration for a neuroscience audience. This is just one example, but similar wordings can be found on almost every page and thus, render this manuscript difficult to read. " Response. We appreciate the reviewer's concern about accessibility and agree that clarity for a broad neuroscience readership is essential. This is why the Methods section already began with two introductory subsections ("From spectral to wavelet" and "Wavelet perspective on rhythmic and arrhythmic processes") designed to clarify key wavelet concepts. In the revision, we further refined each section. Specifically, we added a brief didactic presentation of the fundamentals of our method (described above), but also significantly simplified and vulgarized the Methods section describing RnB mathematical underpinnings (section revised 'Mother wavelet', wavelet family and time-scale expansion of a signal' at lines 160-195). We also simplified the step-by-step description of the algorithm, for a more concrete understanding of its operations (RnB Procedure' at lines 196-260). All those sections were carefully revised with the help of neuroscience experts to ensure conceptual clarity and accessibility to a broad readership.

Also, we systematically reviewed the manuscript to replace or clarify specialized terminology which may not be understood by a broad neuroscience audience. We ensured that technical terms are explicitly defined at their first mention. For example, the term resonance-now defined in lines 111-119-is presented as a localized spectral "bump" where oscillatory power is defined as the product between rhythms and the concurrent aperiodic background. This preserves its meaning in dynamical systems while grounding it in a neurophysiological framework. The term "infrared divergence" noted by the reviewer is a misattribution, since it does not appear anywhere in the original nor revised manuscript submitted to your journal. We also clarified our use of the term spectroscopy for clarity purposes (lines 262-271). Part of the results (lines 542-571) were rewritten to increase the clarity of our findings. Finally, specific sections of the discussion were reworked with the explicit intention of better linking our sleep results with simulation findings (788-792; 768-777). We also elaborated more clearly why the rhythmic time series might be more sensitive to delta-sigma PAC during NREM sleep (lines 837-852).

Overall, these changes aim to increase the relevance of the paper for NREM sleep expert and broad neuroscience readership, and to ensure that readers without prior exposure to wavelet-analysis or advanced signal analysis methods can follow the logic and implementation of our approach, while retaining the mathematical rigor and precision required for critical evaluation and reproducibility.

Comment 3 &4. The figures fail to stand on their own and raise additional questions that the legends do not address. For example, in Fig 1, panel E1: Why is the rhythmic factor > 1 and what does this entail? Likewise, in Fig 1 panels D1/D2 - How broad is the distribution around the mean? In Figure 3, it is almost impossible to understand how the signal with a beta of 2.27 can give rise to a distribution as displayed in panels B (peak at 2.5).

Response. We appreciate the reviewer's feedback, but we must point out that several of the comments refer to figures that were not included in the version of the manuscript submitted to this journal. Figure 1 had no panels labelled E1 or D1/D2, and Figure 3 had no parameter "β = 2.27". These comments appear to refer to a figure set from a version previously submitted to another journal.

For clarity, Figure 1 from the previous J.Neuroscience submission was not part of the current manuscript under review at eNeuro. Furthermore, what the reviewer refers to as "Figure 3" appears to correspond to the Figure 2 in the original eNeuro submission. In that figure, the "β = 2.27" value referred to the scaling exponent of a single displayed NREM rhythmic timeseries. In the revised manuscript, this singular rhythmic signal has been moved to Figure 1G, which is now in direct correspondence with the raw signal from the same epoch on Figure 1A.

We have carefully revised all figure legends to ensure that each figure is fully interpretable on its own. As Figure 1 was not addressed in the reviewer's comments, we now highlight its role in-text more explicitly in the revised version throughout the method. Acronyms and key terms are now defined repeatedly in the legends, independent of the main text.

Comment 5. In Figure 7, the terms persistence and anti-persistence are not well defined and it is puzzling how the authors decided on the two brain regions of interest (ACC and Precuneus). Maybe one motivation was to identify putative cortical origins of SOs and spindles, respectively, but why not include the hippocampus as a source for the ripple oscillation? Response: The terms 'persistence' and 'anti-persistence' have been removed from all figures and text for clarity, as they were not essential to our argument.

The choice of anterior cingulate and precuneus is justified in Methods (NREM sleep analyses using RnB', lines 366-367). These cortical regions were indeed chosen based on prior literature linking them to slow oscillations and spindles.

The hippocampus was not included due to the 200 Hz sampling rate, which is insufficient to resolve ripple (>100 Hz) activity in this dataset.

6.While it is great that the authors make the method details available for the community, the approach might be more broadly adopted if the authors make the manuscript and tool approachable. For me, it is unclear why the methods/results are distributed across 11 figures that employ mainly expert terminology and suboptimal visualizations.

Response: We hope the modifications discussed above have already increased the clarity of our paper. However, we note that the manuscript contained 10 figures (not 11 as stated). Moreover, each of these have a specific contribution related to the validation and application of our method to NREM sleep recordings. Figures 1-2 illustrate the core principle of our approach; Figures 3-5 present simulation results that validate the method (ensuring that its output is valid compared to simulation parameters); and Figures 6-10 demonstrate novel NREM sleep findings, each highlighting a distinct aspect (i.e. Figure 6 : rhythmic activity in each NREM sleep stage, Figure 7 : rhythmic event detection, Figure 8 :the spectroscopy of rhythmic slow waves; Figure 9 : time-frequency analyses of rhythmic slow waves; Figure 10: the benefit of applying the rhythmic timeseries to estimate phase-amplitude coupling [PAC]).

We have thus revised the text from the Results and Figures 6-7-8-9 Titles and Legends to make the contribution of each more explicit.

7. A more detailed explanation of how Rhythmic SSWs (rSSW) were detected would be beneficial. While the two-step process is outlined (initial delta low-pass filter + high-frequency activity for polarity, then adaptive band-pass filter), a clearer, step-by-step description for a broader audience would enhance understanding.

Response: We agree and have expanded the Methods to provide a step-by-step description of rSSW detection in the rhythmic time series (lines 371-409). In intracranial bipolar recordings, the polarity of slow waves is non-trivial; we now explicitly state that we determine the down-state polarity using the reduction in gamma-band power (30-80 Hz) across half-waves, following von Ellenrieder et al. (2016). We also clarified that the second pass uses a region- and stage-specific delta band derived from our RnB spectroscopy. These clarifications are now provided as numbered steps in "Detection of rhythmic slow waves (rSSWs)" with all filter bands and criteria reported (lines 392-420). Finally, we added clarifications regarding the computation of the transition frequency (lines 428-434).

8. The explanation of induced power scalogram and phase-locked scalogram (lines 390-396) is good, but expanding on the detailed procedure for their computation, especially for a wider audience, could be useful. What are the specific steps involved in calculating these? Response: Thank you for this suggestion. Computation steps are now clearly explained in Methods (Time-frequency analyses of rSSWs', lines 453-461).

9. Regarding the rationale for formula 15 of PAC, while the formula is provided, a brief explanation of its derivation or why this specific form is chosen would be helpful for readers not intimately familiar with this particular PAC formulation.

Response: We have chosen the unbiased PAC formulation introduced by Cox et al. (2020). This formulation computes the correlation between instantaneous amplitudes and the complex phase term after subtracting their respective means. This removes bias from differences in mean amplitude or phase distribution, which ensures that PAC values are on the same scale across conditions and subjects, and makes them suitable for direct statistical comparison. We have now expanded the Methods section ("Phase-Amplitude Coupling analyses of rSSWs", lines 482-491) to explain the rationale for using it specifically. We also believe this addition will help readers less familiar with this variant of PAC to understand why it was selected for the present analyses.

10 . Figure 8: The horizontal gray bars in Figure 8.D2 for the precuneus, where rFastS and rSlowS did not differ in arrhythmic and rhythmic estimates, are misleading. They should either be removed or clearly re-explained as indicating frequency ranges rather than significant differences. A different visual cue for frequency ranges would be preferable..

Response: These bars indicate the frequency ranges of interest that were used for statistical analyses of rhythmic fast and slow switchers. We recognize that their previous appearance in Figure 8.D2 could be misinterpreted as significance markers. We have corrected the legend (lines 953-971; 982-986) to clearly state that these are frequency bands, and we have updated the figure to label each band. This should eliminate ambiguity while retaining the information needed for interpreting the upcoming PAC analyses.

Figure 8 Caption (Line 1039): The caption states "precuneus (B1)" which should be "A2" based on the figure layout. This is a minor but important correction for clarity.

Response: The figure caption error (precuneus (B1)' should be 'A2') is corrected.

Conclusion We thank you again for the opportunity to improve our manuscript. We hope these revisions address all concerns, and we remain available for any further suggestions.

Sincerely, Jonathan Dubé &Jean Marc Lina
