# Supplementary material for: Rhythms and Background (RnB): The Spectroscopy of Sleep Recordings
Source: eNeuro. 2026 Feb 3;13(2):ENEURO.0235-25.2025. doi: 10.1523/ENEURO.0235-25.2025 (PMC12867552; doi:10.1523/ENEURO.0235-25.2025)
Supplement: Data 1 — Download Data 1, ZIP file. [file eneuro-13-ENEURO.0235-25.2025-s002.zip › RnB-Wavelet-main/wRnBmain/rnb_wavelet/rnb/FracSpline/references/blu0001.pdf]

THE FRACTIONAL SPLINE WAVELET TRANSFORM: DEFINITION AND IMPLEMENTATION

Thierry Blu and Michael Unser

Biomedical Imaging Group
Swiss Federal Institute of Technology EPFL
CH-1015 Lausanne EPFL, SWITZERLAND

ABSTRACT

We define a new wavelet transform that is based on a recently defined family of scaling functions: the fractional B-splines. The interest of this family is that they interpolate between the integer degrees of polynomial B-splines and that they allow a fractional order of approximation.

The orthogonal fractional spline wavelets essentially behave as a fractional differentiators. This property seems promising for the analysis of $1/f^\alpha$ noise that can be whitened by an appropriate choice of the degree of the spline transform.

We present a practical FFT-based algorithm for the implementation of these fractional wavelet transforms, and give some examples of processing.

1. INTRODUCTION

Wavelets are a powerful tool for for signal compression and analysis. They are especially useful for detecting and characterizing signal singularities. This occurs because a wavelet with n vanishing moments behaves like a differentiator of order $n+1$: $\hat{\psi}(\omega) \propto \omega^{n+1}$ as $\omega \to 0$.

However, in some applications where fractal signals or fractional Brownian motions (fBm) are involved, it is better that the wavelet behave like a *fractional* differentiator $\hat{\psi}(\omega) \propto \omega^{\alpha+1}$ as $\omega \to 0$, for some noninteger number α.

We have recently defined for all the values α larger than $-\frac{1}{2}$, a set of wavelets indexed by α that have this property [1]. They derive from a scaling function, $\beta^\alpha(x)$ which we named "fractional B-splines" because they interpolate the better-known polynomial B-splines at the integers [2]. These functions have most of the good properties of integer degree B-splines (approximation, regularity, scaling or inductive properties) except for their support which is infinite.

In this paper we will define the orthonormal fractional spline wavelet filters and develop a practical algorithm for the implementation of the associated wavelet transform. This method which uses the FFT algorithm, is *exact* despite the infinite support of the wavelets (no truncation of basis functions, exact treatment of boundaries, perfect reconstruction) and is competitive with Mallat's fast wavelet algorithm for this type of filters [3].

2. FRACTIONAL SPLINES

2.1. Causal and symmetric fractional B-splines

These new functions are localized versions of the one-sided power functions $(x-k)_+^\alpha \stackrel{\text{def}}{=} \max(x-k,0)^\alpha$ [2]

$$\begin{aligned}\beta_+^\alpha(x) &\stackrel{\text{def}}{=} \frac{\Delta_+^{\alpha+1} x_+^\alpha}{\Gamma(\alpha+1)} \\ &= \sum_{k\geq 0} \frac{(-1)^k \binom{\alpha+1}{k}}{\Gamma(\alpha+1)} (x-k)_+^\alpha .\end{aligned} \tag{1}$$

where $\alpha > -1/2$ in order to ensure square integrability. These functions interpolate the usual polynomial B-splines; these are recovered for α integer. They are "causal" in the sense that their support belongs to $\mathbb{R}_+$.

Since the fractional B-splines are not symmetric in general (except for integer degrees), we have also defined symmetrized versions of them. If α is not an even integer, then

$$\begin{aligned}\beta_*^\alpha(x) &\stackrel{\text{def}}{=} -\frac{\Delta_*^{\alpha+1} x_*^\alpha}{2\sin(\frac{\pi}{2}\alpha)\Gamma(\alpha+1)} \\ &= \sum_{k\in\mathbb{Z}} \frac{(-1)^{k+1} \left|\binom{\alpha+1}{k}\right|}{2\sin(\frac{\pi}{2}\alpha)\Gamma(\alpha+1)} |x-k|^\alpha\end{aligned} \tag{2}$$

else, if $\alpha = 2n$ (even)

$$\begin{aligned}\beta_*^{2n}(x) &\stackrel{\text{def}}{=} \frac{(-1)^{n+1}}{\pi\Gamma(2n+1)} \Delta_*^{2n+1} x_*^{2n} \\ &= \frac{(-1)^n}{2n!\,\pi} \sum_{k\in\mathbb{Z}} (-1)^{k+1} \begin{vmatrix} 2n+1 \\ k \end{vmatrix} |x-k|^{2n} \log|x-k|\end{aligned} \tag{3}$$

where the number $\left|{x \atop k}\right|$ is defined as the symmetrized version of the binomial function by $\left|{x \atop k}\right| = \binom{x}{k+\frac{x}{2}}$. Here, we assume that the factorial has been extended to noninteger x by $x! = \Gamma(x+1)$ using Euler's gamma function.

2.2. Properties

We showed in [2] that fractional B-splines decrease like $|x|^{-\alpha-2}$ when $|x| \to \infty$ and that they satisfy a two-scale difference equation governed by a scaling filter H^α whose frequency response is given by

$$\begin{aligned} H_+^\alpha(e^{j\omega}) &= \sqrt{2}\left(\frac{1+e^{-j\omega}}{2}\right)^{\alpha+1} \\ H_*^\alpha(e^{j\omega}) &= \sqrt{2}\left|\frac{1+e^{-j\omega}}{2}\right|^{\alpha+1} \end{aligned} \quad (4)$$

for the causal and the symmetric fractional B-splines of degree α, respectively. This also means that the Fourier transform of the corresponding B-splines are $\left(\frac{1-e^{-j\omega}}{j\omega}\right)^{\alpha+1}$ and $\left|\frac{1-e^{-j\omega}}{j\omega}\right|^{\alpha+1}$, respectively.

One of the most interesting properties of these functions is that they have a fractional order of approximation $\alpha+1$. In particular, this implies that any polynomials of degree $\leq \lceil\alpha\rceil$ can be expressed as a linear combination of $\beta^\alpha(x-k)$, $k \in \mathbb{Z}$. In this respect, they behave like approximators that have a higher, integer, approximation order $1+\lceil\alpha\rceil$. This property is useful since it ensures that the very lowpass information—essential in most types of image processing—is exactly represented by the lowpass branch of the filterbank that will be built from the fractional B-spline.

A plot of the B-splines of degree $\alpha = 1/2$ is given in Fig. 1.

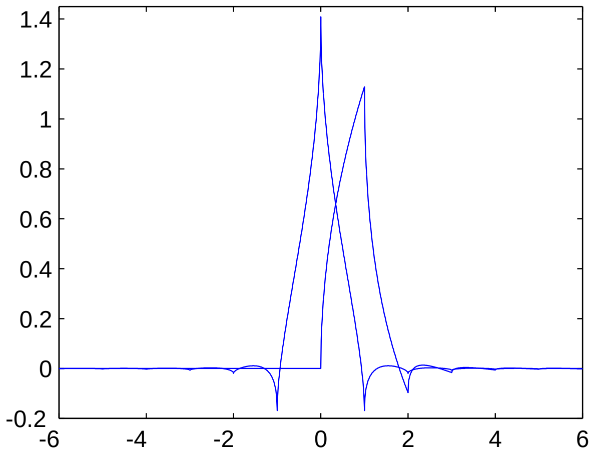

Figure 1: plot of $\beta_+^{\frac{1}{2}}(x)$ and $\beta_*^{\frac{1}{2}}(x)$

From now on, we will drop the subscripts (nonsymmetric "+" or symmetric index "*") since all the expressions we will give will be valid independently of the subscript.

2.3. Orthogonal Fractional Splines

Using a standard technique [4], it is possible to orthonormalize the fractional B-splines. The obtained functions now satify a two-scale relation with the following orthonormal filters

$$H_\perp^\alpha(e^{j\omega}) = H^\alpha(e^{j\omega})\sqrt{\frac{A^{2\alpha+1}(e^{j\omega})}{A^{2\alpha+1}(e^{2j\omega})}} \quad (5)$$

where $A^\alpha(z)$ is the autocorrelation filter of a B-spline of degree α, i.e.

$$A^\alpha(e^{j\omega}) = \sum_{n\in\mathbb{Z}} e^{-jn\omega} \int \beta_+^\alpha(x)\beta_+^\alpha(x+n)\,dx. \quad (6)$$

2.4. Fractional Splines Wavelets

The wavelets are constructed by linear combination of scaling functions. In the orthogonal case, the frequency response of the generating filter is given by

$$G_\perp^\alpha(e^{j\omega}) = e^{-j\omega} H_\perp^\alpha(-e^{-j\omega}) \quad (7)$$

according to classical wavelet theory [5]. It is interesting to note that the fractional spline wavelets behave as a fractional differentiator of order $\alpha+1$ for low frequencies: this is because, for ω close to 0, the nonsymmetric and the symmetric fractional spline wavelets are $\propto (-j\omega)^{\alpha+1}$ and $\propto |\omega|^{\alpha+1}$ respectively to the first order in ω. This property can also be very useful when dealing with $1/f^\beta$ signals, since the fractional differentiator of order β "whiten" them.

3. ORTHOGONAL FRACTIONAL SPLINE WAVELET TRANSFORM

Now that we have defined the orthonormal lowpass and highpass filters, we want to implement the corresponding analysis and synthesis filterbanks, as shown in Fig. 2.

The difficulty with these filters is that they are infinitely supported and that, at least for small values of α, the efficient number of coefficients to keep may be quite large, sometimes even larger than the size of the signal. In such a situation, it is quite advantageous to use a Fourier implementation which will ensure perfect reconstruction to a very high accuracy.

We will assume that the input signal is given by its coefficients $\{x_k\}_{k=0\ldots N-1}$. We extend this signal

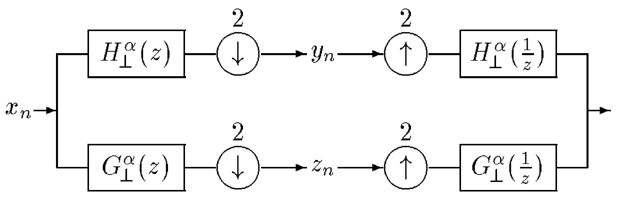

Figure 2: Analysis/Synthesis filterbank with fractional spline filters

to any value of the index $k \in \mathbb{Z}$ by periodization, i.e., $x_{N+k} \stackrel{\text{def}}{=} x_k$.

3.1. Analysis

The filtering and downsampling operation on the extended signal x_n provide the outputs y_n and z_n as illustrated in Fig. 2. In order to be able to apply the same filterbank to these outputs (or at least to the low-pass) we need these outputs to be periodic as well. This can happen only if N is even; this implies that y_n, z_n are $\frac{N}{2}$-periodic. Thus, by induction, in order to propagate the periodicity condition to all possible decomposition levels, N has to be a power of 2.

Let us denote

$$\begin{aligned} X_k &= \textstyle\sum_n x_n e^{-j2\pi\frac{nk}{N}} \text{ for } k = 0 \ldots N-1 \\ Y_k &= \textstyle\sum_n y_n e^{-j2\pi\frac{nk}{N/2}} \text{ for } k = 0 \ldots \frac{N}{2}-1 \\ Z_k &= \textstyle\sum_n z_n e^{-j2\pi\frac{nk}{N/2}} \text{ for } k = 0 \ldots \frac{N}{2}-1 \end{aligned}$$

the DFTs of x_n, y_n and z_n, respectively. Let us also denote by $H_{\perp,k}^\alpha$ and $G_{\perp,k}^\alpha$ the values taken by $H_\perp^\alpha(e^{j2\pi\nu})$ and $G_\perp^\alpha(e^{j2\pi\nu})$ at the frequency points $\nu = \frac{k}{N}$.

Then, for $k = 0 \ldots \frac{N}{2} - 1$, we have

$$\begin{aligned} Y_k &= \frac{1}{2}\left[H_{\perp,k}^\alpha X_k + H_{\perp,k+\frac{N}{2}}^\alpha X_{k+\frac{N}{2}}\right] \\ Z_k &= \frac{1}{2}\left[G_{\perp,k}^\alpha X_k + G_{\perp,k+\frac{N}{2}}^\alpha X_{k+\frac{N}{2}}\right] \end{aligned} \tag{8}$$

Of course the number operations needed in (8) are limited only to $k = 0 \ldots \frac{N}{4} - 1$ since y_n and z_n are real sequences, which implies conjugate symmetry of the DFTs.

The cost of this implementation amounts to one FFT of x_n, then four complex multiplications and two complex additions over $\frac{N}{4}$ frequency points. If we count that a real addition and a real multiplication are both worth one operation, and that a complex multiplication amounts to six operations, while a complex addition is worth 2 of them, then the cost of (8) is $7N$. For a full wavelet decomposition we would thus have $14(N-1)$ operations, due to (8) only.

The cost of the inverse Fourier transforms of the outputs and of the input x_n is to be added to this number. For a full wavelet decomposition, this amounts to $2CN\log_2 N + (2C+14)(N-1)$, where $C \approx 3$ is a constant that evaluates the efficiency of the FFT algorithm.

Typically the cost of the Fourier transformations is larger than (8). For example, when $N = 256$ we find that the number of operations amounts to $68N$. This number has to be compared to $4L$, which is the number of operations needed to iterate an orthogonal filterbank whose filters are of length L: for $N = 256$, the Fourier algorithm is more efficient when $L > 17$. In general, a direct implementation of the fractional spline filterbank require filters that are well longer than 2 in order to ensure a decent reconstruction error: our algorithm is thus more efficient.

3.2. Synthesis

The reconstruction of x_n from y_n and z_n follows easily from (8) (and also from classical filterbank theory [6]), since the filters are orthonormal

$$\begin{aligned} X_k &= \overline{H_{\perp,k}^\alpha} Y_k + \overline{G_{\perp,k}^\alpha} Z_k \\ X_{k+\frac{N}{2}} &= \overline{H_{\perp,k+\frac{N}{2}}^\alpha} Y_k + \overline{G_{\perp,k+\frac{N}{2}}^\alpha} Z_k \end{aligned} \tag{9}$$

Obviously, each synthesis iteration costs the same as an analysis iteration.

3.3. Practical computations

For the computation of the Fourier transforms of the fractional spline filters we need to evaluate the autocorrelation filter at $\nu = \frac{k}{N}$ for $k = 0 \ldots N-1$. For this, we use the Poisson-equivalent expression of (6), i.e., $\sum_{n\in\mathbb{Z}} |\hat{\beta}_+^\alpha(\omega + 2n\pi)|^2$. More precisely, since we must limit this summation to a finite number of terms, we evaluate A^α using the following asymptotic equivalent

$$\begin{aligned} A^\alpha(e^{j2\pi\nu}) \approx &\sum_{|n|\le N} |\hat{\beta}_+^\alpha(2\pi(\nu+n))|^2 + \\ &\left|\frac{\sin(\pi\nu)}{\pi}\right|^{2\alpha+2} \left[\frac{\frac{2}{2\alpha+1}}{N^{2\alpha+1}} - \frac{1}{N^{2\alpha+2}} + \right. \\ &\left. \frac{(\alpha+1)(\frac{1}{3}+2\nu^2)}{N^{2\alpha+3}} - \frac{(\alpha+1)(2\alpha+3)\nu^2}{N^{2\alpha+4}}\right] \end{aligned} \tag{10}$$

An accurate analysis of this expression shows indeed that the remainder of the difference between the lhs and the rhs is $O(\frac{1}{N^{2\alpha+5}})$, which ensures an accuracy that is larger than 200 dB with $N = 100$ computed terms, and for all values of $\alpha > -\frac{1}{2}$.

Once the orthonormal fractional spline filters have been evaluated at the frequency points $\nu = \frac{k}{N}$, we need

not recompute the filters at each new iteration. In fact, at the first iteration, we need the values $H^{\alpha}_{\perp,k}$ and $G^{\alpha}_{\perp,k}$ for $k = 0 \ldots N-1$. Then, at the second iteration, we need the values $H^{\alpha}_{\perp,2k}$ and $G^{\alpha}_{\perp,2k}$ for $k = 0 \ldots \frac{N}{2} - 1$, and so on for higher iteration steps. This means that the filters can be precomputed once and for all, before applying the wavelet transform to different signals.

4. EXAMPLES

Our fractional spline iterated filterbank can be used as an analysis tool, in a compression application or for signal synthesis. We give below two examples of a synthesis of an fBm-like noise. As input, we used white noise in the wavelet channels. The same noise was weighted by $2^{j(\alpha+1)}$ in the jth band for each values of α. This ensures that we get a—nonstationary—signal that has, on the average, a spectrum that behaves like $1/f^{\alpha+1}$. We used the nonsymmetric orthonormal filters for the synthesis of the $N = 4096$ samples.

As can be observed in Figs. 3 and 4, the $1/f^{1.4}$ noise seems to be a smoothed version of the $1/f^{1.1}$ noise. This is obviously a consequence of the localization of the fractional spline wavelet transform, a property that is not shared by the DFT. Due to this local property, we expect that our transform can be beneficial either for the synthesis of localized fBm's or for their analysis.

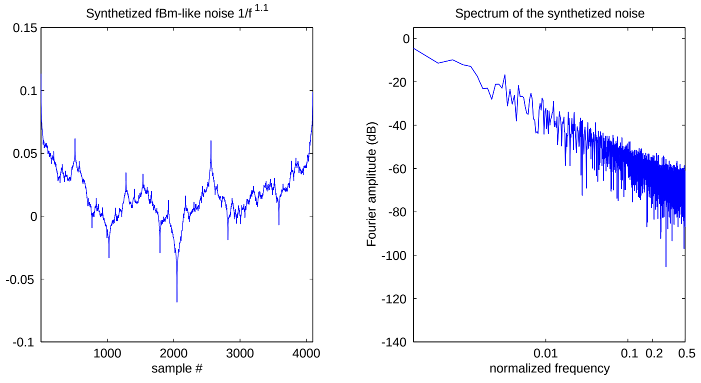

Figure 3: Synthesis of a $1/f^{1.1}$ noise

5. CONCLUSION

We have presented a new wavelet transform based on functions—the fractional splines—that we have recently defined [2]. We have also shown how to implement our wavelet transform efficiently and accurately, using a Fourier technique. We believe that this transform can be especially useful, either for the synthesis of fBm noises, or for the analysis of such signals.

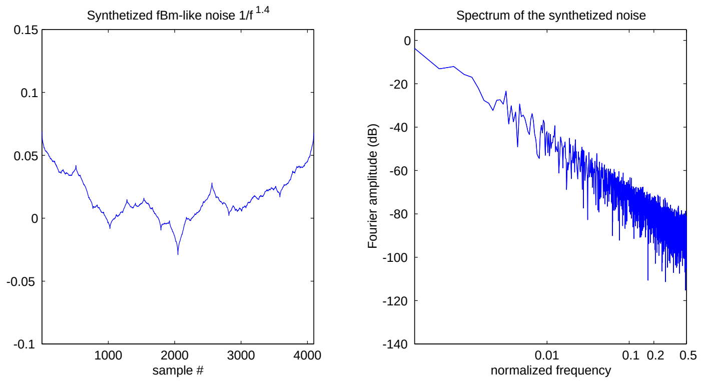

Figure 4: Synthesis of a $1/f^{1.4}$ noise using the same random coefficients as in Fig. 3

The main advantage of such a wavelet technique over the more traditional Fourier method is that it offers the possibility of a local analysis or synthesis.

Matlab M-files implementing a fractional spline wavelet transform (analysis and synthesis) are freely downloadable at

`http://bigwww.epfl.ch/demo/fractsplines/index.html`

with demos and papers on fractional splines.

6. REFERENCES

[1] M. Unser and T. Blu, "Construction of fractional spline wavelet bases," in *Proc. SPIE "Wavelets Applications in Signal and Image Processing VII"*, Denver, CO, 1999, vol. 3813.

[2] M. Unser and T. Blu, "Fractional splines and wavelets," *SIAM Review*, 1999, To appear.

[3] S. Mallat, "A theory for multiresolution signal decomposition: The wavelet decomposition," *IEEE Trans. Pattern Anal. Mach. Intell.*, vol. 11, no. 7, pp. 674–693, July 1989.

[4] Y. Meyer, "Orthonormal wavelets," in *Wavelets, Time-Frequency Methods and Phase Space*, J. M. Combes, Ed., pp. 21–37. Springer, 1989.

[5] S. Mallat, *A Wavelet Tour of Signal Processing*, Academic Press, San Diego, CA, 1998.

[6] G. Strang and T.Q. Nguyen, *Wavelets and Filter Banks*, Wellesley-Cambridge Press, Cambridge MA, 1996.
