# Supplementary material for: Rhythms and Background (RnB): The Spectroscopy of Sleep Recordings
Source: eNeuro. 2026 Feb 3;13(2):ENEURO.0235-25.2025. doi: 10.1523/ENEURO.0235-25.2025 (PMC12867552; doi:10.1523/ENEURO.0235-25.2025)
Supplement: Data 1 — Download Data 1, ZIP file. [file eneuro-13-ENEURO.0235-25.2025-s002.zip › RnB-Wavelet-main/wRnBmain/rnb_wavelet/rnb/FracSpline/references/blu0301.pdf]

A COMPLETE FAMILY OF SCALING FUNCTIONS: THE (α, τ)-FRACTIONAL SPLINES

Thierry Blu and Michael Unser

Biomedical Imaging Group
Swiss Federal Institute of Technology Lausanne (EPFL), Switzerland
e-mail:thierry.blu@epfl.ch,michael.unser@epfl.ch

ABSTRACT

We describe a new family of scaling functions, the (α, τ)-*fractional splines*, which generate valid multiresolution analyses. These functions are characterized by two real parameters: α, which controls the width of the scaling functions; and τ, which specifies their position with respect to the grid (shift parameter). This new family is complete in the sense that it is closed under convolutions and correlations.

We give the explicit time and Fourier domain expressions of these fractional splines.

We prove that the family is closed under generalized fractional differentiations, and, in particular, under the Hilbert transformation. We also show that the associated wavelets are able to whiten $1/f^\lambda$-type noise, by an adequate tuning of the spline parameters.

A fast (and exact) FFT-based implementation of the fractional spline wavelet transform is already available. We show that fractional integration operators can be expressed as the composition of an analysis and a synthesis iterated filterbank.

1. INTRODUCTION

The first instances of fractional splines have been introduced by the authors in [1]. We worked out a fast FFT-based implementation of the fractional spline wavelet transform [2] and put the software on our web server. These functions were extensions of the traditional B-splines to noninteger exponents and were depending on one parameter only—the degree α.

However, this family was not complete under convolutions and correlations—an essential property for considering nonorthogonal projections, or fractional differentiation. This is why we were motivated to generalize the fractional splines by introducing a new parameter, τ, that we interpret as a *shift* of the basis function, while the degree controls their essential *support*.

In this paper, we describe this new family of scaling functions and their associated wavelets. Basically, the scaling functions have a Gaussian-like shape, the size and location of which are given by the first and the second parameter, α and τ, respectively. This extension makes the new family complete; i.e., stable under convolutions and correlations.

We give the explicit time and Fourier domain expressions of these fractional splines. Although these functions do not have compact support, they still decay fast enough for us to consider that they have an effective bounded support.

We show that the generalized fractional derivative of a fractional spline is still a fractional spline with different parameters. In particular, the Hilbert transform of an (α, τ)-fractional spline is an $(\alpha, \tau + 1/2)$-fractional spline.

We also indicate that the associated wavelets behave like fractional derivatives of order $\alpha+1$. This implies that a fractional spline wavelet transform has the property of whitening $1/f^{\frac{\alpha+1}{2}}$-type noise. In practice, this means that by adequate tuning of the degree parameter, it is possible to decorrelate these types of frequently encountered noises. Another potential application of fractional splines is the generation of fractional Brownian motion, or $1/f^\lambda$-type noise. As has been shown by Flandrin et al. [3], the inverse wavelet transform can be used to generate pseudo-fBm by a suitable scaling of the input coefficients. In fact, a true fBm can be generated from the fractional integration of white noise, which makes the fractional spline transform a perfect tool for analyzing or synthetizing such signals [4].

2. DEFINITION

The generalized fractional B-splines are defined by the Fourier expression

$$\hat{\beta}^\alpha_\tau(\omega) = \left(\frac{e^{j\omega}-1}{j\omega}\right)^{\frac{\alpha+1}{2}-\tau} \left(\frac{1-e^{-j\omega}}{j\omega}\right)^{\frac{\alpha+1}{2}+\tau} \quad (1)$$

where $\alpha > -1$ and τ are some real parameters. We call α the "degree" of the spline and τ its "shift" for reasons that will become clear later. Also note that $|\hat{\beta}^\alpha_\tau| = \hat{\beta}^\alpha_0$; i.e., τ has an influence on the phase of the Fourier transform only.

When α is a positive integer and $\tau = (\alpha+1)/2$, expression (1) is the well-known Fourier transform of the non-centered standard B-splines [5, 6]. When $\alpha > -1$ is an real number and $\tau = (\alpha+1)/2$, (1) becomes the fractional extension to the integer B-splines that we have proposed in [1]. A fractional spline is thus a function that can be expressed as a sum of shifted versions of a fractional B(asic)-spline.

In the following, we shall use the standard extension of the factorial, $\alpha! = \Gamma(\alpha+1)$ using Euler's Gamma function which is defined by $\Gamma(p) = \int_0^\infty t^{p-1}e^{-t}\,\mathrm{d}t$ for $p > 0$, and by analytic continuation otherwise. The generalized binomial coefficients are still obtained by the expression $\binom{p}{q} = \frac{p!}{(p-q)!q!}$ and they satisfy the reflection formula $\binom{p}{q} = \binom{p}{p-q}$. For symmetry reasons, we thus also define the centered binomial coefficients by $\left|{p \atop q}\right| = \binom{p}{q+p/2}$. This ensures $\left|{p \atop q}\right| = \left|{p \atop -q}\right|$.

Proposition 1 *The time-domain formulæ for the generalized fractional B-spline β_τ^α is*

$$\beta_\tau^\alpha(t) = \sum_k (-1)^k \left|{\alpha+1 \atop k-\tau}\right| \rho_\tau^\alpha(t-k) \qquad (2)$$

where the function ρ_τ^α is given by

$$\rho_\tau^\alpha(t) = \begin{cases} C_\tau^\alpha |t|^\alpha + D_\tau^\alpha |t|^\alpha \log|t| & \textit{if } \alpha \textit{ is odd} \\ C_\tau^\alpha |t|^\alpha \log|t| + D_\tau^\alpha |t|^\alpha \operatorname{sign} t & \textit{if } \alpha \textit{ is even} \\ C_\tau^\alpha |t|^\alpha + D_\tau^\alpha |t|^\alpha \operatorname{sign} t & \textit{otherwise.} \end{cases} \qquad (3)$$

The constants C_τ^α and D_τ^α are shown in Table 1. It is now easier to understand why α is the degree of the fractional B-spline.

Table 1. Expression of the constants in Proposition 1.

	general case	special cases
C_τ^α	$-\frac{\cos \pi\tau}{2\,\alpha!\,\sin\frac{\pi}{2}\alpha}$	$\frac{(-1)^{\frac{\alpha}{2}+1}\cos\pi\tau}{\pi\,\alpha!}$ for even α
D_τ^α	$-\frac{\sin \pi\tau}{2\,\alpha!\,\cos\frac{\pi}{2}\alpha}$	$\frac{(-1)^{\frac{\alpha+1}{2}}\sin\pi\tau}{\pi\,\alpha!}$ for odd α

Using the same technique as in [1], we can prove that $\beta_\tau^\alpha(t) \propto |t|^{-\alpha-2}$ when $|t| \to \infty$. This ensures that these functions are localized in time. Also note that for negative values of α, $\beta_\tau^\alpha(t)$ assumes infinite values at the integers. These singularities, though, are still integrable.

The fractional B-splines of degree 1 are shown in Fig. 1 for several values of the shift parameter; according to Proposition 1, these functions are linear combinations of shifts of $|t|$ and $|t|\log|t|$. One can already notice that the main effect of τ is to shift the basis function without modifying its shape significantly. Moreover, as α increases, the shape will tend to get more and more preserved, as exemplified in Fig. 2. In fact, similar to [10], we can show that

$$\beta_\tau^\alpha(t) \underset{\alpha\to\infty}{\approx} \sqrt{\frac{6}{\pi(\alpha+1)}}\, e^{-\frac{6}{\alpha+1}(t-\tau)^2},$$

which emphasizes the "shift" and "support" interpretations of the parameters τ and α.

3. PROPERTIES

A key property is that the fractional B-splines satisfy a two-scale difference equation. This is easily seen on the Fourier transform (1) of the B-spline. The z-transform of the resulting scaling filter is

$$H_\tau^\alpha(z) = 2^{-\alpha}(1+z)^{\frac{\alpha+1}{2}-\tau}(1+z^{-1})^{\frac{\alpha+1}{2}+\tau}. \qquad (4)$$

By computing its (binomial) impulse response, we derive the two-scale relation

$$\beta_\tau^\alpha(t) = 2^{-\alpha}\sum_k \left|{\alpha+1 \atop k-\tau}\right| \beta_\tau^\alpha(2t-k). \qquad (5)$$

It is interesting to remark that, although a shifted scaling function does not usually preserve its scaling property, a shifted fractional B-spline $\beta_\tau^\alpha(t-t_0)$ is still very close to a true scaling function, namely $\beta_{\tau+t_0}^\alpha(t)$.

As is apparent from (4), the scaling filter has a zero of multiplicity at least $\lfloor\alpha+1\rfloor$ at $z=-1$. However, in contrast with the classical theory [7], the fractional B-splines not only do reconstruct the polynomials of degree $\lfloor\alpha\rfloor$, but also those of degree $\lceil\alpha\rceil$. Obviously, this unexpected bonus when α is not integer is made possible by the infinite support of the filter—we are in a situation where the theorems of the classical theory do not apply. The fractional B-splines also satisfy the usual stability requirement known as Riesz-basis condition [7]. As a result of the scaling relation (5), the partition of unity and of this stability, we can build a multiresolution analysis in the sense of Mallat [8].

Let us now consider derivatives. In the Fourier domain, N^{th} order differentiation amounts to multiplying by $(j\omega)^N$. Making N non-integer provides a Fourier equivalent of Liouville's definition [9] of fractional derivative. We propose here to generalize even further and define

$$\partial_\tau^\alpha f(t) = \int_{-\infty}^{\infty} (-j\omega)^{\frac{\alpha}{2}-\tau}(j\omega)^{\frac{\alpha}{2}+\tau}\hat{f}(\omega)\frac{\mathrm{d}\omega}{2\pi}. \qquad (6)$$

When $\tau = \frac{\alpha}{2}$, we recover Liouville's fractional derivative. More exciting is the fact that the Hilbert transform $\mathscr{H}$ (which has $-j\operatorname{sign}\omega$ for frequency response) can be expressed as a fractional derivative as well. Specifically, we have

$$\mathscr{H}f = -\partial_{1/2}^0 f. \qquad (7)$$

Proposition 2 *The fractional derivative of order (α', τ') of an (α, τ)-fractional spline is another $(\alpha-\alpha', \tau-\tau')$-fractional spline:*

$$\partial_{\tau'}^{\alpha'} \beta_\tau^\alpha(t) = \sum_k (-1)^k \left| \begin{matrix} \alpha' \\ k-\tau' \end{matrix} \right| \beta_{\tau-\tau'}^{\alpha-\alpha'}(t-k). \tag{8}$$

In particular, making use of (7)*, we have*

$$\mathscr{H} \beta_\tau^\alpha(t) = \sum_k \frac{1}{\pi(k-\frac{1}{2})} \beta_{\tau-1/2}^\alpha(t-k). \tag{9}$$

This property will be used later when we show how to solve a fractional differential equation using a discrete wavelet transform. Notice that the coefficients used in (8) are the impulse response of the filter $2^{\alpha'-1} H_{\tau'}^{\alpha'-1}(-z)$.

4. WAVELETS AND DWT

Multiresolution analysis involves wavelet spaces that are usually chosen orthogonal to the multiresolution spaces. Denoting by

$$A^\alpha(e^{j\omega}) = \sum_k |\hat{\beta}_\tau^\alpha(\omega + 2k\pi)|^2$$

the discrete Fourier transform of the autocorrelation sequence of an (α, τ)-fractional B-spline, the Fourier transform of the semi-orthogonal fractional spline wavelet is given by

$$\begin{aligned} \hat{\psi}_\tau^\alpha(\omega) &= \frac{G_\tau^\alpha(e^{j\frac{\omega}{2}})}{2} \hat{\beta}_\tau^\alpha\left(\frac{\omega}{2}\right) \\ \text{where } G_\tau^\alpha(e^{j\omega}) &= -e^{-j\omega} H_\tau^\alpha(-e^{-j\omega}) A^\alpha(-e^{j\omega}). \end{aligned} \tag{10}$$

This extends the construction given in [10]. Equation (10) is the Fourier transform of the standard wavelet equation, where $G(z)$ is the wavelet filter. It can moreover be verified that ψ_τ^α and its integer shifts are orthogonal to $\{\beta_\tau^\alpha(t-k)\}_{k\in\mathbb{Z}}$.

Now that we have the scaling filter $H_\tau^\alpha(z)$ and the wavelet filter $G_\tau^\alpha(z)$, we can build an iterated dyadic filterbank which computes the discrete wavelet coefficients $\langle f, (\psi_\tau^\alpha)_{i,k} \rangle$, where $(\psi_\tau^\alpha)_{i,k}(t)$ is short fo $2^{-i/2}\psi_\tau^\alpha(2^{-i}t-k)$. The synthesis filters can easily be obtained using standard inversion formulæ [7] and are denoted $\mathring{H}_\tau^\alpha(z)$ and $\mathring{G}_\tau^\alpha(z)$. The fractional B-splines and the semi-orthogonal wavelets can also be orthonormalized, yielding an orthonormal set of filters, a case that is not discussed any further here.

In all cases, we have direct formulæ for the frequency response of these filters which is all we need to implement the wavelet transform *exactly* under periodic boundary conditions [2].

An interesting property is that these wavelets behave like fractional differentiation operators.

Proposition 3 *For a predominantly lowpass function f, the wavelet transform coefficient*

$$\langle f, (\psi_\tau^\alpha)_{i,k} \rangle \approx -2^{i(\alpha+3/2)} \frac{A^\alpha(\pi)}{4^{\alpha+1}} \partial_\tau^{\alpha+1} f(k2^i)$$

behaves like a generalized fractional derivative of order $\alpha+1$ and shift τ, evaluated at the point $k2^i$.

The interpretation of this result is that $|\omega|^{-\frac{\alpha+1}{2}}$-type noises are whitened by the DWT. The α-knob of the fractional spline wavelet transform might thus be an interesting tuning parameter for decorrelating these types of self-similar signals.

As an example, we show here that our fractional spline wavelet transform can also be used to deconvolve the equation $\partial_{\tau'}^{\alpha'} f = g$. If $f_{i,k}$ are the unknown coefficients of the (α, τ)- fractional spline wavelet decomposition of $f(t)$, we should have

$$g(t) = \sum_{i,k} f_{i,k} 2^{-i\alpha'} \partial_{\tau'}^{\alpha'} \psi_\tau^\alpha(2^{-i}t-k).$$

This shows that the coefficients $f_{i,k}$ are given by the wavelet decomposition of $g(t)$ with the wavelet $\partial_{\tau'}^{\alpha'} \psi_\tau^\alpha$. By Proposition 2, we know that $\partial_{\tau'}^{\alpha'} \psi_\tau^\alpha$ is a fractional spline of degree $\alpha-\alpha'$ and shift $\tau-\tau'$. The wavelet coefficients of $g(t)$ are thus obtained by iterating the analysis iterated filterbank that matches the synthesis filterbank that has $H(z) = H_{\tau-\tau'}^{\alpha-\alpha'}(z)$ as scaling filter, and $G(z) = 2^{2\alpha'-1} H_{\tau'}^{\alpha'-1}(-z) G_\tau^\alpha(z)$ as wavelet filter. The corresponding analysis filters follow using standard inversion relations and we get

- low-pass filter: $\tilde{H}(z) = H_{\tau'-\tau}^{\alpha+\alpha'}(z) \dfrac{A^\alpha(z)}{A^\alpha(z^2)}$;
- high-pass filter: $\tilde{G}(z) = -\dfrac{2^{-2\alpha'+1} z H_{\tau-\tau'}^{\alpha-\alpha'}(-z)}{A^\alpha(z^2)}$.

The implementation of this method is shown in Fig. 3; it is then possible to use all kinds of regularizations in the wavelet subbands. Using the same method, we can also generate a fractional Brownian motion. This is because a fBm is a random process that can be seen as the solution B of the differential equation $\partial_0^{h+1/2} B = \epsilon$ where ϵ is a white Gaussian noise [4].

5. CONCLUSION

We have presented a new complete set of scaling functions that depend on two parameters that can be tuned independently. What characterizes the associated multiresolutions are their versatility and flexibility. Adjusting the free parameters gives a way to optimize the basis functions for many problems that are currently solved by means of wavelets

such as source compression, denoising and deconvolution. Online Java demos of the (α, τ)-fractional wavelet transform are available on our website:

`bigwww.epfl.ch/demo/jfractsplinewavelet/`

6. REFERENCES

[1] M. Unser and T. Blu, "Fractional splines and wavelets," *SIAM Review*, vol. 42, no. 1, pp. 43–67, January 2000.

[2] T. Blu and M. Unser, "The fractional spline wavelet transform: Definition and implementation," in *Proc. IEEE Int. Conf. Acoust., Speech, Signal Process.*, Istanbul, Turkey, June 2000, vol. I, pp. 512–515.

[3] P. Flandrin, "Wavelet analysis and synthesis of fractional Brownian motion," *IEEE Trans. Inform. Th.*, vol. 38, no. 2, pp. 910–917, 1992.

[4] Y. Meyer, F. Sellan, and M. Taqqu, "Wavelets, generalized white noise and fractional integration: The synthesis of fractional Brownian motion," *J. Fourier Anal. Appl.*, vol. 5, no. 5, pp. 465–494, 1999.

[5] I.J. Schoenberg, "Contribution to the problem of approximation of equidistant data by analytic functions," *Quart. Appl. Math.*, vol. 4, pp. 45–99 and 112–141, 1946.

[6] M. Unser, "Splines: A perfect fit for signal and image processing," *IEEE Signal Process. Mag.*, vol. 16, no. 6, pp. 22–38, November 1999.

[7] G. Strang and T.Q. Nguyen, *Wavelets and Filter Banks*, Wellesley-Cambridge Press, Cambridge MA, 1996.

[8] S. Mallat, "A theory for multiresolution signal decomposition: The wavelet decomposition," *IEEE Trans. Pattern Anal. Mach. Intell.*, vol. 11, no. 7, pp. 674–693, July 1989.

[9] J. Liouville, "Sur une formule pour les différentielles à indices quelconques à l'occasion d'un mémoire de M. Tortolini," *J. Math. Pures Appl.*, vol. 20, pp. unnumbered, 1855, In French.

[10] M. Unser, A. Aldroubi, and M. Eden, "On the asymptotic convergence of B-spline wavelets to Gabor functions," *IEEE Trans. Inform. Th.*, vol. 38, no. 2, pp. 864–872, March 1992.

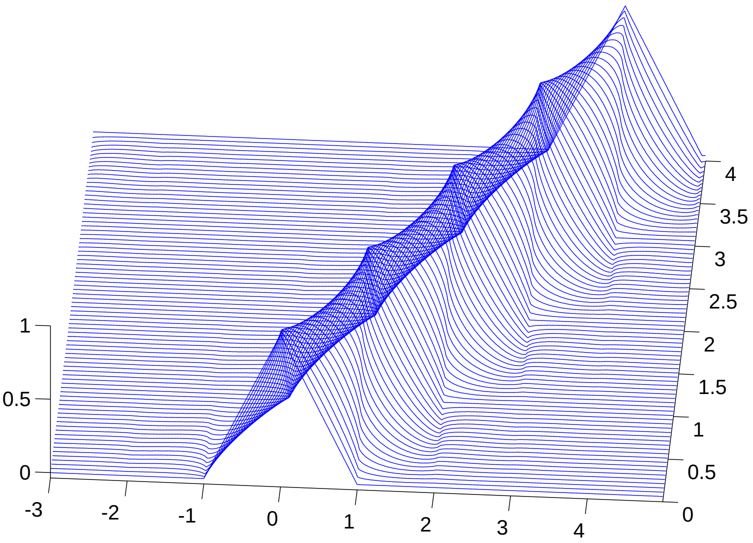

Fig. 1. Plot of the "linear" B-splines β^1_τ, for different values of $\tau \in [0, 4]$. Note that the functions with integer τ ($\tau = 0, 1, 2, 3$ and 4) are the usual linear spline; i.e., the triangle function.

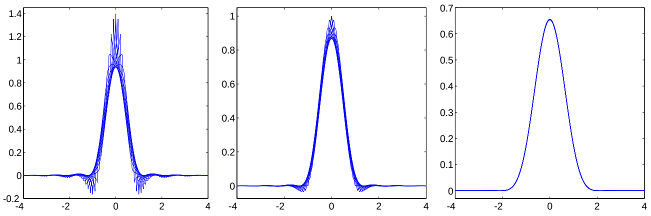

Fig. 2. Plots of $\beta^\alpha_\tau(t + \tau)$, for three different values of α; on each plot, τ varies in $[0, 1]$. This shows that $\beta^\alpha_\tau(t)$ gets closer to $\beta^\alpha_0(t - \tau)$ as α increases, which justifies the interpretation of τ as a shift parameter.

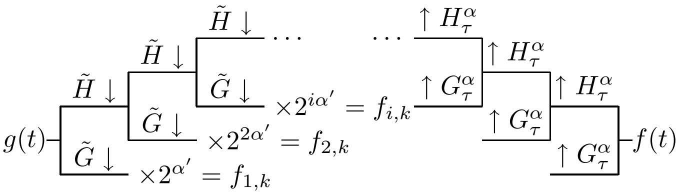

Fig. 3. Resolution of the differential equation $\partial^{\alpha'}_{\tau'} f = g$ using a dyadic analysis-synthesis filterbank. See the text for the exact values of $\tilde{H}$ and $\tilde{G}$.
