# Supplementary material for: Rhythms and Background (RnB): The Spectroscopy of Sleep Recordings
Source: eNeuro. 2026 Feb 3;13(2):ENEURO.0235-25.2025. doi: 10.1523/ENEURO.0235-25.2025 (PMC12867552; doi:10.1523/ENEURO.0235-25.2025)
Supplement: Data 1 — Download Data 1, ZIP file. [file eneuro-13-ENEURO.0235-25.2025-s002.zip › RnB-Wavelet-main/wRnBmain/rnb_wavelet/rnb/FracSpline/references/Self-Similarity__Part_ISplines_and_Operators__Michael_Unser_and_Thierry_Blu.pdf]

Self-Similarity: Part I—Splines and Operators

Michael Unser, *Fellow, IEEE*, and Thierry Blu, *Senior Member, IEEE*

***Abstract*—The central theme of this pair of papers (Parts I and II in this issue) is *self-similarity*, which is used as a bridge for connecting splines and fractals. The first part of the investigation is deterministic, and the context is that of L-splines; these are defined in the following terms: $s(t)$ is a cardinal L-spline iff $\mathrm{L}\{s(t)\} = \sum_{k\in\mathbb{Z}} a[k]\delta(t-k)$, where L is a suitable pseudodifferential operator. Our starting point for the construction of "self-similar" splines is the identification of the class of differential operators L that are both translation and scale invariant. This results into a two-parameter family of generalized fractional derivatives, ∂_τ^γ, where γ is the order of the derivative and τ is an additional phase factor. We specify the corresponding L-splines, which yield an extended class of fractional splines. The operator ∂_τ^γ is used to define a scale-invariant energy measure—the squared L_2-norm of the γth derivative of the signal—which provides a regularization functional for interpolating or fitting the noisy samples of a signal. We prove that the corresponding variational (or smoothing) spline estimator is a cardinal fractional spline of order 2γ, which admits a stable representation in a B-spline basis. We characterize the equivalent frequency response of the estimator and show that it closely matches that of a classical Butterworth filter of order 2γ. We also establish a formal link between the regularization parameter λ and the cutoff frequency of the smoothing spline filter: $\omega_0 \approx \lambda^{-2\gamma}$. Finally, we present an efficient computational solution to the fractional smoothing spline problem: It uses the fast Fourier transform and takes advantage of the multiresolution properties of the underlying basis functions.**

***Index Terms*—Fractals, fractional derivatives, fractional splines, interpolation, self-similarity, smoothing splines, Tikhonov regularization.**

I. Introduction

The concept of self-similarity is intimately linked to fractals [1]. It is a property that often results in a complex, highly irregular appearance, even though fractal patterns are typically constructed using simple generative rules. The classical man-made fractals, such as von Koch's snowflake or Sierpinski's triangle, are deterministic and literally self-similar in the sense that the whole is made up of smaller copies of itself. Nature provides many examples of nondeterministic fractals that are self-similar in a statistical sense over a wide range of scales [1], [2]. Fractional Brownian motion (fBm) is a prime example of a stochastic process that is statistically self-similar [3]. fBms are used to model phenomena in a variety of disciplines, including communications and signal processing [4].

Manuscript received October 12, 2005; revised May 27, 2006. The associate editor coordinating the review of this paper and approving it for publication was Dr. Timothy N. Davidson. This work is funded in part by the grant 200020-101821 from the Swiss National Science Foundation.

The authors are with the Biomedical Imaging Group, Ecole Polytechnique Fédérale de Lausanne (EPEL), CH-1015 Lausanne, Switzerland (e-mail: michael.unser@epfl.ch).

Color versions of one or more of the figures in this paper are available online at http://ieeexplore.ieee.org.

Digital Object Identifier 10.1109/TSP.2006.890843

An important property of fBm and related processes is that they can be easily transformed into stationary processes via the application of simple differential operators—such as finite differences [5], [6], derivatives [7], or even a wavelet transform [8]—or, alternatively, via Lamperti's transformation [9]. This has important practical repercussions, for it greatly simplifies their analysis. In recent years, wavelets have emerged as the preferred tool for analyzing fractal-like processes [10]–[12]. The approach was pioneered by Flandrin who proved that the wavelet transform would decompose an fBm-like process into stationary components that are essentially decorrelated [8]. There is an earlier, closely related result by Wornell that states that the wavelet transform is a good approximation of the Karhunen–Loève transform for the class of stationary processes with near $1/\omega$ behavior [13]. Interestingly, Mallat's landmark paper on wavelets also contains an early application of wavelets to the estimation of the fractal dimension of a signal [14]. The link between fractals and wavelets is very strong and is further supported by the following remarkable wavelet properties:

- a wavelet analysis is equivalent to a multiscale differentiation [15]; this implies that the wavelet coefficients of an fBm at a given scale define a discrete-time stationary process;
- the structure of the decomposition is self-similar by construction: the basis functions are dilated versions of each other [14];
- the basis functions themselves are fractals [16].

For an in-depth coverage of the notion of self-similarity within the context of wavelets and refinement equations, we refer to the monograph of Cabrelli *et al.* [17].

The above results implicitly suggest that there should also be a connection with splines because of the essential role these play in wavelet theory. Indeed, any scaling function (or wavelet) can be written as the convolution of a polynomial B-spline and a singular distribution, with the spline component being responsible for all important mathematical properties: vanishing moments, multiscale differentiation property, order of approximation and regularity [18]. Another relevant fact is that Schoenberg's classical polynomial splines [19] are made up of self-similar building blocks [16]: the one-sided power functions $t_+^n = \max(0,t)^n$, which are elementary fractals.[1]

The notion of splines, however, need not be restricted to piecewise polynomial functions. More generally, we view them as a mathematical framework for linking the continuous and the discrete [20], [21]. This idea can be made explicit by defining generalized cardinal L-splines for which the continuous-time operator L plays the role of a mathematical analog-to-discrete

[1]The function $f(t) = t_+^n$ is homogeneous with respect to dilation in the sense that there exists $\lambda \in \mathbb{R}$ such that $f(t/a) = \lambda \cdot f(t)$.

1053-587X/$25.00 © 2007 IEEE

converter (cf. Section II). We believe that this more abstract, operator-based formulation is the key to gaining a deeper understanding of these entities. It also suggests a deductive paradigm by which splines can be constructed starting from first principles: i.e., the specification of a class of differential operators L with some relevant invariance properties.

Our purpose in this pair of papers (Parts I and II in this issue) is to demonstrate this approach by focusing on the important case where the spline-defining operator is scale invariant. As in the case of fractals, there are two complementary aspects to the problem—deterministic and stochastic—which are treated in Part I and Part II, respectively. The second part, in particular, will focus on the minimum mean-square error (MMSE) estimation of fractal-like processes, which calls for a specialized mathematical treatment; this will allow us to establish a fundamental connection between the fractional splines, which will be identified in the first part, and fBms.

The present paper, whose context is purely deterministic, is organized as follows. In Section II, we set the stage by reinterpreting the elementary example of a piecewise constant function as a D-spline, where D is the derivative operator. We then define cardinal L-splines in the general shift-invariant setting and briefly review their main deterministic properties. In the process, we also propose a new, extended smoothing spline estimator that minimizes a quadratic, convolution-weighted error criterion (data term) subject to a regularization constraint that favors solutions with small "spline energies." The important practical point is that the general solution of this problem is an L*L-spline whose B-spline coefficients can be determined by suitable filtering of the noisy discrete input signal. In Section III, we turn our attention to spline-defining operators L that are self-similar. We prove that this class reduces to fractional derivatives of order γ, which leads to the identification of a corresponding two-parameter family of fractional splines, extending an earlier construction of ours [22]. We also characterize the nonlocal effect of our extended fractional derivatives for Schwartz's class of rapidly decreasing functions. In Section IV, we specify the corresponding fractional smoothing spline estimators and characterize their equivalent frequency response. We then present an efficient fast Fourier transform (FFT)-based computational solution, which takes advantage of the multiresolution properties of the underlying basis functions. We conclude this first part with a brief discussion of the "scale-invariance" properties of the various fractional spline estimators that can be specified within the proposed variational framework.

II. Generalized Splines

The purpose of this section is to present a generalized notion of splines that is associated with a particular class of differential operators L. We start with a simple introductory example that explains the key ideas behind this type of construction. We then proceed with a general characterization of cardinal L-splines along the lines of [23]. We recall their key properties and introduce an extended convolution-weighted smoothing spline algorithm for fitting discrete signal samples corrupted by noise.

A. Introductory Example: D-Splines or Piecewise-Constant Functions

Let $\mathrm{D} = (d/dt)$ denote the first-order derivative operator. A piecewise constant spline can be formally viewed as a function $s(t)$ whose derivative is a weighted stream of Dirac distributions

$$\mathrm{D}s(t) = \sum_{k\in\mathbb{Z}} a_k \delta(t - t_k)$$

where the t_k's encode the locations of the spline discontinuities (or knots). In this paper, we concentrate on the *cardinal* setting where the knots are on the integers (i.e., $t_k = k$) and write $a[k] = a_k$ to signify that the differentiated cardinal spline $\mathrm{D}s(t)$ has the structure of a sampled signal $\sum_{k\in\mathbb{Z}} a[k]\delta(t-k)$. Starting from there, we reconstruct the spline by applying the inverse operator D^{-1}, which amounts to an integration. Thus, by using the well-known fact that $\mathrm{D}^{-1}\{\delta\} = u(t)$ (the unit step), we obtain the explicit formula

$$s(t) = p_0 + \sum_{k\in\mathbb{Z}} a[k]u(t-k) \tag{1}$$

where p_0 is a suitable integration constant. Equation (1) clearly indicates that $s(t)$ is piecewise constant with discontinuities at the integers or, equivalently, a cardinal polynomial spline of degree 0. The important point to note here is that the basis function generator $u(t)$ is the causal *Green function*[2] of D and that the additional term p_0 (a constant) is a signal that is in the null space of D. In practice, one usually prefers an equivalent and much simpler representation in terms of shifted B-spline basis functions

$$s(t) = \sum_{k\in\mathbb{Z}} c[k]\beta_+^0(t-k) \tag{2}$$

where $\beta_+^0(t)$ is the B-spline of degree 0 (causal rect function) that can be expressed as

$$\beta_+^0(t) = \Delta_+ u(t) = u(t) - u(t-1) \tag{3}$$

where Δ_+ is the causal finite-difference operator. By plugging (3) into (2), we can relate the coefficients of the representations (1) and (2) via the difference equation $a[k] = c[k] - c[k-1]$. Moreover, it is easy to establish the following B-spline reproduction formulas:

$$\sum_{k\in\mathbb{Z}} \beta_+^0(t-k) = 1$$
$$\sum_{l\in\mathbb{Z}} u(l-k)\beta_+^0(t-l) = u(t-k)$$

which links the two kinds of basis functions. This whole set of relations is illustrated in Fig. 1. For computational purposes, representation (2) is obviously much more attractive than (1) because the B-spline basis functions are *localized* as opposed to the ones in (1), which are infinitely supported and nondecreasing. In addition, the piecewise-constant basis functions $\{\beta_+^0(t-k)\}_{k\mathbb{Z}}$ are orthogonal, which has many advantages, with stability being not the least. The "magical"

[2]By definition, $\rho(t)$ is a Green function of the shift-invariant operator L if and only if $\mathrm{L}\{\rho\} = \delta(t)$.

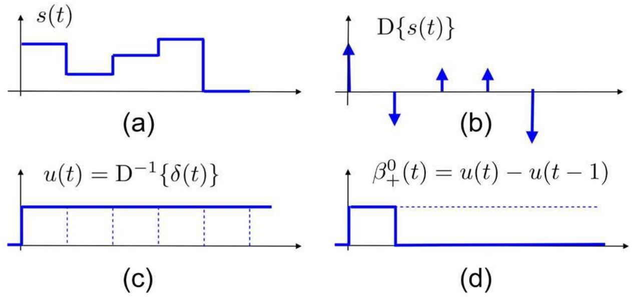

Fig. 1. Piecewise constant splines. (a) and (b): Interpretration of $s(t)$ as a D-spline. (c) Representation of the step function (Green function of the operator D) as a weighted sum of B-splines of degree 0. (d) B-spline of degree 0 as the difference of two step functions.

trick that allowed us to switch from the badly conditioned basis functions in (1) to the much nicer ones in (2) is contained in the localization formula $\beta_+^0(t) = \Delta_+ u(t)$, where $u(t) = \mathrm{D}^{-1}\{\delta\}$. In essence, we are using digital means—the finite-difference operator Δ_+—to approximately undo the effect of the integrator that is applied to δ. In other words, the B-spline $\beta_+^0(t)$ may be thought of as some kind of approximation of the Dirac impulse within the space of cardinal piecewise constant splines, or equivalently, the space that is spanned by the integer shifts of the Green function of D. While this way of describing the construction of piecewise-constant functions may seem contrived and much more complicated than necessary, it is extremely fruitful conceptually because it lends itself naturally to generalization. Basically, we will now replace the derivative operator D by some pseudodifferential operator L and apply the exact same recipe to define an extended family of generalized splines.

B. Spline-Admissible Operators

Following [23], we introduce the notion of "spline-admissible" operator of order r.

Definition 1: L is a *spline-admissible* operator of order $r > 1/2$ if and only if the following conditions are met.

1) L is a linear, shift-invariant operator with a frequency response $\hat{L}(\omega)$ such that
$$\sum_{n\in\mathbb{Z}} \frac{|\omega + 2n\pi|^{2s}}{1 + \left|\hat{L}(\omega + 2n\pi)\right|^2} \leq C_s < +\infty \tag{4}$$
for all positive real $s < r - 1/2$.

2) L has a well-defined inverse L^{-1} (not necessarily unique) whose impulse response $\rho(t) \in \mathcal{S}'$ is a function of slow growth included in Schwartz's class of tempered distributions. Thus, L admits $\rho(t)$ as Green function: $\mathrm{L}\{\rho\} = \delta(t)$.

3) There exists a corresponding spline-generating function $\beta(t) = \Delta_\mathrm{L}\{\rho(t)\} := \sum_{k\in\mathbb{Z}} d[k]\rho(t-k)$ (generalized B-spline) that is sufficiently localized for $\sup_{t\in[0,1)} \sum_{k\in\mathbb{Z}} |\beta(t-k)| < +\infty$. In particular, this implies that $\beta(t) \in L_p$ for all $1 \leq p \leq \infty$ and that its integer samples are in ℓ_1.

4) The localization operator Δ_L in 3) is a stable digital filter in the sense that $d \in \ell_1$.

5) The functions $\{\beta(t-k)\}_{k\in\mathbb{Z}}$ form an L_p-stable Riesz basis. Specifically, the following two conditions must be satisfied for all $1 \leq p \leq \infty$:
$$\inf_{\|c\|_{\ell_p}=1} \left\| \sum_{k\in\mathbb{Z}} c[k]\beta(t-k) \right\|_{L_p} > 0$$
$$\text{and} \quad \sup_{\|c\|_{\ell_p}=1} \left\| \sum_{k\in\mathbb{Z}} c[k]\beta(t-k) \right\|_{L_p} < \infty.$$

Conditions 1) to 4) are quite explicit and not too difficult to check in practice. Condition 1) signifies that L has qualitatively the same behavior as a derivative of order r [23]. One usually has some latitude for the choice of the localization operator Δ_L: in essence, it is a digital filter that should be designed such that its frequency response $\Delta_\mathrm{L}(e^{j\omega}) = \sum_{k\in\mathbb{Z}} d[k]e^{-j\omega k}$ closely matches the behavior of $\hat{L}(\omega)$, especially around the frequencies where L is singular. Indeed, we want the Fourier transform of our generalized B-spline
$$\hat{\beta}(\omega) := \int_{-\infty}^{+\infty} \beta(t)e^{-j\omega t}\mathrm{d}t = \frac{\Delta_\mathrm{L}(e^{j\omega})}{\hat{L}(\omega)} \tag{5}$$
to be close to one over a reasonable frequency range (remember: our goal is to approximate δ) and have the largest possible degree of differentiation (ideally, $\beta \in C^\infty$) to ensure that β has fast decay (ideally, compact support). The absolute summability condition in 3) is required for technical purposes and is automatically satisfied when $\beta(t)$ is bounded and compactly supported, which will often be the case when Δ_L is properly chosen. Condition 5) is less direct and typically needs to be checked on a case-by-case basis. In fact, because of the summability requirement in 3), it is sufficient to satisfy the standard Riesz basis condition [24]. Specifically, one needs to prove that the L_2-Riesz bounds $\inf_{\omega\in[0,2\pi)} \sqrt{A_\mathrm{L}(e^{j\omega})}$ and $\sup_{\omega\in[0,2\pi)} \sqrt{A_\mathrm{L}(e^{j\omega})}$ are strictly positive and finite, where
$$A_\mathrm{L}(e^{j\omega}) = \sum_{n\in\mathbb{Z}} \left|\hat{\beta}(\omega + 2\pi n)\right|^2. \tag{6}$$

C. Cardinal L*-Splines*

Having specified the properties of a spline-admissible operator L, we now proceed with the specification of the corresponding family of cardinal spline functions.

Definition 2: The continuous-time function $s(t)$, $t \in \mathbb{R}$, is a cardinal L-spline if and only if
$$\mathrm{L}s(t) = \sum_{k\in\mathbb{Z}} a[k]\delta(t-k) \tag{7}$$
with $a[k] \in \ell_\infty$.

Now, if L is spline-admissible with generator $\beta(t)$, we can readily define the corresponding generalized spline subspace of L_p with $1 \leq p \leq \infty$
$$\mathcal{V}^p(\beta) = \left\{ s(t) = \sum_{k\in\mathbb{Z}} c[k]\beta(t-k) : c[k] \in \ell_p \right\} \tag{8}$$

and we have the guarantee that each spline in $\mathcal{V}^p(\beta)$ is uniquely characterized by its B-spline coefficients $c[k]$. Moreover, the expansion coefficients in (7) are given by $a[k] = (d * c)[k]$, where d is the digital filter representation of the localization operator; i.e., $\Delta_{\mathrm{L}}\{\delta(t)\} = \sum_{k\in\mathbb{Z}} d[k]\delta(t-k)$.

To illustrate the method, we now consider a slightly more general version of our introductory example with $\mathrm{L} = \mathrm{D}^{n+1}$. The causal Green function of D^{n+1} is the one-sided power function $t_+^n/n!$ (the impulse response of the $(n+1)$-fold integrator) with $t_+ = \max(0,t)$. The frequency response of the $(n+1)$th-order differentiator is $(j\omega)^{n+1}$, and one easily checks that its smoothness order (as specified in (4)) is $(n+1)$ as well. The classical discrete version of this operator is the $(n+1)$th-order finite difference Δ_+^{n+1} whose frequency response is $(1-e^{-j\omega})^{n+1}$. By applying this localization operator to the Green function of D^{n+1}, we obtain Schoenberg's classical formula for the B-spline of degree $n: \beta_+^n(t) = \Delta_+^{n+1} t_+^n/n!$. The last step is to make sure that this B-spline generates a stable Riesz basis, which is indeed the case [22], [25]. From the above, we immediately deduce that the underlying D^{n+1}-splines are in fact equivalent to the classical polynomial ones, which have the following key properties.

1) They are polynomials of degree n within each interval $[k, k+1)$; this becomes more apparent if we consider their representation in terms of shifted one-sided power functions: $s(t) = \sum_{k\in\mathbb{Z}} a[k](t-k)_+^n/n!$.
2) They are $(n-1)$ times continuously differentiable; this follows from the property that the $(n-1)$th derivative of each of the basic Green atoms is a continuous piecewise-linear function: $\mathrm{D}^{n-1}\{(t-k)_+^n/n!\} = (t-k)_+$.
3) They have a stable representation in the cardinal B-spline basis $\{\beta_+^n(t-k)\}_{k\in\mathbb{Z}}$.

D. Variational Splines and Best Interpolants

The spline-defining operator L can also be used to measure the "spline energy" of a function $f(t)$: $\|\mathrm{L}f\|_{L_2}^2$. This quantity is well defined as long as $f \in \mathcal{W}_2^{\mathrm{L}}$, where $\mathcal{W}_2^{\mathrm{L}}$ denotes the generalized Sobolev space associated with the operator L [23]. In the sequel, we will use this spline energy as a regularization term to constrain and specify some general data fitting problems.

It turns out that this spline energy naturally leads to the definition of a corresponding L*L-spline that is optimal in a well-defined variational sense. If L is spline admissible of order $r > (1/2)$ with generator β, then we can prove the following important properties for the corresponding class of L*L-splines (cf. [23]).

1) The operator L*L is guaranteed to be spline admissible of order $r' \geq 2r - 1/2$ with symmetric generator $\varphi(t) = \beta(t) * \beta(-t)$.
2) Any given discrete signal $f[k] \in \ell_p$ has a unique, well-defined L*L-spline interpolant in $\mathcal{V}^p(\varphi)$ as specified in (8).
3) For any function $f \in \mathcal{W}_2^{\mathrm{L}}$, the spline energy can be decomposed as

$$\|\mathrm{L}f\|_{L_2}^2 = \|\mathrm{L}s_{\mathrm{int}}\|_{L_2}^2 + \|\mathrm{L}\{f - s_{\mathrm{int}}\}\|_{L_2}^2 \tag{9}$$

where $s_{\mathrm{int}}(t)$ is the unique L*L-spline that interpolates $f(t)$, i.e., $f(k) = s_{\mathrm{int}}(k), \forall k \in \mathbb{Z}$.

A first, direct practical implication of these properties is the following key result, which yields an "optimal" procedure for interpolating a discrete signal, together with a simple digital filtering algorithm.

Theorem 1: Let $f[k] \in \ell_2$ be a discrete input signal and L be a spline-admissible operator of order $r > 1/2$ with generator $\beta(t)$. Among all possible interpolating functions $f(t) \in \mathcal{W}_2^{\mathrm{L}}$, the optimal one that minimizes $\|\mathrm{L}f\|_{L_2}$, subject to the interpolation constraint $f(t)|_{t=k} = f[k]$, is the L*L-spline interpolant

$$s_{\mathrm{int}}(t) = \sum_{k\in\mathbb{Z}} (h_{\mathrm{int}} * f)[k]\varphi(t-k)$$

where $\varphi(t) = \beta(t) * \beta(-t)$ and where $h_{\mathrm{int}} \in \ell_1$ is the impulse response of a bounded-input bounded-output (BIBO) stable filter whose frequency response is

$$H_{\mathrm{int}}(e^{j\omega}) = \frac{1}{\sum_{k\in\mathbb{Z}} \varphi(k) e^{-j\omega k}} = \frac{1}{A_{\mathrm{L}}(e^{j\omega})} \tag{10}$$

where $A_{\mathrm{L}}(e^{j\omega})$ is defined by (6). The proof of these results can be found in [23]. Note that the denominator of (10) is nonvanishing because of the Riesz basis condition. An important special case is $\mathrm{L} = \mathrm{D}^2$, which leads to the classical result that the cubic spline interpolant (with $\mathrm{L}^*\mathrm{L} = \mathrm{D}^4$) is the minimum curvature solution for it minimizes the energy of the second derivative.

E. Generalized Smoothing Splines

When the input data $\{y[k] = f(k) + n[k]\}_{k\in\mathbb{Z}}$ is corrupted by discrete noise n, it may be counterproductive to determine its exact spline fit. Instead, one should rather seek a solution that is close to the data but has some inherent smoothness to counterbalance the effect of the noise. To this end, one usually specifies a regularized version of the interpolation problem that involves a compromise between a data term—the quadratic fitting error—and a regularization term $\|\mathrm{L}f\|_{L_2}^2$ that limits the spline energy of the solution, which is then called a *smoothing spline* [26], [27]. The relative weight between the two components of the criterion is adjusted by means of a regularization factor $\lambda > 0$. Here, we consider an extension of the standard smoothing spline algorithm where the fitting error is weighted in the frequency domain, which corresponds to the convolution with a discrete weighting filter v in the data domain. A remarkable result is that the solution of this approximation problem, among all possible continuous-time functions $s(t)$, is a cardinal L*L-spline and that it can be determined by digital filtering.

Before stating our result, we set our class of admissible weighting filters to those satisfying $|V(e^{j\omega})| < +\infty$ for almost every $\omega \in [0, 2\pi]$. This ensures that $(v * y)[k]$ is square summable whenever $y[k] \in \ell_2$ (as a consequence of Parseval's relation).

Theorem 2: Let L be a spline-admissible operator of regularity $r > (1/2)$ with spline generator $\beta(t)$ such that $\mathrm{L}\{\beta(t)\} = \Delta_{\mathrm{L}}\{\delta(t)\} = \sum_{k\in\mathbb{Z}} d[k]\delta(t-k)$ with $d \in \ell_1$. Then, the continuous-time solution of the variational problem with discrete

input data $y[k] \in \ell_2$, admissible weighting filter $v[k]$, and regularization parameter $\lambda \geq 0$

$$\min_{s(t)\in L_2} \sum_{k\in\mathbb{Z}} [v[k] * (y[k] - s(k))]^2 + \lambda \|\mathrm{L}s\|_{L_2}^2$$

is the cardinal L*L-spline that is specified by

$$s(t) = \sum_{k\in\mathbb{Z}} (h * y)[k]\varphi(t-k) \tag{11}$$

where $\varphi(t) = \beta(t) * \beta(-t)$ and where $h \in \ell_2$ is the impulse response of the digital smoothing spline filter whose frequency response is

$$H(e^{j\omega}) = \frac{1}{A_{\mathrm{L}}(e^{j\omega}) + \lambda \frac{|\Delta_{\mathrm{L}}(e^{j\omega})|^2}{|V(e^{j\omega})|^2}}. \tag{12}$$

Proof: A necessary condition for the criterion to be finite is obviously $\|\mathrm{L}s\|_{L_2}^2 < +\infty$. This, together with the requirement $s(t) \in L_2$, implies that the solution is necessarily in $\mathcal{W}_2^{\mathrm{L}} \subset L_2$ [23]. Thus, we can use (9) and write the criterion to minimize as

$$\underbrace{\|v[k] * (y[k] - s(k))\|_{\ell^2}^2}_{\xi_1} + \lambda \underbrace{\|\mathrm{L}\{s_{\mathrm{int}}(t)\}\|_{L_2}^2}_{\xi_2} + \lambda \|\mathrm{L}\{s(t) - s_{\mathrm{int}}(t)\}\|_{L_2}^2$$

where $s_{\mathrm{int}}(t)$ is the L*L-spline interpolator of the sequence $s(k) \in \ell_2$. Note that the underbraced expressions, ξ_1 and ξ_2, are entirely specified by the integer samples $s(k)$. Moreover, using Parseval identity and the fact that $\hat{\varphi}(\omega) = |\Delta_{\mathrm{L}}(e^{j\omega})|^2/|\hat{L}(\omega)|^2$, we find that

$$\begin{aligned}
\xi_1 &= \frac{1}{2\pi}\int_0^{2\pi} \left| V(e^{j\omega})\left(Y(e^{j\omega}) - S(e^{j\omega})\right)\right|^2 \mathrm{d}\omega \\
\xi_2 &= \frac{1}{2\pi}\int_{-\infty}^{\infty} \left| S(e^{j\omega}) H_{\mathrm{int}}(e^{j\omega})\hat{L}(\omega)\hat{\varphi}(\omega)\right|^2 \mathrm{d}\omega \\
&= \frac{1}{2\pi}\int_{-\infty}^{\infty} \left|\frac{S(e^{j\omega})}{A_{\mathrm{L}}(e^{j\omega})}\right|^2 \frac{\left|\Delta_{\mathrm{L}}(e^{j\omega})\right|^4}{\left|\hat{L}(\omega)\right|^2} \mathrm{d}\omega
\end{aligned}$$

where $S(z) = \sum_{k\in\mathbb{Z}} s(k) z^{-k}$ and $A_{\mathrm{L}}(e^{j\omega}) = \sum_{n\in\mathbb{Z}} \hat{\varphi}(\omega + 2\pi n)$. The second term is further simplified to

$$\begin{aligned}
\xi_2 &= \frac{1}{2\pi}\int_{-\infty}^{\infty} \left|\frac{S(e^{j\omega})\Delta_{\mathrm{L}}(e^{j\omega})}{A_{\mathrm{L}}(e^{j\omega})}\right|^2 \hat{\varphi}(\omega)\mathrm{d}\omega \\
&= \frac{1}{2\pi}\int_0^{2\pi} \left|\frac{S(e^{j\omega})\Delta_{\mathrm{L}}(e^{j\omega})}{A_{\mathrm{L}}(e^{j\omega})}\right|^2 \sum_{n\in\mathbb{Z}} \hat{\varphi}(\omega + 2\pi n)\mathrm{d}\omega, \\
&= \frac{1}{2\pi}\int_0^{2\pi} \frac{\left|\Delta_{\mathrm{L}}(e^{j\omega})S(e^{j\omega})\right|^2}{A_{\mathrm{L}}(e^{j\omega})} \mathrm{d}\omega
\end{aligned}$$

Combining the above expressions, we rewrite the criterion to minimize as

$$\begin{aligned}
\xi_1 + \lambda\xi_2 = &\frac{1}{2\pi}\int_0^{2\pi} \frac{\left|V(e^{j\omega})\right|^2}{H(e^{j\omega})A_{\mathrm{L}}(e^{j\omega})} \\
&\times \left|S(e^{j\omega}) - H(e^{j\omega})A_{\mathrm{L}}(e^{j\omega})Y(e^{j\omega})\right|^2 \mathrm{d}\omega \\
&+ \frac{1}{2\pi}\int_0^{2\pi} \left|V(e^{j\omega})\right|^2 \left|Y(e^{j\omega})\right|^2 \\
&\times \left(1 - A_{\mathrm{L}}(e^{j\omega})H(e^{j\omega})\right)\mathrm{d}\omega
\end{aligned}$$

using the expression of $H(e^{j\omega})$ (12) for simplification purposes; the quantity $|V(e^{j\omega})|^2/(H(e^{j\omega})A_{\mathrm{L}}(e^{j\omega}))= |V(e^{j\omega})|^2 + \lambda(|\Delta_{\mathrm{L}}(e^{j\omega})|^2/A_{\mathrm{L}}(e^{j\omega})$ is bounded from above, due to our assumptions. The key step here has been to combine the arguments of the integral into a square (first term) plus a correction term that is independent upon the unknown $S(e^{j\omega})$.

The criterion $\xi_1 + \lambda\xi_2$ is clearly minimal iff $S(e^{j\omega}) - H(e^{j\omega})A_{\mathrm{L}}(e^{j\omega})Y(e^{j\omega}) = 0$; that is, when $s[k] = h[k] * (\varphi(k) * y[k])$, using the fact that $A_{\mathrm{L}}(e^{j\omega}) = \sum_k \varphi(k)e^{-jk\omega}$. On the other hand, $\lambda\|\mathrm{L}\{s(t) - s_{\mathrm{int}}(t)\}\|_{L_2}^2$ is minimal iff $s = s_{\mathrm{int}}$; i.e., iff s is an L*L spline.

As a consequence, the complete functional $\xi_1 + \lambda\xi_2 + \lambda\|\mathrm{L}\{s(t) - s_{\mathrm{int}}(t)\}\|_{L_2}^2$ is minimized for the L*L spline $s(t)$ whose samples satisfy $s[k] = h[k] * (\varphi(k) * y[k])$. This is precisely the solution (11) as one can check by setting $t = k$ in (11).

Note that the frequency response of the smoothing spline filter is bounded (ℓ_2-stability), irrespective of the value of $\lambda \geq 0$, because $1/H(e^{j\omega}) \geq \inf_\omega A_{\mathrm{L}}(e^{j\omega}) > 0$ (Riesz basis condition).

If we further add the restriction that $v[k] \in \ell_1$ and that its Fourier transform is bounded from below, then we have the guarantee that $h[k] \in \ell_1$ (BIBO stability), which comes a consequence of Wiener's Lemma (cf. [28, Ch. 13]). ■

The optimal solution $s(t)$ in Theorem 2 is a generalized version of the nonweighted smoothing spline described in [23]. By adjusting the regularization parameter λ, we can control the amount of smoothing. When $\lambda = 0$, there is no smoothing at all and the solution interpolates the data precisely and coincides with $s_{\mathrm{int}}(t)$ in Theorem 1, irrespective of the choice of weighting kernel v. For larger values of λ, the smoothing kicks in and typically tends to attenuate high frequency components. In the limit, when $\lambda \to +\infty$, it will preserve the signal components that are in the null space of the operator L; for instance, the best fitting polynomial of degree $n-1$ when $\mathrm{L} = \mathrm{D}^n$ (polynomial spline case).

The key practical question is how to select the most suitable operator L, the weighting kernel and the optimal value of λ for the problem at hand. While this can be done empirically, it can also be approached in a rigorous statistical fashion by introducing a stochastic model for the signal. Here, we will promote the use of fractional derivative operators of the type introduced next. In the companion paper, we will prove that this is the optimal approach for the estimation of fractal-like processes. We will also show how to optimally select the free parameters of the fractional smoothing spline filter: γ (order of the derivative), λ, and $|V(e^{j\omega})|^2$.

III. Scale-Invariant L-Splines

Since fractal-like signals are statistically self-similar, it is quite natural to investigate the class of differential operators that have the same type of invariance properties. We will characterize these operators and verify that they are spline admissible. We will also show that they yield an extended family of fractional splines—the so-called (α, τ)-splines—which are substantially richer than the ones initially proposed in [22].

A. Characterization of Scale-Invariant Operators

We will now characterize the special class of spline-admissible operators that are scale invariant.

Definition 3: A real operator L is scale invariant if and only if it commutes (up to some scaling constant C_T) with the dilation operation D_T: $\mathrm{D}_T \circ \mathrm{L} = C_T \cdot \mathrm{L} \circ \mathrm{D}_T$, where $\mathrm{D}_T\{s\}(t) = s(t/T)$ for any signal $s(t)$, and where $C_T = f(T)$ is a function of the dilation factor $T > 0$.

In the case of a convolution operator, we can rewrite the above scale-invariance condition in the frequency domain; this gets translated into the following condition on the frequency response of the operator: $\hat{L}(T\omega) = f(T)\hat{L}(\omega)$. Note that the function $f(T)$ has to be real in order to ensure that $\hat{L}(-\omega) = \hat{L}(\omega)^*$ (Hermitian symmetry). In fact, the choice of $f(T)$ is even more restricted, as shown next.

Proposition 1: A real scale-invariant convolution operator L is necessarily γth-order scale invariant; i.e., its frequency response is such that $\hat{L}(T\omega) = T^{\gamma}\hat{L}(\omega)$ for any $T > 0$, where $\gamma \in \mathbb{R}$.

Proof: We consider a scale-invariant convolution operator L. Because $\hat{L}(\omega)$ is a distribution, it acts as a linear functional on the test functions φ_k in Schwartz's class $\mathcal{S}$; it also satisfies the standard continuity condition: $\langle \hat{L}, \varphi_k \rangle \to \langle \hat{L}, \varphi \rangle$ when $\varphi_k \to \varphi$ as $k \to \infty$ [29]. This implies the continuity at $T = 1$ of the function $f(T)$ involved in Definition 3 as shown below:

- By making a change of variables, we have that $\langle \hat{L}(\omega T), \varphi(\omega) \rangle = \langle \hat{L}(\omega), T^{-1}\varphi(\omega T^{-1}) \rangle$. Using scale invariance, this proves that
$$f(T)\left\langle \hat{L}(\omega), \varphi(\omega) \right\rangle = \left\langle \hat{L}(\omega), T^{-1}\varphi(\omega T^{-1}) \right\rangle.$$
- The limit of $T^{-1}\varphi(\omega T^{-1})$ as $T \to 1$ is obviously $\varphi(\omega)$. So, using the continuity property of the distribution $\hat{L}(\omega)$, the right-hand side of the above equation tends to $\langle \hat{L}(\omega), \varphi(\omega) \rangle$. This proves that the left-hand side is convergent as well when $T \to 1$, and finally that $\lim_{T \to 1} f(T) = 1$.

In addition, it is easy to verify that $f(T)$ has to satisfy the chain rule $f(T_1 T_2) = f(T_1) f(T_2)$ by writing

$$\begin{aligned} f(T_1T_2)\hat{L}(\omega) &= \hat{L}(\omega T_1 T_2) \\ &= f(T_1)\hat{L}(\omega T_2) \\ &= f(T_1)f(T_2)\hat{L}(\omega). \end{aligned}$$

We can now turn to standard analysis to show that the functions that satisfy the chain rule $f(T_1T_2) = f(T_1)f(T_2)$ and are continuous at $T = 1$ are necessarily of the form $f(T) = T^{\gamma}$. Note that γ has to be real in order to ensure that $f(T)$ is real. ■

The γth-order scale-invariance property implies that the Green function of L is self-similar: $\rho(t/T) = T^{1-\gamma}\rho(t)$. This follows from the fact that the inverse operator L^{-1} is scale-invariant of order $-\gamma$; a fact that is easily established in the Fourier domain. The importance of scale-invariant operators is that they are the only ones that yield splines that are truly scale invariant in the sense that the defining operator remains the same irrespective of the scale (or knot spacing T). To put it more explicitly, we will say that a function $s(t)$ is a *scale-invariant* L-*spline* of order γ if and only if $\mathrm{L}\{s(t/T)\} = \sum_{k\in\mathbb{Z}} T^{1-\gamma} a[k]\delta(t - kT)$ with $a \in \ell_\infty$ for any scale $T > 0$. This is obviously only possible if L is spline admissible and scale invariant of order γ. Interestingly, it turns out that the only splines that are scale invariant are the fractional ones, which corresponds to the choice where L is a pure fractional derivative of order γ. This is a direct consequence of the following proposition.

Proposition 2: A convolution operator L is γth-order scale invariant if and only if its Fourier transform can be written (up to some real multiplicative factor) as

$$\hat{L}(\omega) = \hat{L}_{\gamma,\tau}(\omega) := (-j\omega)^{\frac{\gamma}{2}-\tau}(j\omega)^{\frac{\gamma}{2}+\tau} \quad (13)$$

where τ is an adjustable phase parameter. Moreover, for $\gamma > 1$, these fractional derivative operators are all spline admissible of order γ.

Proof: By differentiating $\hat{L}(T\omega) = T^{\gamma}\hat{L}(\omega)$ with respect to T and setting $T = 1$, we obtain the differential equation

$$\omega \frac{\mathrm{d}\hat{L}(\omega)}{\mathrm{d}\omega} = \gamma \hat{L}(\omega)$$

whose general solution can be shown to be (cf. [30])

$$\hat{L}(\omega) = \begin{cases} C_1\omega_+^{\gamma} + C_2(-\omega)_+^{\gamma} \\ \quad + C_3\delta^{(-\gamma-1)}(\omega), & \text{for } \gamma = -1, -2, -3, \dots \\ C_1\omega_+^{\gamma} + C_2(-\omega)_+^{\gamma}, & \text{otherwise} \end{cases}$$

where C_1, $C_2 \in \mathbb{C}$ are some arbitrary constants. In our case, we have the additional constraint (4), which rules out the possibility of $\hat{L}$ containing Diracs and implies $\gamma > (1/2)$. Moreover, because our operators are real, $\hat{L}$ has to satisfy the Hermitian symmetry $\hat{L}^*(-\omega) = \hat{L}(\omega)$ so that we can write the solution for $\omega \geq 0$ as $\hat{L}(\omega) = C_1\omega^{\gamma}$. This is equivalent to (13) provided that we set $C_1 = |C_1| \cdot e^{j\pi\tau}$ with the choice of normalization $|C_1| = 1$.

The Green functions of these operators are well defined and can be localized to yield the so-called $(\alpha - \tau)$ fractional B-splines with $\alpha = \gamma - 1$ [31]. These generalized B-splines satisfy the Riesz basis condition for $r = \gamma > 1/2$. The proof is identical to the one given for the symmetric fractional B-splines in [22], which correspond to the special case $\tau = 0$. Moreover, the fractional B-splines are all L_p-stable for $\gamma > 1$. A limiting case is the Haar function with ($\gamma = 1$, $\tau = -1/2$), which is obviously spline admissible as well; this is not so for the other splines of order 1 when τ is not a half-integer. ■

The amplitude response of these operators is $|\hat{L}_{\gamma,\tau}(\omega)| = |\omega|^{\gamma}$, which clearly indicates that they are of order γ and that they correspond to γth fractional derivatives, which will be denoted by ∂_τ^γ.

B. Fractional Derivatives and Test Functions

In general, these fractional derivatives are nonlocal operators unless $\gamma = n$ (integer) and $\tau = -\gamma/2$ (causal version), which corresponds to the usual definition of the derivative. This can lead to some theoretical difficulties and makes it necessary to get an estimate of the type of decay that should be expected when applying them to rapidly decreasing functions.

Theorem 3: Let $\phi(t) \in \mathcal{S}$ (Schwartz' class of functions) be an indefinitely differentiable test function with rapidly decreasing usual derivatives (i.e., faster than polynomial rate). Then, $\partial_\tau^\gamma \phi(t)$ with $\gamma \geq 0$ is indefinitely differentiable and has at least polynomial decay in the sense that

$$|\partial_\tau^\gamma \phi(t)| \leq \frac{\text{Const}}{|1+t|^{\gamma+1}}.$$

Proof: The Fourier transform of a function of $\mathcal{S}$ has fast decay and multiplying it by a polynomial (e.g., the frequency response of the fractional derivative) preserves this property. Using the usual duality between decay and differentiation, we immediately deduce that the fractional derivative of a function of $\mathcal{S}$ is indefinitely differentiable.

Concerning the polynomial decay of this fractional derivative, our reasoning requires three steps.

- First, the exact computation of the fractional derivative of $v(t) = (1+t^2)^{-1}$ (note that $v(t) \notin \mathcal{S}$):

$$\partial_\tau^\gamma \left\{ \frac{1}{1+t^2} \right\} = \Re \frac{e^{j\pi\tau}\Gamma(\gamma+1)}{(1-jt)^{\gamma+1}}.$$

  This result is easily obtained from the Fourier expression of $\partial_\tau^\gamma v$, i.e., $e^{j\pi\tau}\omega^\gamma e^{-\omega}$ for $\omega \geq 0$, and its Hermite conjugate for $\omega < 0$.

- Second, the observation that, if $\phi(t) \in \mathcal{S}$, then the function

$$\psi(t) = \partial_\tau^\gamma \left\{ \phi(t) - \frac{\hat{\phi}(0)}{\pi(1+t^2)} \right\}$$
$$\overset{\text{Fourier}}{\rightsquigarrow} \hat{\psi}(\omega) = (-j\omega)^{\frac{\gamma}{2}-\tau}(j\omega)^{\frac{\gamma}{2}+\tau}\left(\hat{\phi}(\omega) - \hat{\phi}(0)e^{-|\omega|}\right)$$

  decreases at least as Const $\times$ $(1+|t|^{\lceil\gamma\rceil+1})^{-1}$. This is proven by analyzing the Fourier transform of $\psi(t)$, more specifically, by showing that it satisfies $\hat{\psi}^{(n)}(\omega) \in L_1$ for all positive integer $n \leq \lceil\gamma\rceil + 1$. Indeed, $\hat{\psi}(\omega)$ is indefinitely differentiable everywhere, except at $\omega = 0$; moreover, $\hat{\psi}(\omega)$ and its usual derivatives of any order are rapidly decreasing. On the other hand, in the neighborhood of $\omega = 0$, $\hat{\psi}(\omega)$ is $O(|\omega|^{\gamma+1})$, which implies that $\hat{\psi}^{(n)}(\omega)$ is locally L_1 around 0. Put together, this implies that $\hat{\psi}^{(n)}(\omega) \in L_1$. Finally, using the identity

$$(-jt)^n \psi(t) = \frac{1}{2\pi}\int \hat{\psi}^{(n)}(\omega)e^{j\omega t}\mathrm{d}\omega$$

  and the absolute integrability of $\hat{\psi}^{(n)}$, we are able to deduce that $|t|^n|\psi(t)| \leq$ Const for $n = 0, 1, \ldots (\lceil\gamma\rceil + 1)$, which proves the claim of this step.

- Last, the identity

$$\partial_\tau^\gamma \phi(t) = \psi(t) + \frac{\hat{\phi}(0)}{\pi}\partial_\tau^\gamma \left\{ \frac{1}{1+t^2} \right\}$$

  and the results of the previous steps lead to the following bound:

$$|\partial_\tau^\gamma \phi(t)| \leq \frac{\text{Const}}{1+|t|^{\lceil\gamma\rceil+1}} + \frac{\text{Const}}{1+|t|^{\gamma+1}} \leq \frac{\text{Const}'}{1+|t|^{\gamma+1}}.$$

■

In order to extend the fractional differentiation to distributions u, it suffices to observe that for functions ϕ and ψ of $\mathcal{S}$, we have the dual property $\langle \partial_\tau^\gamma \phi, \psi \rangle = \langle \phi, \partial_{-\tau}^\gamma \psi \rangle$. It is then natural to define the scalar product of a distribution u with a test function $\phi(t)$ by $\langle \partial_\tau^\gamma u, \phi \rangle = \langle u, \partial_{-\tau}^\gamma \phi \rangle$. Theorem 3 tells us that we must restrict the admissible distributions to those that admit test functions that are indefinitely differentiable but may decrease as slowly as Const $\times$ $(1+|t|)^{-1}$.

C. Fractional B-Splines

Going back to the introductory example in Section II-A, we notice that the frequency response of the corresponding first-order localization operator Δ_+ is $\Delta_+(e^{j\omega}) = 1 - e^{-j\omega} = j\omega + O(\omega^2)$. Hence, it makes perfect sense to introduce the generalized fractional localization operator by its Fourier transform

$$\Delta_\tau^\gamma(e^{j\omega}) = (1-e^{-j\omega})^{\frac{\gamma}{2}+\tau}(1-e^{j\omega})^{\frac{\gamma}{2}-\tau}$$

which provides a discrete approximation of the fractional derivative ∂_τ^γ. By using a generalized version of the binomial expansion and taking the inverse Fourier transform, it is possible to obtain the exact analytical formulae of the corresponding filter coefficients $d_\tau^\gamma[k]$ in terms of generalized factorials involving the Euler's gamma function [31]. The sequences can also be shown to decrease like $1/|k|^{\gamma+1}$ when $|k| \to \infty$. Once again, it is possible to apply fractional finite differences to distributions by using the duality relation $\langle \Delta_\tau^\gamma u, \varphi \rangle = \langle u, \Delta_{-\tau}^\gamma \varphi \rangle$.

By using the expression of Δ_τ^γ and the definition of the generalized B-splines in Section II-B, we readily obtain the Fourier domain representation of the fractional B-splines of degree[3] $\alpha = \gamma - 1$ and asymmetry parameter τ:

$$\hat{\beta}_\tau^\alpha(\omega) = \frac{\Delta_\tau^{\alpha+1}(e^{j\omega})}{(j\omega)^{\frac{\alpha+1}{2}+\tau}(-j\omega)^{\frac{\alpha+1}{2}-\tau}} = \left(\frac{1-e^{-j\omega}}{j\omega}\right)^{\frac{\alpha+1}{2}+\tau}\left(\frac{1-e^{j\omega}}{-j\omega}\right)^{\frac{\alpha+1}{2}-\tau}. \quad (14)$$

For $\tau = (\alpha+1)/2$, we recover the causal fractional splines $\beta_+^\alpha(t)$ of degree α, which are made of building blocks of the type $t_+^\alpha/\Gamma(\alpha+1)$. These particular functions play a fundamental role in wavelet theory in the sense that every scaling function can be represented as the convolution product between a fractional B-spline and a singular distribution [18].

[3]The terminology "of degree α" is used to signify that the elementary building blocks of these splines are power functions of degree α. On the other hand, their order of approximation is $\gamma = \alpha + 1$, which also coincides with the differential order of the defining operator ∂_τ^γ.

D. Multiresolution Properties

Our definition of scale-invariant L-splines implies that the underlying functions have some fundamental multiresolution properties. Specifically, we have that $s(t) \in \mathcal{V}(\beta_\tau^{\gamma-1}) \Rightarrow s(t/m) \in \mathcal{V}(\beta_\tau^{\gamma-1})$, $\forall m \in \mathbb{N}^+$, which follows directly from the property that $\partial_\tau^\gamma\{s(t/m)\} = \sum_{k\in\mathbb{Z}} m^{1-\gamma} a[k]\delta(t-mk)$, because the defining operator ∂_τ^γ is scale invariant of order γ. This implies that the underlying B-splines must satisfy a general scaling relation

$$\beta_\tau^\alpha(t/m) = \sum_{k\in\mathbb{Z}} g_{\tau,m}^\alpha[k]\beta_\tau^\alpha(t-k) \tag{15}$$

where $g_{\tau,m}^\alpha[k]$ (scaling filter) is an appropriate sequence of weights corresponding to the expansion coefficients of $\beta_\tau^\alpha(t/m)$ in $\mathcal{V}(\beta_\tau^\alpha)$. The Fourier domain equivalent of (15) is $m\hat{\beta}_\tau^\alpha(m\omega) = G_{\tau,m}^\alpha(e^{j\omega})\hat{\beta}_\tau^\alpha(\omega)$. By plugging in the explicit formula (14) for $\hat{\beta}_\tau^\alpha(\omega)$ and solving for the frequency response of the scaling filter, we find that

$$G_{\tau,m}^\alpha(e^{j\omega}) = \frac{1}{m^\alpha}\left(\frac{1-e^{-jm\omega}}{1-e^{-j\omega}}\right)^{\frac{\alpha+1}{2}+\tau}\left(\frac{1-e^{jm\omega}}{1-e^{j\omega}}\right)^{\frac{\alpha+1}{2}-\tau} \tag{16}$$

which is clearly 2π-periodic. The case $m = 2$ in (15) is of special interest because it yields the corresponding two-scale relation, which is central to wavelet theory [15], [18], [32]. We note, however, that the present scaling relation is more general because it holds for any positive integer m, and not just powers of 2.

IV. Fractional Smoothing Splines

Our purpose in this section is to present an in-depth investigation of smoothing spline estimators for the case where the regularization operator is scale invariant as specified in Section III. We will characterize the corresponding fractional smoothing spline estimators and propose an efficient Fourier-based algorithm.

A. Basic Solution

Given a discrete noisy input signal, $\{y[k]\}_{k\in\mathbb{Z}}$, the problem is thus to determine the optimal estimator $s(t)$ such that

$$\min_{s(t)}\left(\sum_{k\in\mathbb{Z}}|v[k]*(y[k]-s(k))|^2 + \lambda\,\|\partial_\tau^\gamma s\|_{L_2}^2\right) \tag{17}$$

where $v[k]$ is a suitable positive-definite weighting sequence.

We have just seen that ∂_τ^γ is a scale-invariant, spline-admissible operator of order γ corresponding to the spline generator $\beta_\tau^{\gamma-1}(t)$. We can therefore apply Theorem 2, which tells us that the optimal solution is a fractional spline specified by (11) with $\varphi(t) = \beta_\tau^{\gamma-1}(t) * \beta_\tau^{\gamma-1}(-t)$. By using (14), we obtain the Fourier transform of the optimal generator

$$\hat{\varphi}(\omega) = \left|\hat{\beta}_\tau^{\gamma-1}(\omega)\right|^2 = \left|\frac{\sin(\omega/2)}{\omega/2}\right|^{2\gamma} = \hat{\beta}_0^{2\gamma-1}(\omega) \tag{18}$$

which does not depend on τ anymore. As indicated by the right-hand side of (18), this corresponds to a fractional B-spline of order 2γ and asymmetry parameter $\tau = 0$. It is also equivalent to the symmetric B-spline $\beta_*^\alpha(t)$ of degree $\alpha = 2\gamma-1$, which is fully characterized in [22].

The localization operator for $\varphi(t) = \beta_0^{2\gamma-1}(t)$ can be seen to be $\Delta_0^{2\gamma}$ whose Fourier transform is $\Delta_0^{2\gamma}(e^{j\omega}) = |2\sin(\omega/2)|^{2\gamma}$. Likewise, we can use Poisson's summation formula to compute the Fourier transform of the sampled version of the symmetric fractional B-spline, as follows:

$$A_\gamma(e^{j\omega}) = \sum_{k\in\mathbb{Z}}\beta_0^{2\gamma-1}(k)e^{-j\omega k} = \sum_{n\in\mathbb{Z}}\hat{\beta}_0^{2\gamma-1}(\omega+2\pi n). \tag{19}$$

Finally, by substituting (18) into the right-hand side of (19), we get an explicit formula for the smoothing spline filter (10) associated with the fractional differentiation operator ∂_τ^γ, as follows:

$$H(e^{j\omega}) = \frac{1}{\sum_{n\in\mathbb{Z}}\left|\frac{2\sin(\omega/2)}{\omega+2\pi n}\right|^{2\gamma} + \lambda\frac{|2\sin(\omega/2)|^{2\gamma}}{|V(e^{j\omega})|^2}}. \tag{20}$$

B. Characterization of Smoothing Spline Estimators

We now consider the special case where the weighting sequence v in (17) is the identity (i.e., $V(e^{j\omega}) = 1$). To characterize the underlying estimator, we rewrite the smoothing spline solution as

$$s(t) = \sum_{k\in\mathbb{Z}} y[k]\varphi_{\gamma,\lambda}(t-k)$$

where $y[k]$ is the (noisy) input sequence and where $\varphi_{\gamma,\lambda}(t) = \sum_{k\in\mathbb{Z}} h[k]\beta_0^{2\gamma-1}(t-k)$ is an equivalent spline basis function that represents the impulse response of the smoothing spline algorithm. Here, we have made use of the commutativity of the convolution operation and have moved the digital reconstruction h in (11) on the side of the basis functions, instead of applying it to the digital input signal. Using (20) and (18), we obtain the frequency response of the smoothing spline estimator as

$$\begin{aligned}\hat{\varphi}_{\gamma,\lambda}(\omega) &= H(e^{j\omega})\cdot\hat{\beta}_0^{2\gamma-1}(\omega)\\ &= \frac{\left|\frac{2\sin(\omega/2)}{\omega}\right|^{2\gamma}}{\sum_{n\in\mathbb{Z}}\left|\frac{2\sin(\omega/2)}{\omega+2\pi n}\right|^{2\gamma} + \lambda\,|2\sin(\omega/2)|^{2\gamma}}\\ &= \frac{1}{\sum_{n\in\mathbb{Z}}\left|\frac{\omega}{\omega+2\pi n}\right|^{2\gamma} + \lambda|\omega|^{2\gamma}}.\end{aligned} \tag{21}$$

Next, we show that this operator essentially behaves like a classical Butterworth filter of order 2γ and cutoff frequency ω_0, which is defined as (cf. [33] and [34])

$$B_{2\gamma}\left(\frac{\omega}{\omega_0}\right) := \frac{1}{1+\left|\frac{\omega}{\omega_0}\right|^{2\gamma}}. \tag{22}$$

While γ is traditionally constrained to be an integer, this definition is applicable for $\gamma \in \mathbb{R}^+$ as well.

Theorem 4: The frequency response $\hat{\varphi}_{\gamma,\lambda}(\omega)$ of the smoothing spline estimator of order 2γ and regularization parameter $\lambda \geq 0$ satisfies the following inequalities.

1) For $-\pi \leq \omega \leq \pi$

$$B_{2\gamma}\left(\frac{\omega}{\omega_{\min}}\right) \leq \hat{\varphi}_{\gamma,\lambda}(\omega) \leq B_{2\gamma}\left(\frac{\omega}{\omega_{\max}}\right) \leq 1 \quad (23)$$

with

$$\omega_{\min} = \left(\lambda + \frac{2\zeta(2\gamma)(1-2^{-2\gamma})-1}{\pi^{2\gamma}}\right)^{-\frac{1}{2\gamma}}$$

$$\omega_{\max} = \left(\lambda + \frac{2\zeta(2\gamma)}{(2\pi)^{2\gamma}}\right)^{-\frac{1}{2\gamma}}$$

where $\zeta(s) = \sum_{n=1}^{\infty} n^{-s}$ is the Riemann zeta function.

2) For $|\omega| \geq \pi$

$$|\hat{\varphi}_{\gamma,\lambda}(\omega)| \leq B_{2\gamma}\left(\frac{\omega}{\omega_0}\right) = O\left(|\omega|^{-2\gamma}\right) \quad (24)$$

with

$$\omega_0 = \left(\lambda + \frac{1}{\pi^{2\gamma}}\right)^{-\frac{1}{2\gamma}} \leq \pi. \quad (25)$$

Proof: We rewrite (21) as

$$\hat{\varphi}_{\gamma,\lambda}(\omega) = \frac{1}{1+|\omega|^{2\gamma}\left(\lambda + \frac{F\left(\frac{\omega}{2\pi}\right)}{(2\pi)^{2\gamma}}\right)} \quad (26)$$

where $F(x)$ is the auxiliary function defined by

$$F(x) = \sum_{n=1}^{\infty}\left(\frac{1}{|x+n|^{2\gamma}} + \frac{1}{|-x+n|^{2\gamma}}\right). \quad (27)$$

Clearly, $F(x)$ is symmetric and positive. Moreover, we can show that it is monotonically increasing for $0 \leq x \leq (1/2)$. Hence, we have the following bound:

$$\forall\omega \in [-\pi,\pi], \quad F(0) \leq F\left(\frac{\omega}{2\pi}\right) \leq F\left(\frac{1}{2}\right).$$

Next, we note that $F(0) = 2\zeta(2\gamma)$ and use the identity $\sum_{n\in\mathbb{Z}}|2n-1|^{-s} = 2\zeta(s)(1-2^{-s})$ (cf. [35, Sec. 23.26]) to show that $F(1/2) = 2\zeta(2\gamma)(2^{2\gamma}-1) - 2^{2\gamma}$, which yields the first part of the theorem.

For the second part, we note that the dominant term in (27) for $x = \omega/2\pi$ with $|x| \geq (1/2)$ corresponds to the index $n_0 = \mathrm{round}(x)$. Denoting $\Delta x_0 = |(\omega/2\pi) - n_0|$, we therefore have

$$\forall|\omega| \geq \pi,\ F\left(\frac{\omega}{2\pi}\right) > \frac{1}{(\Delta x_0)^{2\gamma}} \geq 2^{2\gamma}$$

simply because $\Delta x_0 \leq (1/2)$. Using the definition of ω_0 in (25) and making use of the above inequality in (26), we ultimately get

$$\hat{\varphi}_{\gamma,\lambda}(\omega) \leq \frac{1}{1+\left|\frac{\omega}{\omega_0}\right|^{2\gamma}}$$

which holds for $|\omega| \geq \pi$. ∎

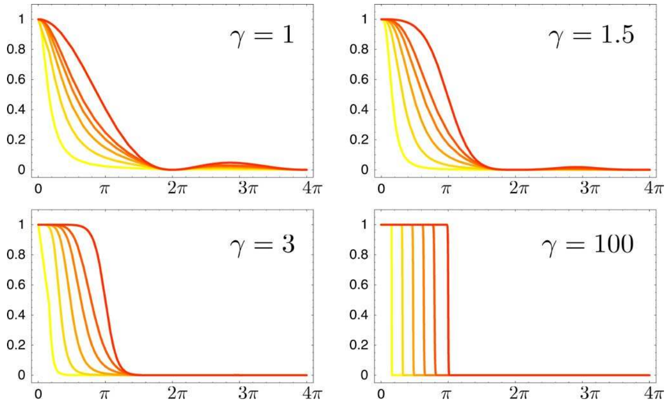

Fig. 2. Frequency responses of smoothing spline estimators as λ and γ varies. Each subplot corresponds to a fixed value of γ and the filters of a given color are matched according to their equivalent bandwitdh: $\omega_0 = \pi, 4\pi/5, 3\pi/5, 2\pi/5$, and $\pi/5$.

Since $\omega_{\min} \leq \omega_0 \leq \omega_{\max}$, the interpretation of Theorem 4 is that the frequency response of the smoothing spline filter with regularization parameter λ closely matches that of a Butterworth filter of fractional order 2γ and cutoff frequency given by (25). Conversely, we may specify an equivalent bandwitdth $\omega_0 \leq \pi$ and select the regularization parameter accordingly (cf. (25)), as follows:

$$\lambda = \omega_0^{-2\gamma} - \pi^{-2\gamma}.$$

The variety of responses that can be obtained by varying γ and λ is illustrated in Fig. 2. In these examples, the latter parameter was computed using the above equation with $\omega_0 = \pi, 4\pi/5, 3\pi/5, 2\pi/5$, and $\pi/5$. The behavior of these filters is clearly lowpass with a response that gets sharper and closer to the ideal one as γ increases.

We note that the Butterworth approximation $\hat{\varphi}_{\gamma,\lambda}(\omega) \approx B_{2\gamma}(\omega/\omega_0)$ improves as λ increases, in which case the cutoff frequencies $\omega_{\min}, \omega_0$, and $\omega_{\max}$ get closer to $1/\sqrt[2\gamma]{\lambda}$. The same type of effect can also be observed as γ gets larger; indeed, $\omega_{\min}$ rapidly converges to ω_0 with the consequence that the lower bound in (23) becomes undistinguishable from the upper bound in (24).

For the particular case $\lambda = 0$, the estimator is equivalent to a spline interpolator of degree $2\gamma - 1$. The corresponding cutoff frequency is $\omega_0 = \pi$ (Nyquist frequency). As γ increases, the frequency response converges to an ideal filter as illustrated in Fig. 3; this is consistent with earlier findings for the integer case [36].

While the above results suggest a close connection between smoothing spline estimators and Butterworth filters, we also like to point out two fundamental differences. The first is the context: in the present case, the input of the spline estimator is discrete; this is in contrast with traditional Butterworth filters which are designed for processing analog signals. The second difference concerns the reproduction of polynomials, which is a property that is specific to splines.

Proposition 3: A smoothing spline estimator of order 2γ has the ability to reproduce the polynomials of degree $n = \lceil 2\gamma -$

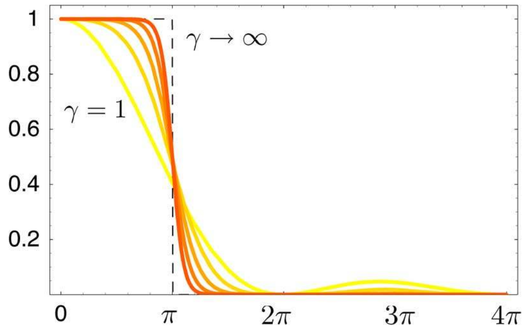

Fig. 3. Frequency response $\hat{\varphi}_{\gamma,0}(\omega)$ of fractional spline interpolators for increasing values of γ. In the limit, $\hat{\varphi}_{\gamma,0}(\omega)$ tends to the ideal filter $\mathrm{rect}(\omega/(2\pi))$.

$1\rceil$, irrespective of the value of the regularization parameter λ. Specifically, we have that

$$t^m = \sum_{k\in\mathbb{Z}} k^m \varphi_{\gamma,\lambda}(t-k), \quad \text{for } m = 0, \cdots, n.$$

Proof: By using Poisson's summation formula, one gets an equivalent relation in the Fourier domain; the so-called Strang–Fix conditions of order $n-1$

$$\hat{\varphi}^{(m)}_{\gamma,\lambda}(2\pi k) = \delta[m]\delta[k] \text{ for } k \in \mathbb{Z} \text{ and } m = 0, \cdots, n$$

where $\delta[k]$ is the Kronecker impulse and where $\varphi^{(m)}_{\gamma,\lambda}$ denotes the mth derivative of the Fourier transform of $\varphi_{\gamma,\lambda}$. The condition $\hat{\varphi}^{(m)}_{\gamma,\lambda}(0) = \delta[m]$ follows for the 2γth-order flatness of $\hat{\varphi}_{\gamma,\lambda}(\omega)$ around the origin; indeed, a simple Taylor series development of (26) yields the asymptotic relation $\hat{\varphi}_{\gamma,\lambda}(\omega) = 1 - (\lambda + 2\zeta(2\gamma)/(2\pi)^{2\gamma})|\omega|^{2\gamma}$ as $\omega \to 0$. Otherwise, $\hat{\varphi}^{(m)}_{\gamma,\lambda}$ has the required vanishing properties because the smoothing spline filter contains a fractional B-spline factor that imposes zeros of multiplicity 2γ at $\omega = 2\pi k$, $k \in \mathbb{Z} \setminus \{0\}$ (cf. [22, Sec. 4.1]). ■

This means that the smoothing spline estimator is a *quasi-interpolant* of degree $\lceil 2\gamma - 1\rceil$, which is the maximum possible within the given spline space [22], [37], [38]. While we would expect a perfect reconstruction of any polynomial in the null space of the regularizing operator ∂^{γ}_{τ}—i.e., with a degree less or equal to $\lceil \gamma - 1\rceil$—it comes as a nice surprise to see that the property extends to twice the order.

C. Fast Fractional Smoothing Splines

In our earlier work, we have presented an efficient recursive algorithm for computing linear and cubic smoothing splines, i.e., $\gamma = 1, 2$ [39]. For the more general fractional case where γ is not necessarily integer, we propose an alternative approach that uses a combination of Fourier and multirate filtering techniques.

In practice, one is often more interested in the samples of the smoothing spline $s(t)$ than in the B-spline coefficients per se. The integer samples of the solution can be computed efficiently by applying a postfilter that corresponds to the sampled version of the B-spline generator $\beta_0^{2\gamma-1}(t)$. A similar technique is applicable for evaluating a finer version of the solution with an

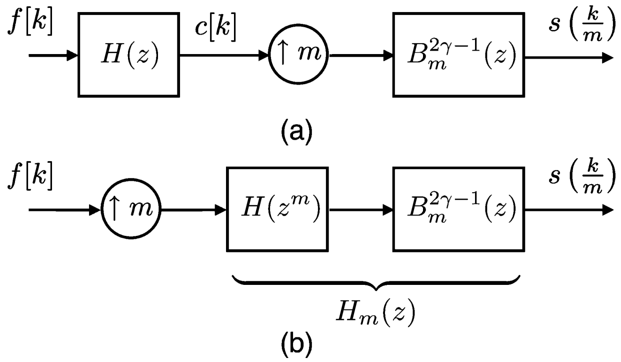

Fig. 4. Equivalent multirate filtering algorithms for the implementation of smoothing splines.

oversampling factor of m (integer). The corresponding block diagram is given in Fig. 4(a), where the first filter $H(z)$ provides the B-spline coefficients $c[k]$ and where $B_m^{2\gamma-1}(z)$ corresponds to the oversampling of the basis functions by a factor of m. We can also move the smoothing spline filter $H(z)$ to the right-hand side of the upsampling operator and combine the two filters into a single one whose equivalent z-transform is $H_m(z) = H(z^m)\cdot B_m^{2\gamma-1}(z)$, as illustrated in Fig. 4(b). Further, by using the scaling relation (15), we show that

$$\begin{aligned} B_m^{2\gamma-1}(e^{j\omega}) &= \sum_{k\in\mathbb{Z}} \beta_0^{2\gamma-1}(k/m)e^{-j\omega k} \\ &= \left(\frac{1}{m^{2\gamma-1}}\left|\frac{\sin(m\omega/2)}{\sin(\omega/2)}\right|^{2\gamma}\right) A_\gamma(e^{j\omega}) \end{aligned}$$

where the central factor corresponds to the scaling filter $G_{0,m}^{2\gamma-1}(e^{j\omega})$ as specified by (16). Finally, by combining these various formulas, we obtain the frequency response of the equivalent digital smoothing spline filter in Fig. 4(b), as follows:

$$\begin{aligned} H_m(e^{j\omega}) = \frac{1}{m^{2\gamma-1}}&\left|\frac{\sin(m\omega/2)}{\sin(\omega/2)}\right|^{2\gamma} \\ &\times \frac{A_\gamma(e^{j\omega})}{A_\gamma(e^{jm\omega}) + \lambda\frac{|2\sin(m\omega/2)|^{2\gamma}}{|V(e^{jm\omega})|^2}} \end{aligned} \quad (28)$$

where $A_\gamma(e^{j\omega})$ is defined by (19). The last ingredient that we need is an efficient way to evaluate $A_\gamma(e^{j\omega}) = B_1^{2\gamma-1}(e^{j\omega})$ for any given value of ω. This can be done by way of the following accelerated partial sum formula:

$$\begin{aligned} A_\gamma(e^{j\omega}) \approx &\left[\sum_{n=-M}^{M}\left|\frac{2\sin(\omega/2)}{\omega+2\pi n}\right|^{2\gamma}\right] + \left|\frac{\sin(\omega/2)}{\pi M}\right|^{2\gamma} \\ &\times\left[\frac{2M}{2\gamma-1} - 1 + \frac{\gamma\left(\frac{2}{3}\pi^2+\omega^2\right)}{2\pi^2 M} - \frac{\gamma(2\gamma+1)\omega^2}{4\pi^2M^2}\right] \end{aligned} \quad (29)$$

which has a remainder that is $O(M^{-2\gamma-3})$, as compared with $O(M^{-2\gamma+1})$ for a partial sum without the correction term. Practically, this means that we can evaluate $A_\gamma(e^{j\omega})$ to machine pre-

cision using (29) with a reasonably small number of terms, say $M = 10$.

We now have all the elements to describe our fast fractional smoothing spline algorithm whose complexity is essentially that of the FFT.

1) Computation of the N-point FFT of the input signal $\{y[k]\}_{k=0,\cdots,N-1}$: this yields the Fourier coefficients $\{Y[n]\}_{n=0,\cdots,N-1}$.
2) Fourier domain implementation of the upsampling by m: this is achieved by extending $Y[n]$ to a sequence of length mN using N-periodic boundary conditions.
3) Filtering by multiplication in the Fourier domain: the sampled frequency response of the digital smoothing spline filter is evaluated using (28) and (29) with $\omega = n2\pi/mN$, for $n = 0, \cdots, mN-1$.
4) Evaluation of $\{s(k/m)\}_{k=0,\cdots,mN-1}$ by mN-point inverse FFT.

This algorithm has been coded in Matlab and is available from the authors upon request.

V. Conclusion

Starting from first principles—in particular, the notion of self-similarity—we pursued the task of specifying an extended class of scale-invariant L-splines together with some efficient signal processing algorithms for signal interpolation and approximation.

Our starting point was the identification of the family of differential operators that are both shift and scale invariant (i.e., L is such that it (pseudo)commutes with shifts and dilations); these are the generalized fractional derivatives ∂_τ^γ, which are indexed by an order parameter $\gamma > (1/2)$ and an asymmetry factor τ. The corresponding fractional splines are conveniently represented as a linear combination of fractional B-splines, which are localized versions of the Green functions of the defining operator ∂_τ^γ.

Using the operator ∂_τ^γ, we also introduced a spline energy $\|\partial_\tau^\gamma s\|_{L_2}^2$ that could be used as a regularization functional for the stable reconstruction of continuous-time functions from discrete measurements. Interestingly, the optimal solutions are all fractional splines of order 2γ (or, degree $\alpha = 2\gamma - 1$) and are essentially scale invariant. Specifically, if we relocate the samples of a signal on a grid dilated by a factor of T and determine the interpolation function $s(t)$ that minimizes $\|\partial_\tau^\gamma s\|_{L_2}^2$, we obtain a fractional spline solution that is precisely the dilated version of the solution for $T = 1$. We can also achieve the same in the smoothing spline case via an appropriate rescaling of λ. This means that the spline fitting process commutes with the rescaling of the time axis, which is a reasonable requirement if one is looking for a universal algorithm that does not depend on a particular choice of units or reference system. Of course, this is a feature that is specific to fractional splines and that takes its roots in the scale invariance of the defining operator L. Another interesting consequence of the scale invariance of the operator, as well as of the underlying Green function, is that the fractional B-splines all satisfy a two-scale relation [16]—this means that they can be used as elementary building blocks for the construction of (fractional) wavelet bases of L_2 [18].

An important aspect of our investigation has been the characterization of fractional smoothing spline estimators that are optimal in a deterministic, variational sense. We have shown how these could be implemented efficiently by means of FFTs. We have specified the underlying filters and have uncovered an interesting connection with the classical Butterworth filters. While we did investigate the influence of the order 2γ and the regularization parameter λ on the filter characteristics, we did not yet provide general guidelines as to how these should be adjusted in practice for best performance. We will now show that we can obtain a satisfactory answer to this question by adopting a stochastic formulation of the spline estimation problem. This will take us to the next step which is the unraveling of the connection between splines and fractals [40].

References

[1] B. B. Mandelbrot, *The Fractal Geometry of Nature*. San Francisco, CA: Freeman, 1982.

[2] H. O. Peitgen and D. Saupe, Eds., *The Science of Fractal Images*. New York: Springer-Verlag, 1988.

[3] B. B. Mandelbrot and J. W. Van Ness, "Fractional Brownian motions fractional noises and applications," *SIAM Rev.*, vol. 10, no. 4, pp. 422–437, 1968.

[4] B. Pesquet-Popescu and J. Lévy Véhel, "Stochastic fractal models for image processing," *IEEE Signal Process. Mag.*, vol. 19, no. 5, pp. 48–62, 2002.

[5] A. M. Yaglom, "Correlation theory of processes with random stationary n-th increments," *Amer. Math. Soc. Translations*, ser. 2, vol. 2, no. 8, pp. 87–141, 1958.

[6] M. S. Taqqu, "Fractional Brownian motion and long-range dependence," in *Theory and Applications of Long-Range Dependence*, P. Doukhan, G. Oppenheim, and M. S. Taqqu, Eds. Boston, MA: Birkhauser, 2003, pp. 5–38.

[7] A. M. Yaglom, *Correlation Theory of Stationary and Related Random Functions I: Basic Results*. New York: Springer, 1986.

[8] P. Flandrin, "Wavelet analysis and synthesis of fractional Brownian-motion," *IEEE Trans. Inf. Theory*, vol. 38, no. 2, pp. 910–917, 1992.

[9] J. Lamperti, "Semi-stable stochastic processes," *Trans. Amer. Math. Soc.*, vol. 1094, pp. 62–78, 1962.

[10] G. W. Wornell, "Wavelet-based representations for the $1/f$ family of fractal processes," *Proc. IEEE*, vol. 81, no. 10, pp. 1428–1450, 1993.

[11] B. S. Chen and C. W. Lin, "Multiscale Wiener filter for the restoration of fractal signals: Wavelet filter bank approach," *IEEE Trans. Signal Process.*, vol. 42, no. 11, pp. 2972–2982, Nov. 1994.

[12] G. A. Hirchoren and C. E. D'Attellis, "Estimation of fractional Brownian motion with multiresolution Kalman filter banks," *IEEE Trans. Signal Process.*, vol. 47, no. 5, pp. 1431–1434, May 1999.

[13] G. W. Wornell, "A Karhunen–Loève-like expansion for $1/f$ processes via wavelets," *IEEE Trans. Inf. Theory*, vol. 36, no. 4, pp. 859–861, 1990.

[14] S. G. Mallat, "A theory of multiresolution signal decomposition: the wavelet representation," *IEEE Trans. Pattern Anal. Mach. Intell.*, vol. 11, no. 7, pp. 674–693, 1989.

[15] S. Mallat, *A Wavelet Tour of Signal Processing*. San Diego, CA: Academic, 1998.

[16] T. Blu and M. Unser, "Wavelets, fractals, and radial basis functions," *IEEE Trans. Signal Process.*, vol. 50, no. 3, pp. 543–553, Mar. 2002.

[17] C. A. Cabrelli, C. Heil, and U. M. Molter, "Self-similarity and multiwavelets in higher dimensions," *Mem. Amer. Math. Soc.*, vol. 170, no. 807, 2004.

[18] M. Unser and T. Blu, "Wavelet theory demystified," *IEEE Trans. Signal Process.*, vol. 51, no. 2, pp. 470–483, Feb. 2003.

[19] I. J. Schoenberg, "Contribution to the problem of approximation of equidistant data by analytic functions," *Quart. Appl. Math.*, vol. 4, pp. 45–99, 1946, 112-141.

[20] M. Unser, "Splines: A perfect fit for signal and image processing," *IEEE Signal Process. Mag.*, vol. 16, no. 6, pp. 22–38, 1999.

[21] ——, "Cardinal exponential splines: Part II—Think analog, act digital," *IEEE Trans. Signal Process.*, vol. 53, no. 4, pp. 1439–1449, Apr. 2005.

[22] M. Unser and T. Blu, "Fractional splines and wavelets," *SIAM Rev.*, vol. 42, no. 1, pp. 43–67, 2000.

[23] ——, "Generalized smoothing splines and the optimal discretization of the Wiener filter," *IEEE Trans. Signal Process.*, vol. 53, no. 6, pp. 2146–2159, Jun. 2005.
[24] A. Aldroubi and K. Gröchenig, "Nonuniform sampling and reconstruction in shift invariant spaces," *SIAM Rev.*, vol. 43, pp. 585–620, 2001.
[25] I. J. Schoenberg, "Cardinal interpolation and spline functions," *J. Approx. Theory*, vol. 2, pp. 167–206, 1969.
[26] ——, "Spline functions and the problem of graduation," *Proc. Nat. Acad. Sci.*, vol. 52, pp. 947–950, 1964.
[27] C. H. Reinsh, "Smoothing by spline functions," *Numer. Math.*, vol. 10, pp. 177–183, 1967.
[28] K. Gröchenig, *Foundations of Time-Frequency Analysis*. Cambridge, MA: Birkhäuser, 2001.
[29] L. Schwartz, *Théorie des Distributions*. Paris, France: Hermann, 1966.
[30] I. M. Gelfand and G. Shilov, *Generalized Functions*. New York: Academic, 1964, vol. 1.
[31] T. Blu and M. Unser, "A complete family of scaling functions: The (α, τ)-fractional splines," in *Proc. 28th Int. Conf. Acoustics, Speech, Signal Processing (ICASSP'03)*, Hong Kong S.A.R., Apr. 6–10, 2003, vol. VI, pp. 421–424.
[32] I. Daubechies, *Ten Lectures on Wavelets*. Philadelphia, PA: Society for Industrial and Applied Mathematics, 1992.
[33] S. Butterworth, "On the theory of filter amplifiers," *Wireless Eng.*, vol. 7, pp. 536–541, 1930.
[34] V. D. Landon, "Cascade amplifiers with maximal flatness," *RCA Rev.*, vol. 5, pp. 347–362, 1941.
[35] M. Abramowitz and I. A. Stegun, *Handbook of Mathematical Functions*. Washington, DC: National Bureau of Standards, 1972.
[36] A. Aldroubi, M. Unser, and M. Eden, "Cardinal spline filters: Stability and convergence to the ideal sinc interpolator," *Signal Process.*, vol. 28, no. 2, pp. 127–138, 1992.
[37] C. de Boor and G. Fix, "Spline approximation by quasi-interpolants," *J. Approx. Theory*, vol. 8, pp. 19–45, 1973.
[38] W. A. Light and E. W. Cheney, "Quasi-interpolation with translates of a function having non-compact support," *Construct. Approx.*, vol. 8, pp. 35–48, 1992.
[39] M. Unser, A. Aldroubi, and M. Eden, "B-spline signal processing: Part II—Efficient design and applications," *IEEE Trans. Signal Process.*, vol. 41, no. 2, pp. 834–848, Feb. 1993.
[40] T. Blu and M. Unser, "Self-similarity: Part II—Optimal estimation of fractal-like processes," *IEEE Trans. Signal Process.*, vol. 55, no. 4, pp. 1364–1378, Apr. 2007.

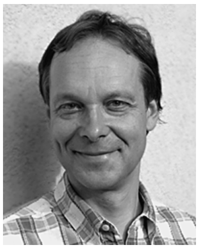

Michael Unser (M'89–SM'94–F'99) received the M.S. (*summa cum laude*) and Ph.D. degrees in electrical engineering from the École Polytechnique Fédérale de Lausanne (EPFL), Switzerland, in 1981 and 1984, respectively.

From 1985 to 1997, he worked as a Scientist with the National Institutes of Health, Bethesda, MD. Currently, he is a Professor and Director of the Biomedical Imaging Group at the EPFL. His primary research interests are biomedical image processing, splines, and wavelets. He is the author of over 140 published journal papers in these areas.

Dr. Unser has been actively involved with the IEEE TRANSACTIONS ON MEDICAL IMAGING, holding the positions of Associate Editor (1999 to 2002 and 2006 to present), Member of the Steering Committee, and Associate Editor-in-Chief (2003 to 2005). He has served as Associate Editor or Member of the Editorial Board for eight more international journals, including the *IEEE Signal Processing Magazine*, the IEEE TRANSACTIONS ON IMAGE PROCESSING (1992 to 1995), and the IEEE SIGNAL PROCESSING LETTERS (1994 to 1998). He organized the first IEEE International Symposium on Biomedical Imaging (ISBI'2002). He currently chairs the technical committee of the IEEE Signal Processing (IEEE-SP) Society on Bio Imaging and Signal Processing (BISP), and well as the ISBI steering committee. He is the recipient of three Best Paper Awards from the IEEE Signal Processing Society.

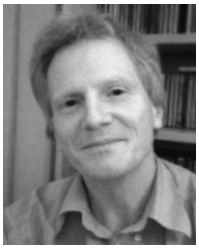

Thierry Blu (M'96–SM'06) was born in Orléans, France, in 1964. He received the "Diplôme d'Ingénieur" from École Polytechnique, France, in 1986 and the Ph.D. degree in electrical engineering from Télécom Paris (ENST), France, in 1988 and 1996, respectively. His Ph.D. focused on a study on iterated rational filter banks, applied to wideband audio coding.

He is with the Biomedical Imaging Group at the Swiss Federal Institute of Technology (EPFL), Lausanne, Switzerland, on leave from France Télécom National Center for Telecommunications Studies (CNET), Issy-les-Moulineaux, France. His research interests include (multi)wavelets, multiresolution analysis, multirate filterbanks, approximation and sampling theory, psychoacoustics, optics, and wave propagation.

Dr. Blu is the recipient of the 2003 best paper award (SP Theory and Methods) from the IEEE Signal Processing Society. He is currently serving as an Associate Editor for the IEEE TRANSACTIONS ON SIGNAL PROCESSING.
