# Supplementary material for: Rhythms and Background (RnB): The Spectroscopy of Sleep Recordings
Source: eNeuro. 2026 Feb 3;13(2):ENEURO.0235-25.2025. doi: 10.1523/ENEURO.0235-25.2025 (PMC12867552; doi:10.1523/ENEURO.0235-25.2025)
Supplement: Data 1 — Download Data 1, ZIP file. [file eneuro-13-ENEURO.0235-25.2025-s002.zip › RnB-Wavelet-main/wRnBmain/rnb_wavelet/rnb/FracSpline/references/unser0403.pdf]

FRACTIONAL SPLINES, WAVELET BASES, AND APPLICATIONS

Michael Unser

Biomedical Imaging Group,
Swiss Federal Institute of Technology Lausanne (EPFL),
CH-1015 Lausanne, Switzerland

Abstract: The purpose of this presentation is to describe a recent family of basis functions—the fractional B-splines—which appear to be intimately connected to fractional calculus. Among other properties, we show that they are the convolution kernels that link the discrete (finite differences) and continuous (derivatives) fractional differentiation operators. We also provide simple closed forms for the fractional derivatives of these splines. The fractional B-splines satisfy a fundamental two-scale relation. Consequently, they can be used as building blocks for constructing a variety of orthogonal and semi-orthogonal wavelet bases of L_2; these are indexed by a continuous order parameter $\gamma = \alpha + 1$, where α is the (fractional) degree of the spline. We show that the corresponding wavelets behave like multiscale differentiation operators of fractional order γ. This is in contrast with classical wavelets whose differentiation order is constrained to be an integer. We also briefly discuss some recent applications in medical and seismic imaging.

Keywords: Splines, wavelets, fractional differentiation, fractional calculus

1. INTRODUCTION

The fractional splines are a recent extension of the polynomial splines for all fractional degrees $\alpha > -1$ (Unser and Blu, 2000). Their basic constituents are piecewise power functions of degree α. One constructs the corresponding B-splines through a localization process similar to the classical one, replacing finite differences by fractional differences (see, Section 2). The fractional B-splines share virtually all the properties of the classical B-splines, including the two-scale relation, and can therefore be used to define new wavelet bases with a continuously-varying order parameter (Unser and Blu, 1999). They only lack positivity and compact support. The fractional splines have the following remarkable properties:

- Generalization: For α integer, they are equivalent to the classical polynomial splines (Schoenberg, 1946). In some sense, the fractional B-splines interpolate the polynomial ones.
- Regularity: The fractional splines are α-Hölder continuous; their critical Sobolev exponent is $\alpha + 1/2$.
- Decay: The fractional B-splines decay at least like $|t|^{\alpha-2}$; they are compactly supported for α integer.
- Order of approximation: The fractional splines have a fractional order of approximation $\alpha + 1$; a property that has not been encountered before in wavelet theory.
- Vanishing moments: The fractional spline wavelets have $\lceil \alpha + 1 \rceil$ vanishing moments, while the fractional B-splines reproduce the polynomials of degree $\lceil \alpha \rceil$.
- Fractional derivatives: Simple formulae are available for obtaining the fractional deriva-

tives of B-splines. In addition, fractional spline wavelets essentially behave like multiscale fractional derivative operators.

- Stretching the bounds of wavelet theory: for $-1/2 < \alpha < 0$, the fractional B-splines do not have the standard regularity factor $(1+z)$. Yet, they yield perfectly valid wavelet bases with some unusual characteristics (order of approximation lesser than 1, singularities at the integers, etc...).

Despite their non-conventional properties and lack of compact support, the fractional spline wavelets are perfectly implementable. The most efficient computational solution is to implement the wavelet filtering in the Fourier domain using the FFT (Fast Fourier transform). Generic Matlab and JAVA software is available for computing the various brands of semi-orthogonal fractional wavelet transforms in one and two dimensions (Blu and Unser, 2000). The parameter $\alpha > -\frac{1}{2}$ can be adjusted in a continuous fashion. These wavelet transforms are non-redundant and fully reversible. Moreover, their orthogonal version constitutes the natural generalization of the Battle-Lemarié wavelet transform (Battle, 1987) which is recovered for α integer.

2. FRACTIONAL SPLINES AND FRACTIONAL DIFFERENTIATION

2.1 Fractional differential operators

Here, we consider the fractional differentiation operator ∂^γ of order $\gamma \in \mathbb{R}^+$, which is most conveniently characterized in the Fourier domain:

$$\mathcal{F}\{\partial^\gamma f(t)\}(\omega) = (j\omega)^\gamma \hat{f}(\omega),$$

where $j = \sqrt{-1}$ and $\hat{f}(\omega) = \mathcal{F}\{f\}$ denotes the Fourier transform of f. If $f(t) \in L_1$ then $\mathcal{F}\{f\}(\omega) = \int_{-\infty}^{+\infty} f(t)e^{-j\omega t}dt$; otherwise, $\mathcal{F}\{f\}$ is defined in the distributional sense.

The discrete version of this operator is the causal fractional difference operator, Δ_+^γ, whose Fourier definition is

$$\mathcal{F}\{\Delta_+^\gamma f(t)\}(\omega) = (1 - e^{-j\omega})^\gamma \hat{f}(\omega), \qquad (1)$$

where we note that $(1 - e^{-j\omega})^\gamma = (j\omega)^\gamma +$ higher-order terms. By applying the generalized binomial expansion to $(1-e^{-j\omega})^\gamma$ and by taking the inverse Fourier transform of (1), we find that:

$$\Delta_+^\gamma f(t) = \sum_{k=0}^{+\infty} (-1)^k \binom{\gamma}{k} f(t-k).$$

The generalized binomial coefficients are given by

$$\binom{u}{v} = \frac{\Gamma(u+1)}{\Gamma(v+1)\Gamma(u-v+1)}$$

where $\Gamma(u)$ is Euler's celebrated gamma function.

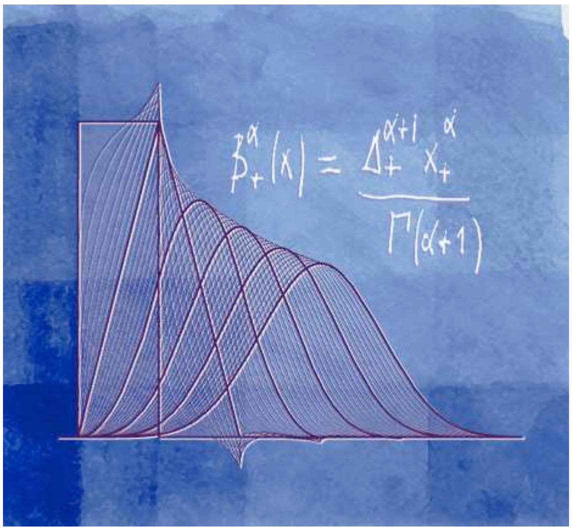

Fig. 1. Artistic rendition of the causal fractional B-splines of degree $\alpha \geq 0$ (authors: Annette Unser and Michael Unser). These functions interpolate the conventional B-splines, represented using thicker lines, in very much the same way as the gamma function (included in the definition) interpolates the factorials.

2.2 Fractional B-splines

The causal fractional B-spline of order $\gamma = \alpha+1$ is most conveniently defined in the Fourier domain by taking the ratio of the frequency responses of the operators Δ_+^γ and ∂_+^γ. Specifically, we have that

$$\mathcal{F}\{\beta_+^\alpha(t)\}(\omega) = \left(\frac{1 - e^{-j\omega}}{j\omega}\right)^{\alpha+1}. \qquad (2)$$

A direct implication of this method of construction is the distributional relation

$$\Delta_+^\gamma f(t) = \left(\beta_+^{\gamma-1} * \partial_+^\gamma f\right)(t),$$

which relates the discrete and continuous fractional differential operators introduced above. In fact, we have learned recently that Zheludev (1983) in Russia had identified the fractional B-splines many years before us while trying to characterize the convergence properties of a discrete, finite-difference-based algorithm for solving Abel's fractional integral equation (which amounts to the calculation of a fractional derivative). However, he did not consider them as generating functions for constructing fractional splines. Because of this fundamental connection with fractional differentiation, it is also quite likely that the fractional B-splines may have been (re-)discovered independently by other researchers having a direct interest in this type of operators.

The last step in our spline construction is to take the inverse Fourier transform of (2), which yields the elegant and concise formula

$$\beta_+^\alpha(t) = \frac{\Delta_+^{\alpha+1} t_+^\alpha}{\Gamma(\alpha+1)}, \qquad (3)$$

with $t_+^\alpha = \max(0,t)^\alpha$, which is compatible with Schoenberg's initial B-spline definition for α integer (Schoenberg, 1946). These functions are shown in Fig. 1.

Last but not least, we can apply a similar Fourier technique to derive an explicit formula for the fractional derivative of a B-spline

$$\partial^\gamma \beta_+^\alpha(t) = \Delta_+^\gamma \beta_+^{\alpha-\gamma}(t), \qquad (4)$$

which yields a fractional spline of reduced degree $\alpha - \gamma$. If we take $\gamma = \alpha + 1$, we hit the spline singularities and are left with a stream of Dirac impulses

$$\partial^{\alpha+1}\beta_+^\alpha(t) = \Delta_+^{\alpha+1}\delta(t) = \sum_{k=0}^{+\infty}(-1)^k \binom{\gamma}{k} \delta(t-k).$$

The whole point here is that there is a very simple, convenient calculus for taking the fractional derivatives of the fractional B-splines, and, by extension, of any function (spline or wavelet) that can be expressed as a linear combination of such basic atoms.

2.3 Fractional splines

Following Schoenberg (1946) once again, we call "cardinal fractional spline of degree α" any function $s(t)$ that can be written as

$$s(t) = \sum_{k\in\mathbb{Z}} c_k \beta_+^\alpha(t-k) \qquad (5)$$

with $c_k \in \ell_\infty$. Such a function has a unique and stable representation in terms of its B-spline coefficients c_k. Another more general way of defining fractional splines is to consider that the application of the fractional differential operator $\partial^{\alpha+1}$ to $s(t)$, as defined by (5), yields a weighted stream of Diracs. Thus, we will say that $s(t)$ is a fractional spline of degree α and knots sequence $-\infty < \cdots < t_0 < t_1 < \cdots t_k < +\infty$ if and only if:

$$\partial^{\alpha+1} s(t) = \sum_{k\in\mathbb{Z}} a_k \delta(t-t_k)$$

with $a_k \in \ell_\infty$.

2.4 Fractional spline wavelets

A basic fractional spline wavelet

$$\psi(t/2) = \sum_{k\in\mathbb{Z}} q_k \beta_+^\alpha(t-k)$$

can be specified by selecting a suitable sequence q_k such that $\langle\psi(\cdot/2), \beta_+^\alpha(\cdot - k)\rangle$ for all $k \in \mathbb{Z}$ (Unser et al., 1993). Using such a prototype, it is then possible to specify a basis of L_2 which takes the form $\{\psi_{i,k}\}_{i\in\mathbb{Z},k\in\mathbb{Z}}$, where $\psi_{i,k}(t) = 2^{-i/2}\psi(t/2^i - k)$. The remarkable property is that these wavelets essentially behave like multiscale

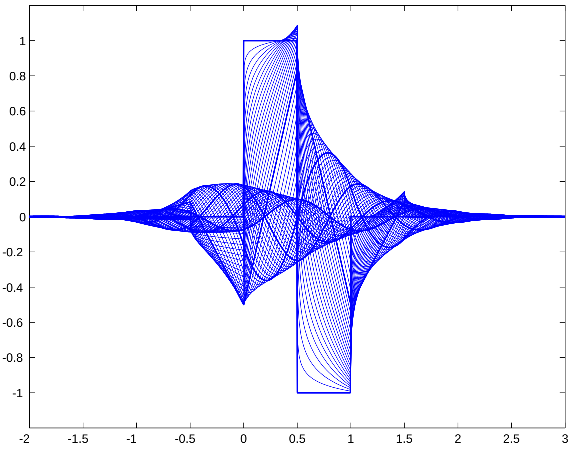

Fig. 2. Fractional B-spline wavelets with $\alpha \geq 0$ corresponding to the B-splines in Fig. 1. The remarkable property is that any one of these functions generates a wavelet basis of L_2.

fractional differentiation operators of order $\gamma = \alpha + 1$. Specifically, we have that

$$\langle\psi\left(\frac{\cdot - b}{a}\right), f(\cdot)\rangle = \partial^\gamma \left(\phi_a * f\right)(b)$$

with $\phi_a(t) = \phi(t/a)$, where $\phi(t)$ is a suitable smoothing kernel (typically, a fractional spline of degree $2\alpha+1$). The mathematical justification for this result can be found in (Unser and Blu, 2003).

The semi-orthogonal B-spline wavelets are shown in Fig. 2. The first one with $\alpha = 0$ is piecewise constant; it corresponds to the classical Haar transform. Likewise, the functions that are represented in thicker lines are piecewise polynomial and have been characterized by us more than a decade ago Unser et al. (1993). The ones in thinner lines are much less conventional in that they are truly fractional (Unser and Blu, 1999).

3. APPLICATIONS

The key advantages of fractional splines for applications in signal and image processing are threefold.

- Explicit fractional differentiation: It is possible to obtained closed-form expressions, and hence efficient computational algorithms, for computing any fractional derivative of a signal expressed in a fractional spline or wavelet basis.
- Family of adjustable wavelets: The fractional B-spline wavelets are tunable in a continuous fashion. By varying α, we have a direct control over a number of key wavelet properties: the parametric form of the basis functions, their smoothness, their space-frequency localization, the order and multiscale differentiability properties of the transform, and,

finally, the number of vanishing moments. The parameter α also directly controls the size (i.e., the spatial extent) of the basis functions. For instance, for the B-spline family, the basis functions (resp., wavelets) converge to Gaussians (resp., modulated Gaussians or Gabor functions) with a standard deviation (or equivalent window size) that is proportional to $\sqrt{\alpha}$ (Unser et al., 1992). This also means that these functions, for α sufficiently large (say, $\alpha > 2$), will tend to be optimally localized in the sense of the Heisenberg uncertainty principle; in other words, the product of their time and frequency uncertainties will tend to the minimum that is achievable.

- Fast algorithms: All fractional spline wavelet transforms can be implemented efficiently in the Fourier domain with an $O(N \log N)$ complexity, where N is the size of the signal (Blu and Unser, 2000). A further advantage is that the algorithm is completely generic; it is the same irrespective of the choice of α. The software is available on the web at: `bigwww.epfl.ch/demo/fractsplines/`

An example of application where the explicit differentiation property is particularly useful is tomographic image reconstruction from projections. Indeed, the first computational step of the filtered-backprojection algorithm is the evaluation of the so-called ramp filter that corresponds to a certain kind of (symmetric) fractional derivative for which one can also define fractional B-splines (Unser et al., 2000). Note that it is also possible to extend the B-spline family for dealing with an even larger family of fractional derivatives of the form $(j\omega)^{\frac{\gamma}{2}+\tau}(-j\omega)^{\frac{\gamma}{2}-\tau}$(Blu and Unser, 2003). In particular, this enlarged family includes the Hilbert transform—an important tool in signal processing, especially in the context of amplitude and frequency modulation. Other applications where the fractional differentiation properties are relevant are the wavelet-based generation of fractional Brownian motion-like processes (Meyer et al., 1999), and the analysis of signals with fractal properties (Flandrin, 1992). In particular, we note that a γth-order wavelet will essentially whiten processes whose power spectrum decays like $O(\omega^{-\gamma})$.

The fact that the fractional wavelets are adjustable in a continuous manner has been used advantageously for the statistical analysis of functional magnetic resonance (fMRI) data (Feilner et al., 2000). The idea here is to tune the wavelet transform to optimize the detection of activation patterns. Interestingly, it has been found that the value $\alpha = 1.2$ has a special significance since it establishes a relation between the effect of downsampling and the notion of resel used in the standard SPM approach which applies a Gaussian prefilter to the data (Van De Ville et al., 2003). Another promising application is seismic imaging. Herrmann argues that the fractional discontinuities of the type $(t - t_0)_+^{\alpha}$ (or $(t_0 - t)_+^{\alpha}$) are well matched to the description of seismic data. He has developed a fractional spline wavelet-packet-like algorithm for analyzing these signals (Herrmann, 2001).

REFERENCES

G. Battle. A block spin construction of ondelettes. Part I: Lemarié functions. *Commun. Math. Phys.*, 110:601–615, 1987.

T. Blu and M. Unser. The fractional spline wavelet transform: Definition and implementation. In *Proc. Int. Conf. Acoustics, Speech, and Signal Processing*, volume I, pages 512–515, Istanbul, Turkey, June 5-9, 2000.

T. Blu and M. Unser. A complete family of scaling functions: The (α, τ)-fractional splines. In *Proc. Int. Conf. Acoustics, Speech, and Signal Processing*, volume VI, pages 421–424, Hong Kong SAR, People's Republic of China, April 6-10, 2003.

M. Feilner, T. Blu, and M. Unser. Optimizing wavelets for the analysis of fMRI data. In *Proc. SPIE Conf. Wavelet Applications in Signal and Image Processing VIII*, volume 4119, pages 626–637, San Diego CA, USA, July 31-August 4, 2000.

P. Flandrin. Wavelet analysis and synthesis of factional Brownian motion. *IEEE Trans. Information Theory*, 38(2):910–917, 1992.

F.J. Herrmann. Singularity characterization by monoscale analysis: Application to seismic imaging. *Applied Computational Harmonic Analysis*, 11(1):64–88, 2001.

Y. Meyer, F. Sellan, and M.S. Taqqu. Wavelets, generalized white noise and fractional integration: The synthesis of fractional brownian motion. *J. Fourier Analysis and Applications*, 5 (5):465–494, 1999.

I.J. Schoenberg. Contribution to the problem of approximation of equidistant data by analytic functions. *Quart. Appl. Math.*, 4:45–99, 112–141, 1946.

M. Unser, A. Aldroubi, and M. Eden. On the asymptotic convergence of B-spline wavelets to Gabor functions. *IEEE Transactions on Information Theory*, 38(2):864–872, March 1992. Part II.

M. Unser, A. Aldroubi, and M. Eden. A family of polynomial spline wavelet transforms. *Signal Processing*, 30(2):141–162, 1993.

M. Unser and T. Blu. Construction of fractional spline wavelet bases. In *Proc. SPIE Conf. Wavelet Applications in Signal and Image Processing VII*, volume 3813, pages 422–431, Denver CO, USA, July 19-23, 1999.

M. Unser and T. Blu. Fractional splines and wavelets. *SIAM Review*, 42(1):43–67, 2000.

M. Unser and T. Blu. Wavelet theory demystified. *IEEE Trans. Signal Process.*, 51(2):470–483, February 2003.

M. Unser, S. Horbelt, and T. Blu. Fractional derivatives, splines and tomography. In *Proc. European Signal Processing Conference*, volume IV, pages 2017–2020, Tampere, Finland, September 4-8, 2000.

D. Van De Ville, T. Blu, and M. Unser. Wavelets versus resels in the context of fMRI: Establishing the link with SPM. In *Proc. SPIE Conf. Wavelet Applications in Signal and Image Processing X*, volume 5207, pages 417–425, San Diego CA, USA, August 3-8, 2003. Part I.

V.A. Zheludev. Fractional-order derivatives and the numerical solution of certain convolution equations. *Diff. Equations*, 18:1404–1413, 1983.
