# Supplementary figures and images for: Rhythms and Background (RnB): The Spectroscopy of Sleep Recordings
Source: eNeuro. 2026 Feb 3;13(2):ENEURO.0235-25.2025. doi: 10.1523/ENEURO.0235-25.2025 (PMC12867552; doi:10.1523/ENEURO.0235-25.2025)

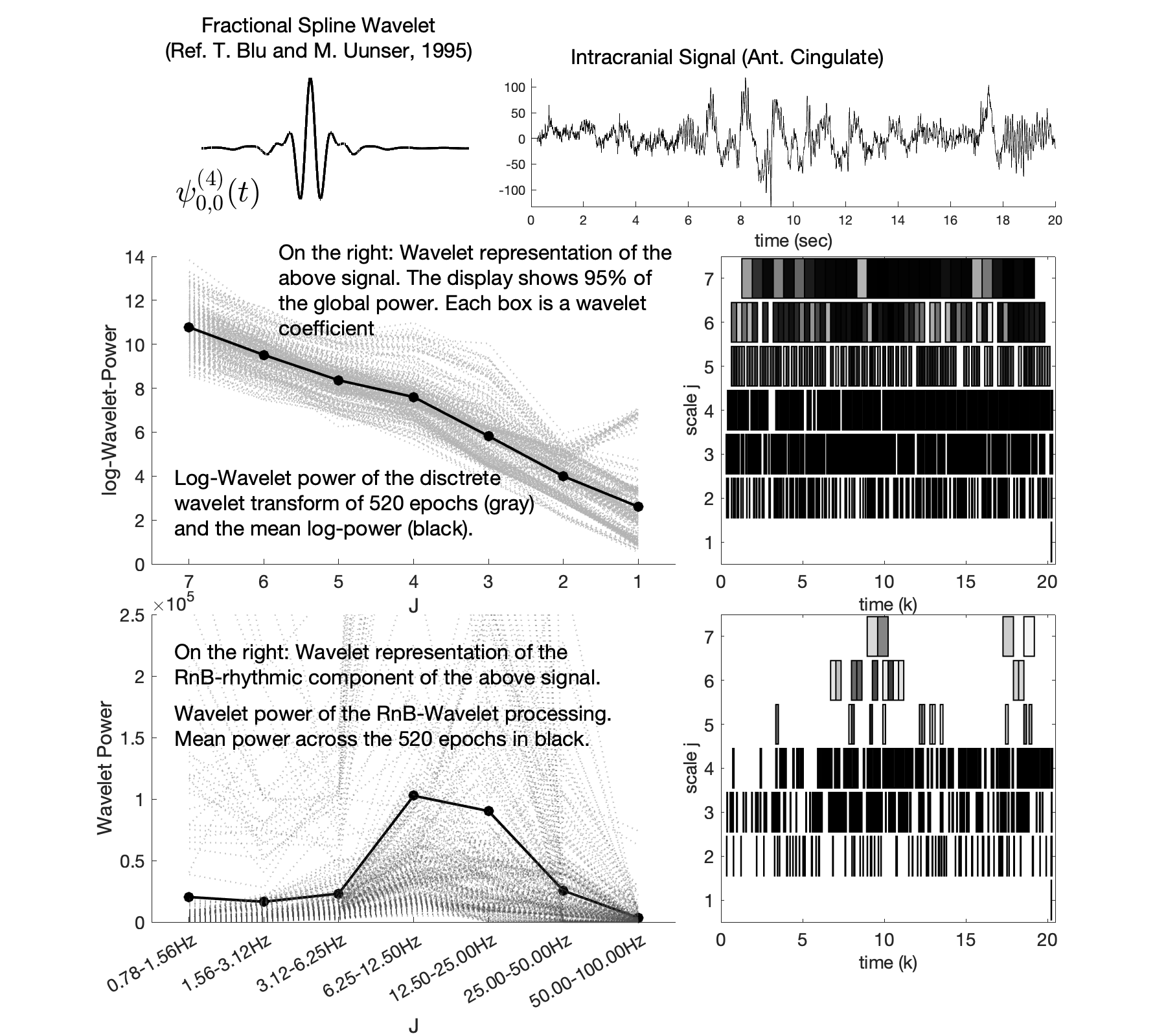

Supplement: Data 1 — Download Data 1, ZIP file. [file eneuro-13-ENEURO.0235-25.2025-s002.zip › RnB-Wavelet-main/Figures/FigGitHub.png]
